# “EviMass”: A Literature Evidence-Based Miner for Human Microbial Associations

**DOI:** 10.3389/fgene.2019.00849

**Published:** 2019-09-13

**Authors:** Divyanshu Srivastava, Krishanu D. Baksi, Bhusan K. Kuntal, Sharmila S. Mande

**Affiliations:** ^1^Bio-Sciences R&D Division, TCS Research, Tata Consultancy Services Ltd., Pune, India; ^2^School of Information Technology, Indian Institute of Technology Delhi, Delhi, India; ^3^Chemical Engineering and Process Development Division, CSIR-National Chemical Laboratory, Pune, India; ^4^ Academy of Scientific and Innovative Research (AcSIR), Ghaziabad, India

**Keywords:** microbiome, literature mining, human disease, web server, microbial association

## Abstract

The importance of understanding microbe–microbe as well as microbe–disease associations is one of the key thrust areas in human microbiome research. High-throughput metagenomic and transcriptomic projects have fueled discovery of a number of new microbial associations. Consequently, a plethora of information is being added routinely to biomedical literature, thereby contributing toward enhancing our knowledge on microbial associations. In this communication, we present a tool called “EviMass” (Evidence based mining of human Microbial Associations), which can assist biologists to validate their predicted hypotheses from new microbiome studies. Users can interactively query the processed back-end database for microbe–microbe and disease–microbe associations. The EviMass tool can also be used to upload microbial association networks generated from a human “disease–control” microbiome study and validate the associations from biomedical literature. Additionally, a list of differentially abundant microbes for the corresponding disease can be queried in the tool for reported evidences. The results are presented as graphical plots, tabulated summary, and other evidence statistics. EviMass is a comprehensive platform and is expected to enable microbiome researchers not only in mining microbial associations, but also enriching a new research hypothesis. The tool is available free for academic use at https://web.rniapps.net/evimass.

## Introduction

The microbial groups residing in human body remain in complex association within themselves as well as with the host. These associations range from mutualism, amenalism, and commensalism to parasitism, predation, and competitions (Faust and Raes, 2012). However, with the onset of a disease, the human microbiome is often seen to display aberrations, which may be a cause or an effect (Eloe-Fadrosh and Rasko, 2013; Liang et al., 2018). Advances in the field of metagenomics have made it possible to successfully capture and report such microbial dysbiosis observed in the diseased state. Microbial abundance measurements for many samples can be simultaneously obtained using 16S rRNA (amplicon) sequencing in a short span of time (Goodrich et al., 2014). Recent developments in sequencing technology and the drastic reduction in the associated cost have encouraged researchers to probe the microbial basis of various human diseases. Consequently, a plethora of information relating to microbes and their association with diseases are added to the growing biomedical literature (Cani, 2018). Although the obtained microbiome data can be used to calculate differentially abundant genera as well as their co-occurrence patterns (Kuntal et al., 2013; Kumar et al., 2014; Dhariwal et al., 2017), their evidence from biomedical literature can help to strengthen a research hypothesis.

The Human Microbe Disease Association Database (HMDAD) was the first resource developed using literature mining to systematically gather experimental data to study microbe–disease associations (Ma et al., 2017b). Several tools have been developed thereafter to utilize the curated data from HMDAD and score human microbe associations using advanced mathematical approaches (Chen et al., 2017; Huang et al., 2017a; Huang et al., 2017b; Wang et al., 2017b; Peng et al., 2018; Zou et al., 2018; Qu et al., 2019). The above set of tools focuses on identifying associated genera across a set of selected diseases and is eventually used to find diseases having similar pattern of associated microbes. For example, KATZHMDA (Chen et al., 2017) computes the number of walks of connections between microbe and disease nodes, LRLSHMDA (Wang et al., 2017b) uses a semisupervised learning framework based on Laplacian regularized least squares, ABHMDA (Peng et al., 2018) uses an Adaptive Boosting model, PBHMDA (Huang et al., 2017b) calculates the Gaussian interaction profile kernel similarity, and very recently a new method called MDLPHMDA (Qu et al., 2019) based on Matrix Decomposition and Label Propagation has been introduced. While some of the above methods are limited to predict microbes associated with a fixed set of diseases, more recent methods like ABHMDA can predict microbes associated with a new disease (Peng et al., 2018). In addition, methods like MDLPHMDA also can now be used to predict novel microbe–disease associations with minimum noise (Qu et al., 2019). Tools like Micro-pattern, on the other hand, can perform an enrichment analysis for a given set of microbes using a hypergeometric test (Ma et al., 2017a). This method relies on creation of pregenerated microbe sets using manual curation from selected diseases, making it advantageous for accurate predictions, but limits the applicability. Given the scenario, although the association of individual microbes with a disease can give informative predictions, the knowledge of microbial co-occurrence patterns can augment it further to provide improved insights. As microbes are known to work in mutual associations rather than single entities, it is also imperative to validate a known co-occurrence pattern observed in an experimental microbiome study. One such method called “Microbial Prior Lasso” (or MPLasso) uses literature evidence supplied as an input to quantify microbial associations and is available as an R package (Lo and Marculescu, 2017). However, the major limitation lies in gathering systematic information relating to intermicrobe association and their relation to human diseases.

**Figure 1:**
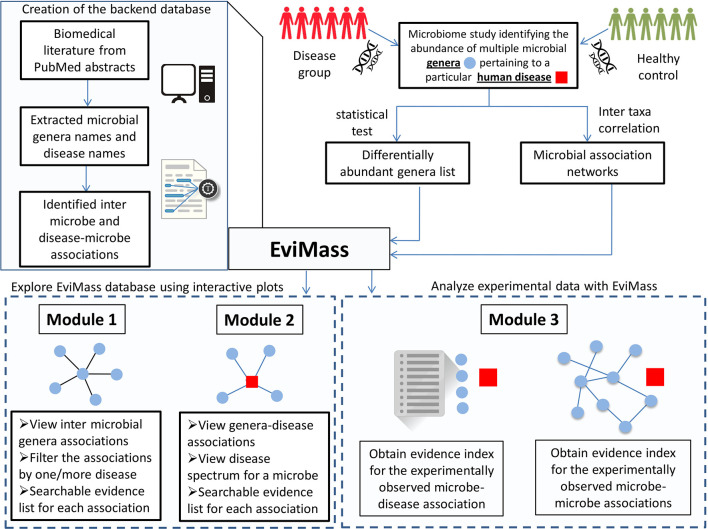
Overview of the EviMass backend creation and utility of its various modules in understanding the intermicrobial and microbe–disease associations.

In order to address the aforementioned limitation, we have developed a web-based GUI resource called “EviMass” (Evidence based mining of human Microbial Associations) available at https://web.rniapps.net/evimass that can be interactively used for not only querying microbe disease associations, but also inferring the intermicrobe association patterns mined from biomedical literature (**Figure 1**). The EviMass backend database has been developed using extensive data mining of the currently available PubMed abstracts. The front-end is designed with an interactive query system, which allows users to find all microbes associated with a user-defined query microbe. In addition, the identified microbial associations can also be visualized for their occurrence statistics in various human diseases. Similarly, users can search for an individual microbe to view all diseases associated with it and vice versa. Additionally, users can upload a microbial association network generated from experimental microbiome data corresponding to a human disease and easily verify these associations using the evidence statistics. A list of differentially abundant genera obtained from a disease–control microbiome case study can also be validated using EviMass along with an option for enrichment analysis. All evidence inferred using the present tool is listed with corresponding PubMed IDs, which can be used for further reference. The utility of EviMass is demonstrated with case studies as well as using real-world microbiome data.

## Results

### Global Overview of Disease–Microbe Associations Captured by EviMass

**Figure 2:**
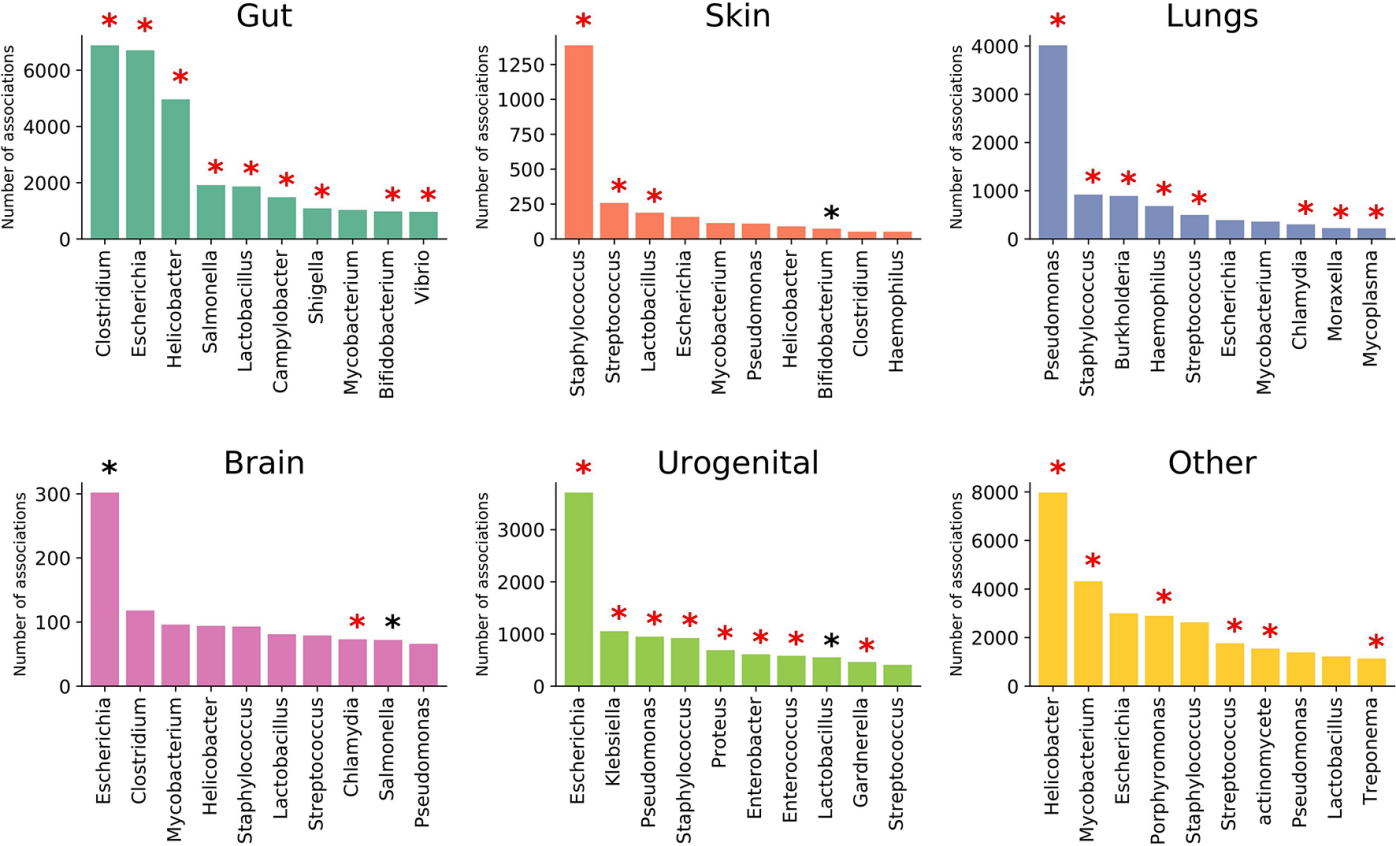
Top 10 prominent microbial genera associated with diseases affecting various organs. Statistically significant (*P* < 0.05) genera are marked with a black asterisk (with Bonferroni-corrected *P* < 0.05 highlighted in red).

EviMass backend database was generated using a systematic literature mining approach (details in *Material, Methods and Implementation*) specific to microbiome and human diseases. We focused our analysis on 51 widely reported microbiome associated human diseases and their associations with various microbes (genera level). These diseases spanned six categories, namely, systemic diseases and those affecting gut, skin, lung, brain, and urogenital system (**Table 1**). The results of the literature mining as incorporated in EviMass yielded several interesting findings. For example, ulcer, diarrhea, HIV, urinary tract infection, and cystic fibrosis were found to be the most widely (top 5) reported diseases with microbial associations (**Supplementary Figure 1**). On the other hand, microbial genera, namely, *Escherichia*, *Staphylococcus*, *Pseudomonas*, *Bacillus*, and *Streptococcus*, were seen to occupy the top 5 spots in terms of their reported all-microbiome articles in PubMed (irrespective of disease association) (**Supplementary Figure 2**). A closer look into the genera maximally associated with human diseases revealed *Escherichia*, *Lactobacillus*, *Clostridium*, *Streptococcus*, and *Bacteroides* to be the top 5 players (**Supplementary Figure 3**). A deeper analysis revealed the following genera to be significantly (*P* < 0.05) associated with diseases (affecting various organs): *Clostridium* with gut, *Staphylococcus* with skin, *Pseudomonas* with lungs, *Escherichia* with brain as well as urogenital, and *Helicobacter* with the other systemic diseases (**Figure 2**).

**Table 1:** List of different microbe-related human diseases categorized by the organs they affect.

Organs affected	Diseases	No. of diseases
Gut	End-stage renal disease (ESRD), kidney stones, diarrhea, liver cirrhosis, malnutrition, ileal Crohn disease (CD), necrotizing enterocolitis, colon cancer, infectious colitis, constipation, colitis, ulcerative colitis, Whipple disease, irritable bowel syndrome (IBS), gastroesophageal reflux, Crohn disease (CD), gastric and duodenal ulcer, inflammatory bowel disease (IBD), *Clostridium difficile* infection (CDI), colorectal carcinoma	20
Skin	Skin and mucosal infections, atopic dermatitis, psoriasis, guttate psoriasis, atopic sensitization, eczema, atopy	7
Lungs	Asthma, allergic asthma, recurrent wheeze, chronic obstructive pulmonary disease, cystic fibrosis	5
Brain	Multiple sclerosis, Parkinson’s disease, Schizophrenia, Autism, Depression	5
Urogenital	Urinary tract infection, bacterial vaginosis, polycystic ovary syndrome, preterm birth	4
Systemic	Type 1 diabetes, diabetes, type 2 diabetes, HIV/AIDS, obesity, systemic inflammatory response syndrome, allergic sensitization, allergy, ulcer, periodontitis	10
	Total	51

**Figure 3:**
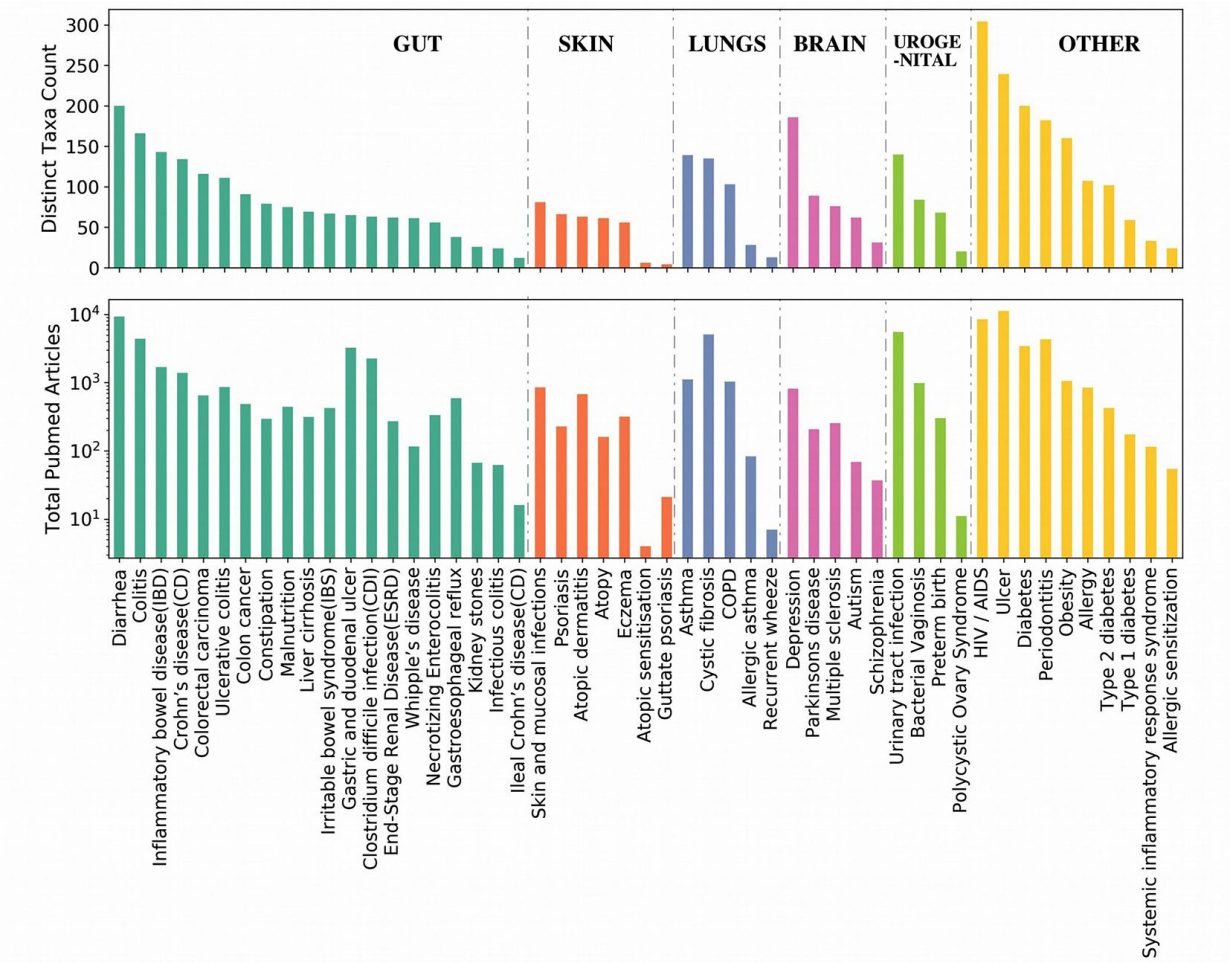
Summary of the associated microbial genera count corresponding to each disease and the number of articles reporting the disease. The diseases are ordered based on the categories as listed in **Table 1**. Each category of disease is sorted based on the number of genera associations.

In order to check which all genera are closely associated with each of the aforementioned top genera irrespective of diseases, Module 1 of the EviMass tool was utilized. The results (**Supplementary Figures 4–19**) showed a wide range of association patterns between each of these genera shown as graphs. While the central node of the graph represented the query genera, the remaining nodes corresponded to the genera associated with it. The size of the nodes depended on the strength of the associations calculated as the sum total of publications where the two genera were identified to co-occur. It was interesting to observe that most of the association graphs were dominated by a selected group of genera like *Escherichia*, *Staphylococcus*, and *Pseudomonas*. In order to get a deeper insight into the microbe–disease associations, a summary of the associated microbial genera count corresponding to each disease and the number of articles reporting the disease was generated (**Figure 3**). The Module 2 of EviMass was then used to explore each of these associations along with the literature evidences. Our analysis using EviMass for the top diseases across each category showed some amount of genera specificity (**Supplementary Figures 20–24**). For example, cystic fibrosis (**Supplementary Figure 20**) showed a very strong association with the genera *Pseudomonas* with 3,711 evidences (journal articles). Apart from being dominant in cystic fibrosis, *Pseudomonas* was also found to be associated with other diseases like HIV, diabetes, ulcer, and urinary tract infection although with lower evidences. Similar associations were also observed in other diseases (**Supplementary Figures 20–24**), which instigated an interest to look into the disease similarities based on their associated genera as explored in the next section.

### Disease Similarity Based on Literature Evidence Using EviMass

**Figure 4:**
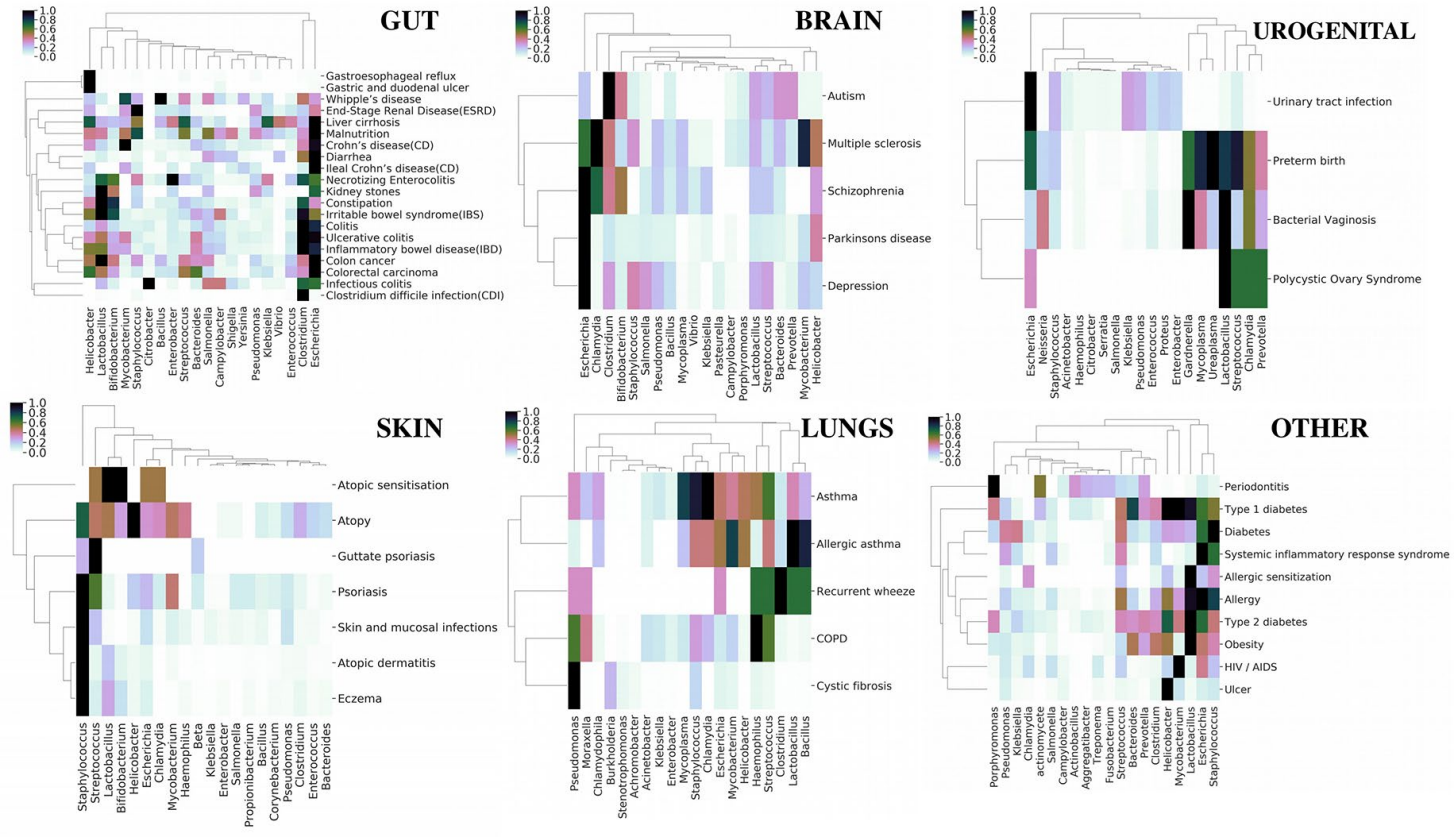
Category-wise (organs affected by various diseases) bidirectionally clustered heat maps based on microbial associations. The top 20 persistent microbes across the six categories (**Table 1**) were chosen and used to generate bidirectionally clustered (UPGMA hierarchical clustering) heat map for each category. Euclidean distance was used as the measure of distance, and the values were normalized by rows (diseases).

Although earlier studies (Ma et al., 2017a) have shown an overall relation between various diseases based on their microbial associations, we focused on obtaining categorical insight based on our extended database (**Figure 4**). The top 20 persistent microbes across the six categories (**Table 1**) were chosen and used to generate bidirectional clustered (UPGMA hierarchical clustering) heat map for each category. Euclidean distance was used as the measure of distance, and the values were normalized by rows (diseases). Diseases like colorectal carcinoma, colon cancer, inflammatory bowel disease, irritable bowel syndrome, colitis, and kidney stones were part of closely linked cluster in the gut category. These diseases were seen to be reported with an increased association with *Lactobacillus*, *Bifidobacterium*, and *Clostridium*. The skin, brain, and urogenital diseases did not show any distinct clustering, but *Staphylococcus*, *Escherichia*, and *Lactobacillus* were observed to be the dominant players in these diseases, respectively. Asthma and related diseases were seen to cluster away from cystic fibrosis and chronic obstructive pulmonary disease in the lung category. The remaining category of systemic diseases showed a clear cluster of allergy, obesity, and type 2 diabetes dominated by *Lactobacillus* and *Helicobacter*. Periodontitis, one of the diseases in the last category, clustered away from other systemic diseases and was characterized by the increase in association of the genera *Porphyromonas*. The “word cloud” feature was used to understand the associations that distinctly showed the dominance of the word “gingivalis” (**Supplementary Figure 25**) in the abstracts indicating the role of *Porphyromonas gingivalis*. A secondary search on the listed abstracts by using the keyword “inflammation” further yielded keywords like “cytokines,” “tnf,” “lps” (**Supplementary Figure 25**), which are indicators of some mechanisms of *Porphyromonas gingivalis* infection in periodontitis (Jiang et al., 2018; Kajiura et al., 2018; Zhou et al., 2018). However, these observations only provided a global picture, which can be enriched by augmenting with experimental data. In the next section, we investigated a specific disease along with reported experimental data to get more insights into microbial pathogenesis.

### Case Study With Real World Microbiome Data

One of the featured utility of the EviMass tool pertains to Module 3, which allows users to validate their results from microbiome experiments based on the curated literature evidence. In order to demonstrate the utility, we first selected a publicly available data (Fazlollahi et al., 2018) where the authors studied 72 asthma subjects (using 16S ribosomal RNA sequencing on nasal swabs) and compared the same with those obtained from healthy controls. Four microbial genera reported to be significantly associated with asthma, namely, *Prevotella*, *Dialister*, *Gardnerella*, and *Alkanindiges*, were used as input for the EviMass Module 3 along with the disease keyword “asthma.” The result indicated *Prevotella* to be the most widely reported as well as statistically significant (*P* < 0.001) genera to be associated, among others, for asthma (**Supplementary Figure 26**). The node “*Prevotella*” can be clicked to populate the list of PubMed articles reporting the association, which in turn can be filtered based on search criteria. As most microbes are known to orchestrate an inflammatory disease by altering the immune response in the host, we searched for the keyword “immune” to filter the articles reporting the immunological role of *Prevotella* in asthma. The search result yielded three articles, of which one clearly reported the marked capacity of *Prevotella* in driving T_H_17 immune responses (Larsen, 2017).

In the next step, we used another dataset for analyzing a microbial association network for allergic asthma where the authors did not find any differentially abundant genera specific to the allergy samples (Hevia et al., 2016). We had used the same data in one of our earlier works (Kuntal et al., 2019) to identify microbial “driver” genera (using “NetShift” methodology). While *Granulicatella* and *Turicibacter* were seen to be two potential pathogenic drivers, only *Granulicatella* was predicted to be the main driver (Kuntal et al., 2019). The same microbial network was used as an input for EviMass, and the associations of *Granulicatella* and *Turicibacter* were investigated with Module 3 (also provided as an autoload example in the web server). The evidence statistics for *Granulicatella* and its associated genera (which were mostly pathogens) *Staphylococcus*, *Streptococcus*, and *Veillonella* showed a tendency to co-occur irrespective of disease condition (**Supplementary Figure 27**). For example, evidence for association of *Granulicatella* and *Staphylococcus* was seen in 23 articles, *Granulicatella* and *Streptococcus* in 80 articles, and *Granulicatella* and *Veillonella* in 35 articles. This observation provides evidence that co-occurrence of the genus *Granulicatella* with the above pathogens is indeed seen globally. On the other hand, the associations of *Turicibacter* (with *Fusibacter* and *Alkaliphilus*) did not show any literature evidence of co-occurrence (**Supplementary Figure 28**), thereby strengthening our earlier prediction of inability of *Turicibacter* to become a pathogenic driver. The primary intention of this case study was to demonstrate the ease with which scientific hypothesis in microbiome research can be enriched using the EviMass tool.

## Conclusions and Future Work

In this communication, we developed a resource for understanding the microbe–microbe and microbe–disease associations. The present version aims to provide a one-stop platform for validating data-driven hypothesis on microbiome studies. We aim to update our resource on a regular basis in order to incorporate the growing corpus of information. The current version of EviMass performs a text processing of the available PubMed abstracts to identify microbe association trends (increase or decrease). Additionally, it allows one to filter the results based on specific queries like genera/species name, journal information, or any generic keyword available in the abstracts. While interpreting the results, it should be noted that the association graphs are generated based on the cumulative evidence counts, which might be biased for a disease or microbe having a higher coverage. In such cases, the individual associations must be carefully assessed using the implemented hypergeometric tests before making any biological inference. The implementation of word cloud for the search output can highlight keywords in the abstracts that get repeatedly mentioned. Although this feature can be used as a tool to understand the mechanism of how the microbes affect various diseases, it is strongly advised to carefully crosscheck with the individual publications. In a future update, we plan to link the results with human genome-wide association studies and other related databases to help users automatically get improved insights. We also plan to augment an additional layer of natural language processing to help users automatically get insights on the nature of interaction in a future update. Additionally, we will introduce a “Contribute” feature to allow users pick a random abstract from an initial preselected set of abstracts and submit their annotation on the observed type of association (both microbe–microbe and microbe–disease). Every annotation will be cross validated by two other independent annotations to improve accuracy. We expect EviMass to serve as a valuable resource for microbiologist as well as other researchers working in the field of human microbiome and diseases.

## Material, Methods and Implementation

### Data Acquisition and Building the EviMass Backend

**Figure 5:**
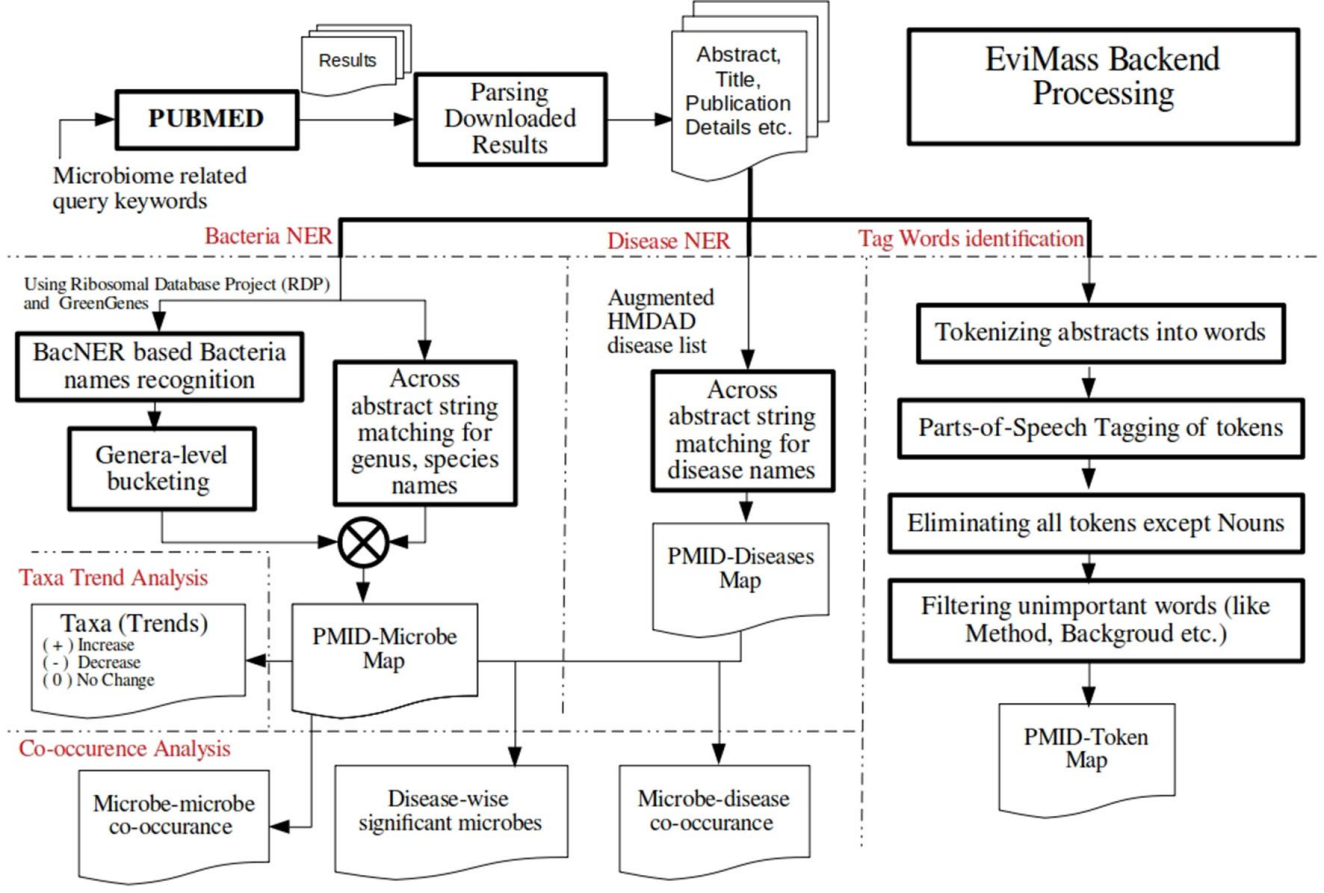
Flowchart describing the various steps involved in development of the EviMass backend.

Generation of the EviMass backend involved two major steps, namely, information extraction and entity recognition. Articles with abstracts were downloaded directly from PubMed. A combination of keywords including “microbe,” “microbiome,” “microbial disease,” “metagenome,” and “bacteria” was used to query abstracts using the PubMed web interface. There were 1,457,991 unique articles retrieved, which were parsed using in-house scripts to retain PubMed IDs, title, publication year, journal name, authors, and abstract text. These abstracts were further processed to extract bacteria names and the reported human diseases. The steps involved in backend processing are described below as well as summarized in **Figure 5**. Processed backend tables along with their description are provided in the **Supplementary data**.

### Bacteria Named Entity Recognition

The abstracts were passed through a named entity recognition (NER) engine implemented in the BacNER tool (Wang et al., 2017a). BacNER is a dedicated bacterial NER tool, which reports bacteria names, strains, and related entities from a given query text. It is based on a trained conditional random field, which processes text and tags bacterial entities in IOB (inside-outside-beginning) format. The title and the abstract for each article were passed to BacNER, and the entities reported in them were extracted. A total of 787,069 articles from our library were returned with at least one bacterial entity recognized. The results from BacNER required further processing in order to be used in our model. For instance, entities like *Escherichia coli* and *E. coli* needs to be clubbed together. Moreover, there were instances where specific species/strains of a bacterium were reported, which needed to be clustered together. The identified species were also kept as a separate map with the PubMed IDs to display them in the EviMass web tool. To resolve these ambiguities, a master list of 2,178 genera was generated using the Ribosomal Database Project (Maidak et al., 1996) and Green Genes (DeSantis et al., 2006) database. As the majority of microbiome 16S rRNA studies utilize one of these databases, it also aligns to our aim of validating the results from microbiome experimental data. Using an approximate string matching method based on Levenshtein distance (Miller et al., 2009), each identified bacterial entity was matched and mapped to the master list. The mapping was then manually verified to modify inconsistent mappings. A total of 637,428 articles were finally selected having a mapped bacterial entity to the biomedical text. A detailed description of the steps involved is summarized in **Figure 5**.

### Diseases Named Entity Recognition

In order to create a disease entity dictionary, the HMDAD’s most commonly occurring list of diseases (Ma et al., 2017a) was used along with some additions to finalize a set of 51 diseases. The disease set is created in order to effectively cater to the wide variety of researches. For example, “diabetes” is deliberately kept as a different disorder and is not merged with “type 1” or “type 2 diabetes.” Another example of a similar case pertains to the disease “colorectal carcinoma” where we added a search query term for both “colorectal carcinoma” and “colorectal cancer” to encompass all the search results. These 51 diseases were further grouped into 6 categories broadly based on their target regions: gut, skin, lungs brain, urogenital, and other (systemic diseases) (**Table 1**). Disease names were recognized from abstracts identified earlier to have an associated bacterial entity using string matching.

The complete information extracted from more than a million scientific articles is stored and indexed for minimum memory consumption and fast access. All the genera as well as the diseases reported for articles are stored in tables, where each record corresponds to a PubMed ID. Apart from this, all PubMed IDs that report each genus are also separately identified and stored. Similarly, a mapping of disease and PubMed IDs is also created for easy information retrieval. Abstracts are then processed to identify “increase” and “decrease” of the various microbial names identified to be present in them. These patterns are later displayed in the web application in the PMID result table under “taxa and trends” column with a “+” (increase), “−” (decrease), and 0 (no trend detected) sign beside the identified taxa name in an abstract. For advanced analysis, EviMass holds all the parts-of-speech (POS) tagged noun words corresponding to the articles, which can be used to get a deeper insight. These POS tags can be used to fine tune a search based on a particular term of interest as described in the next section (**Figure 5**). Microbial genera significantly associated with the diseases (*P* < 0.05) were identified using a Fisher exact test (Lo and Marculescu, 2017; Ma et al., 2017a), which is further applied for enrichment analysis in the web tool.

### The EviMass Frontend

EviMass web server uses the generated backend to allow easy queries using simplistic searches and graphical outputs. Three workflows are implemented to systematically query for a microbe–microbe or disease–microbe association as described below (additional details in **Supplementary material 2**).

### Module 1: Identify Intermicrobial Associations

Using this module, users can select a microbial genus and find all other microbial genera associated with it. The results of the workflow are presented as a network with the central node representing the queried genus and the peripheral nodes representing the associated genera. The sizes of the nodes represent the strengths of the associations and are calculated as the total number of publications where the two genera (corresponding to the central and the peripheral node) are identified to co-occur. EviMass displays the top 100 strongest associated pairs by default but also provide users an option to view all the associations. Along with the network, a dropdown/text box with automatic suggestions for associated microbial genera names is rendered. Clicking on any node or selecting any microbial genera from the dropdown will display all the PMIDs in which the corresponding genera and the queried genera co-occur, along with the main keywords (POS tags) used in the abstract listed as a table. Additionally, a set of hypergeometric tests, namely, Fisher exact test and χ^2^ test, are performed (Camilli, 1995; Lo and Marculescu, 2017; Ma et al., 2017a) to statistically assess the significance of the selected association, and the results are presented as a contingency table along with *P* values. Users have the option to search and filter the displayed table for any term/keyword and narrow down the number of abstracts containing the specified word using either the global search or a column-specific search. Also, to ease further analysis, a word cloud of entity names in the abstracts from the PMID resultant output table can be generated for a specific custom query. If a particular gene, protein, or clinical condition gets repeatedly mentioned in the abstract texts for the selected interaction, it will appear as a dominant word. The PMID output table can also be downloaded in a variety of commonly used formats. EviMass also allows users to identify inter microbial associations, which are present in a selected set of diseases using interactive options.

### Module 2a: Identify All Microbial Genera Associated With a Disease

This module can be used to find all genera that are reported to be associated with a selected disease. The results of this module can be viewed either as a network (with the central node being the disease and the peripheral nodes being the associated microbial genera) or as a bar chart with the top 30 associated genera sorted by their strength of associations. Microbes identified to be significantly (*P* < 0.05) associated with the selected diseases are highlighted in pink (nodes/bars). In addition, a dropdown/text box with automatic suggestions for associated microbial genera names is provided for convenience. Clicking on any peripheral node (in case of the network view) or bar (in case of bar chart) or selecting any microbial genus from the dropdown displays the PMIDs in which the disease and the corresponding genus co-occur along with the keywords in the abstract in a sortable, searchable, and downloadable table. Similar to Module 1, results for assessing significance of the association are also generated. In addition, a genus node can be interactively queried (using left mouse click) to inquire its other known disease associations as a separate bar plot.

### Module 2b: Identify All Diseases Associated With a Microbial Genera

The diseases associated with a particular genus can be evaluated using this module. A genus can be queried to find its associations with the diseases depicted in form of a bar chart, arranged in order of the strength of their associations along with their statistical significance. As in the previous modules, clicking on any bar or selecting any associated disease from the dropdown will load the PMIDs where the corresponding disease and the queried microbe co-occur along with the keywords in a sortable, searchable, and downloadable table. The “word cloud” for the PMID resultant table can be used to understand the mechanism of how the microbe affects the disease (**Supplementary Figure 25**).

### Module 3a: View Literature Evidence for a Disease-Specific Microbial Network

Often, biological systems are analyzed as a network/graph, which are mostly obtained using computational techniques on microbiome abundance data. However, such data-driven approaches often lead to spurious connections among noninteracting microbes, due to either measurement or statistical errors. Therefore, a quick and easy method to correlate such associations with literature mined results is likely to help in getting an improved understanding. This module offers users the possibility to upload a microbial association network as an edge list along with the pertinent disease. The uploaded edge list is depicted as a network with a searchable dropdown containing all the edges. For user convenience, the edge widths are automatically mapped to their association frequencies. Clicking on any edge or selecting any edge from the dropdown shows the PMIDs and keywords where the pair co-occurs along with a list of evidence statistics. The evidence statistics reports the occurrence count of the selected genera independently as well as together in the given disease, any diseases, and globally in the EviMass backend. The utility of this feature has been demonstrated as a case study in the Results section.

### Module 3b: View Literature Evidence for Genera Identified to Be Differentially Abundant in a Disease and Perform Enrichment Analysis

Analyzing differentially abundant microbial genera in disease–healthy microbiome studies is often used to identify potential microbial biomarkers. This module enables one to view literature reported evidences for associations of a given set of differentially abundant microbial genera (identified from an experimental study) with a specific disease. The results of the module can be viewed either as a network, with the central node depicting the disease and the peripheral nodes representing the queried microbial genera, or as a bar chart with the queried genera sorted by their strength of associations. In addition, a dropdown/text box with automatic suggestions for associated microbial genera names is rendered. Clicking on any peripheral node (in case of the network view) or bar (in case of bar chart) or selecting any microbial genus from the dropdown displays the PMIDs in which the disease and the corresponding genus co-occur along with the keywords in the abstract in a sortable, searchable, and downloadable table. All the other disease associations of the genus corresponding to the selected node/bar are reported as a separate bar chart. An enrichment analysis of the uploaded set of microbial genera is performed with respect to the selected disease similar to the implementation in Micro-pattern (Ma et al., 2017a). For this implementation, the microbes identified to be significantly associated with the 51 diseases are used as “disease sets” in EviMass.

## Data Availability

Publicly available datasets were analyzed in this study. This data can be found here: https://web.rniapps.net/netshift/datasets/allergy.zip.

## Author Contributions

BK conceived the idea. DS extracted and processed/analyzed the data and created the backend. KB designed and developed the web server and implemented the statistical tests. BK, DS, and KB designed the case studies. BK, DS, KB, and SM evaluated the results and drafted the manuscript. All authors read and approved the final manuscript.

## Conflict of Interest Statement

All the authors are employed by company Tata Consultancy Services Limited and declare no conflict of interest.
