# Corrigendum: “EviMass”: A Literature Evidence-Based Miner for Human Microbial Associations

**DOI:** 10.3389/fgene.2020.614051

**Published:** 2020-11-02

**Authors:** Divyanshu Srivastava, Krishanu D. Baksi, Bhusan K. Kuntal, Sharmila S. Mande

**Affiliations:** ^1^Bio-Sciences R&D Division, TCS Research, Tata Consultancy Services Ltd., Pune, India; ^2^School of Information Technology, Indian Institute of Technology Delhi, Delhi, India; ^3^Chemical Engineering and Process Development Division, CSIR-National Chemical Laboratory, Pune, India; ^4^Academy of Scientific and Innovative Research (AcSIR), Ghaziabad, India

**Keywords:** microbiome, literature mining, Human Disease, Web server, microbial association

In the published article, there was an error in affiliation **4**. Instead of **“Academy of Scientific and Innovative Research (AcSIR), CSIR-National Chemical Laboratory Campus, Pune, India”** it should be “**Academy of Scientific and Innovative Research (AcSIR), Ghaziabad, India”**.

The authors apologize for this error and state that this does not change the scientific conclusions of the article in any way. The original article has been updated.

