# Supplementary material for: “EviMass”: A Literature Evidence-Based Miner for Human Microbial Associations
Source: Front Genet. 2019 Sep 13;10:849. doi: 10.3389/fgene.2019.00849 (PMC6763948; doi:10.3389/fgene.2019.00849)
Supplement: Supplementary file 1 [file DataSheet_1.pdf]

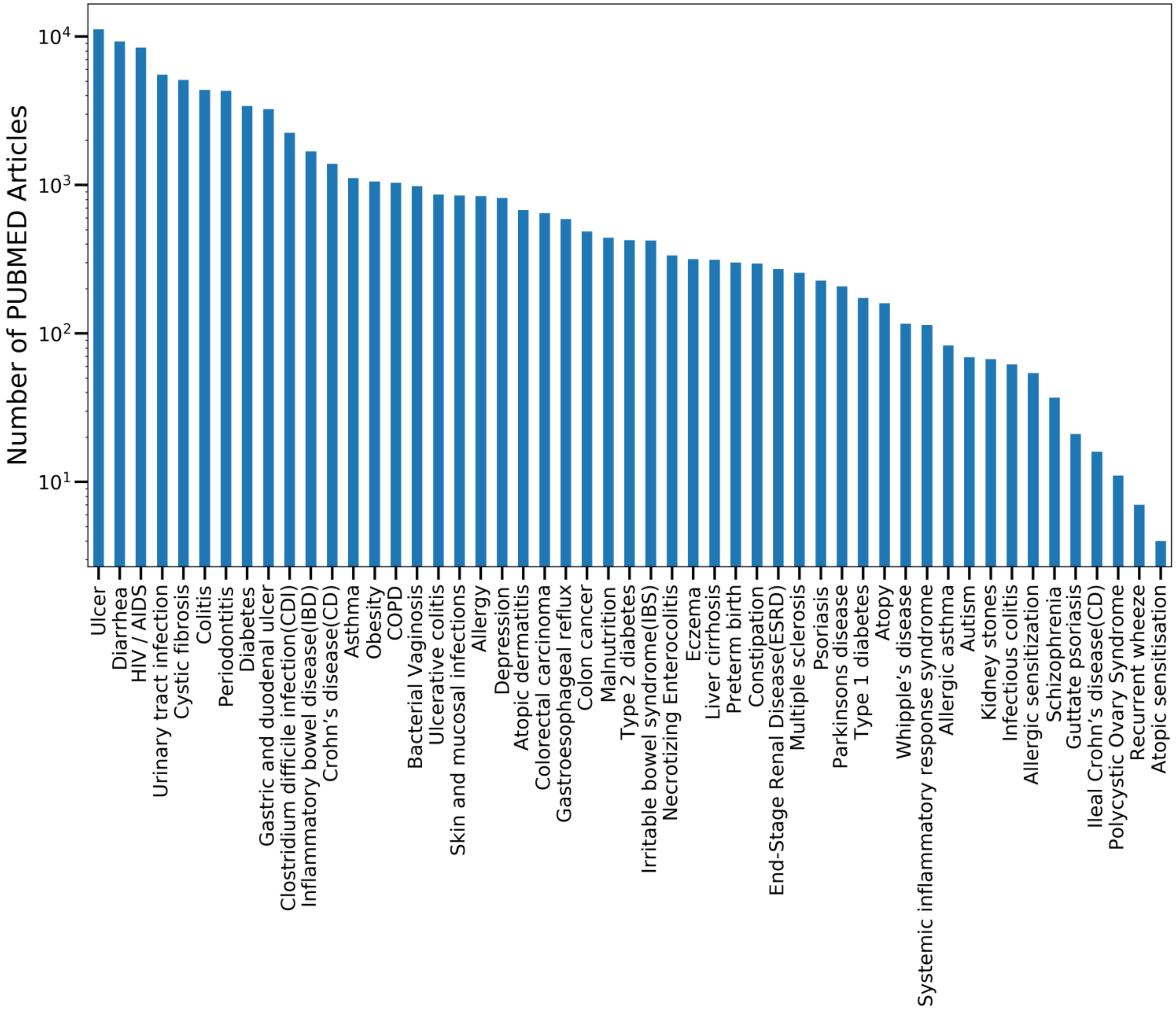

Supplementary Figure 1: Bar plot showing the number of PubMed articles (y axis) which report a disease (x axis). The y axis is shown as a log scale

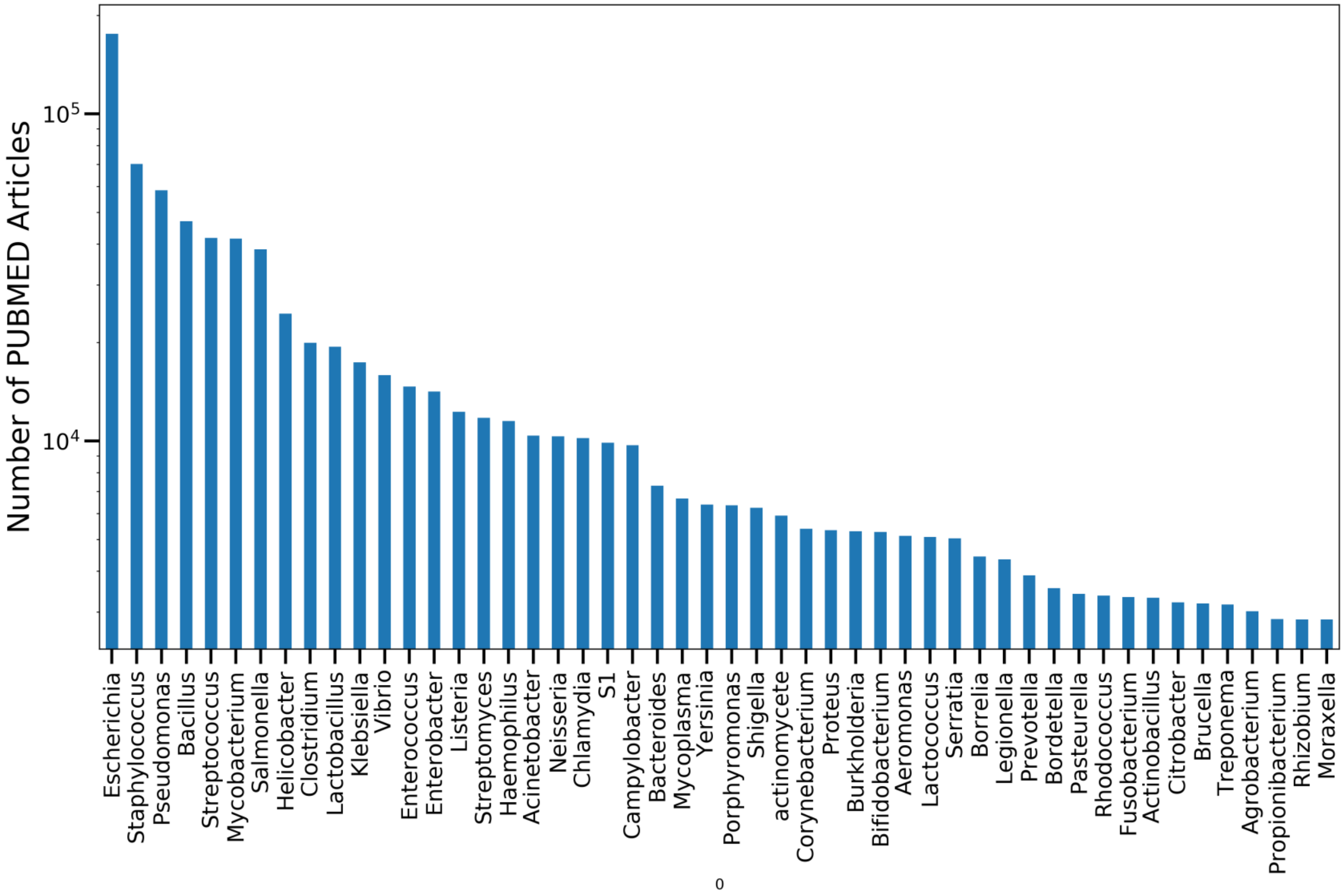

Supplementary Figure 2: Bar plot showing the number of PubMed articles (y axis) which report a taxa (x axis). The top 50 genera are shown on the x axis. The y axis is shown in log scale

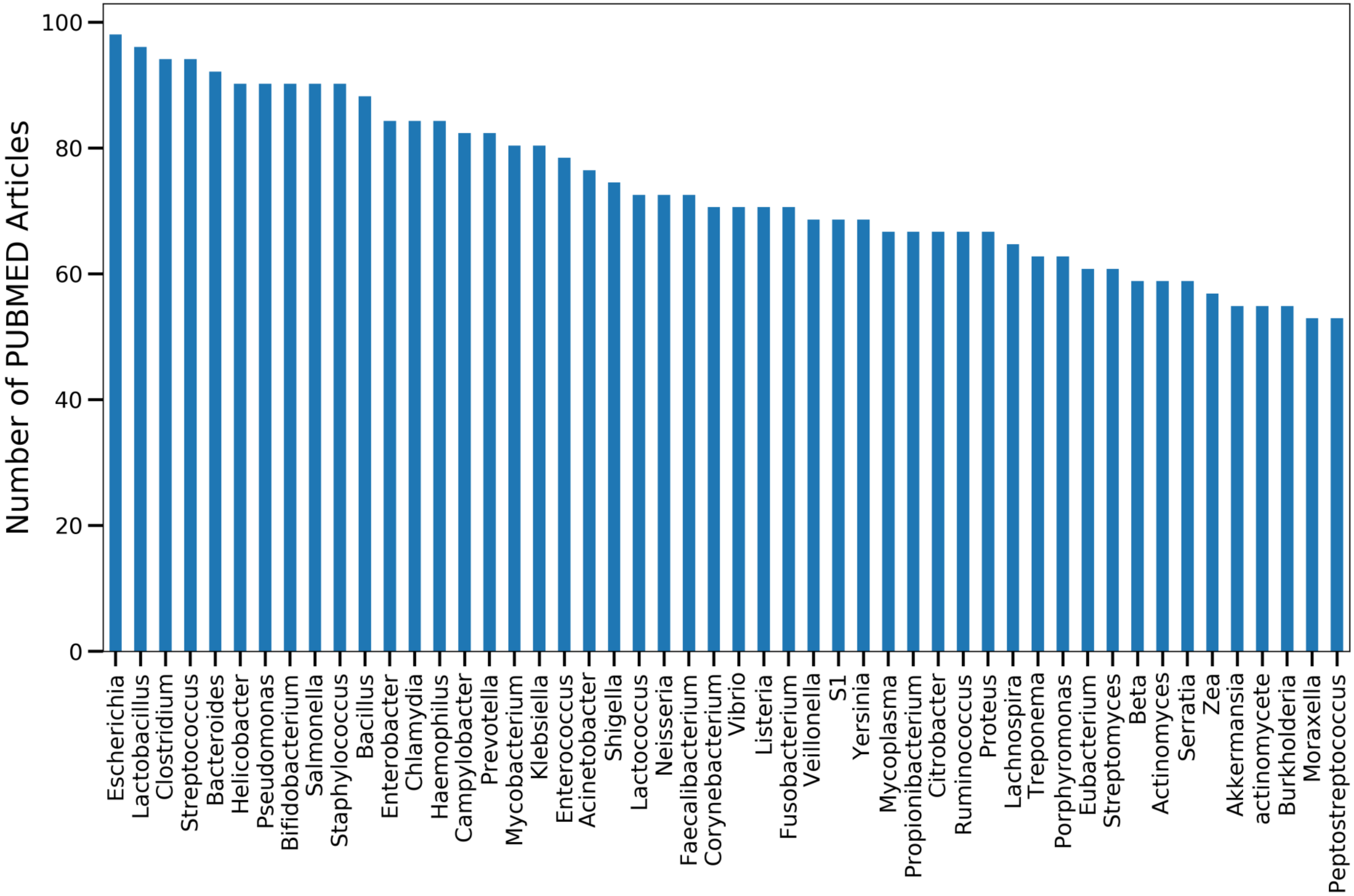

Supplementary Figure 3: Bar plot showing the percentage of diseases (y axis) which are reported to be linked to a taxa (x axis). The top 50 genera are shown on the x axis.

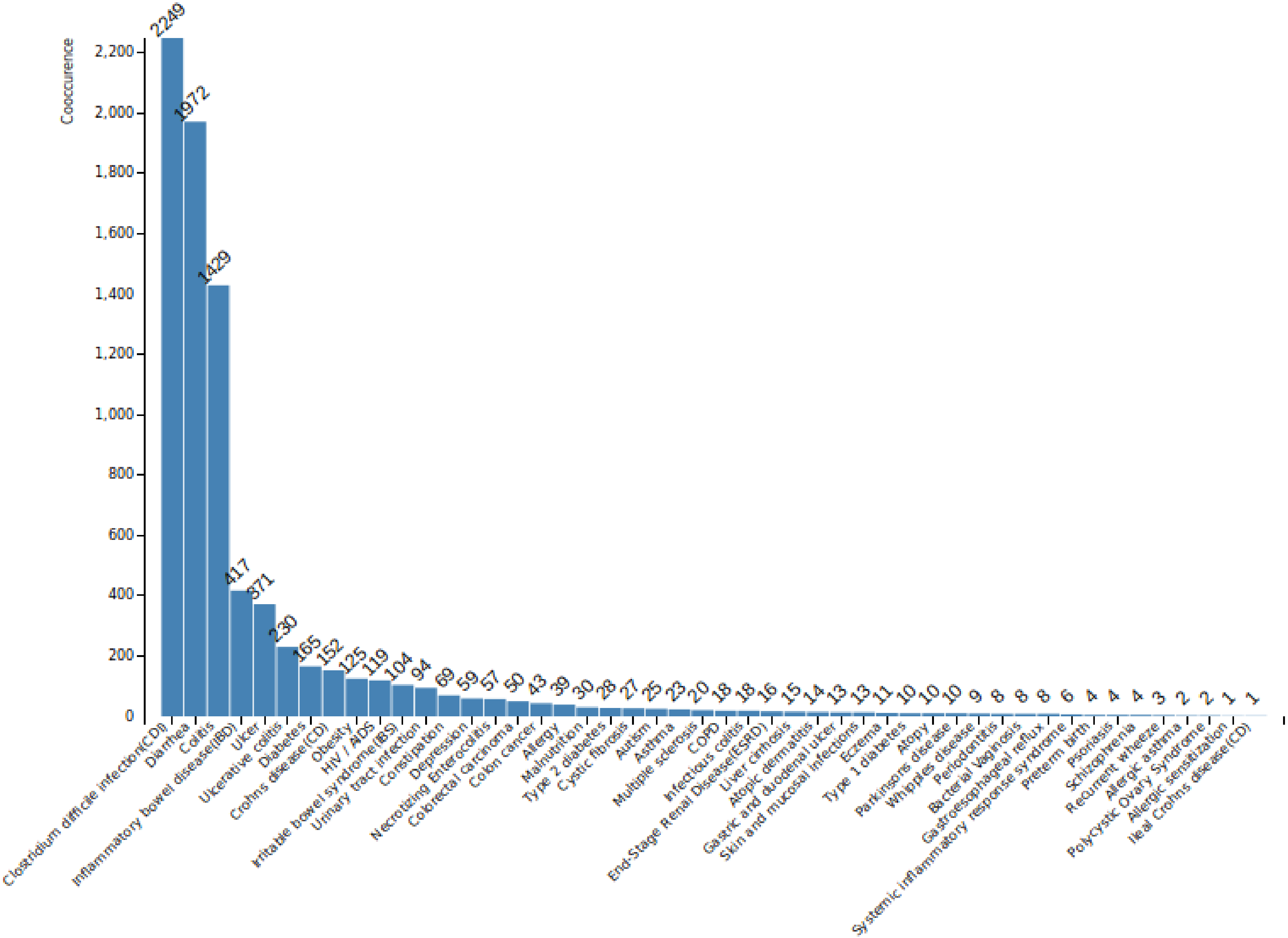

Supplementary Figure 4: Visualizing associated diseases for genera *Clostridium*

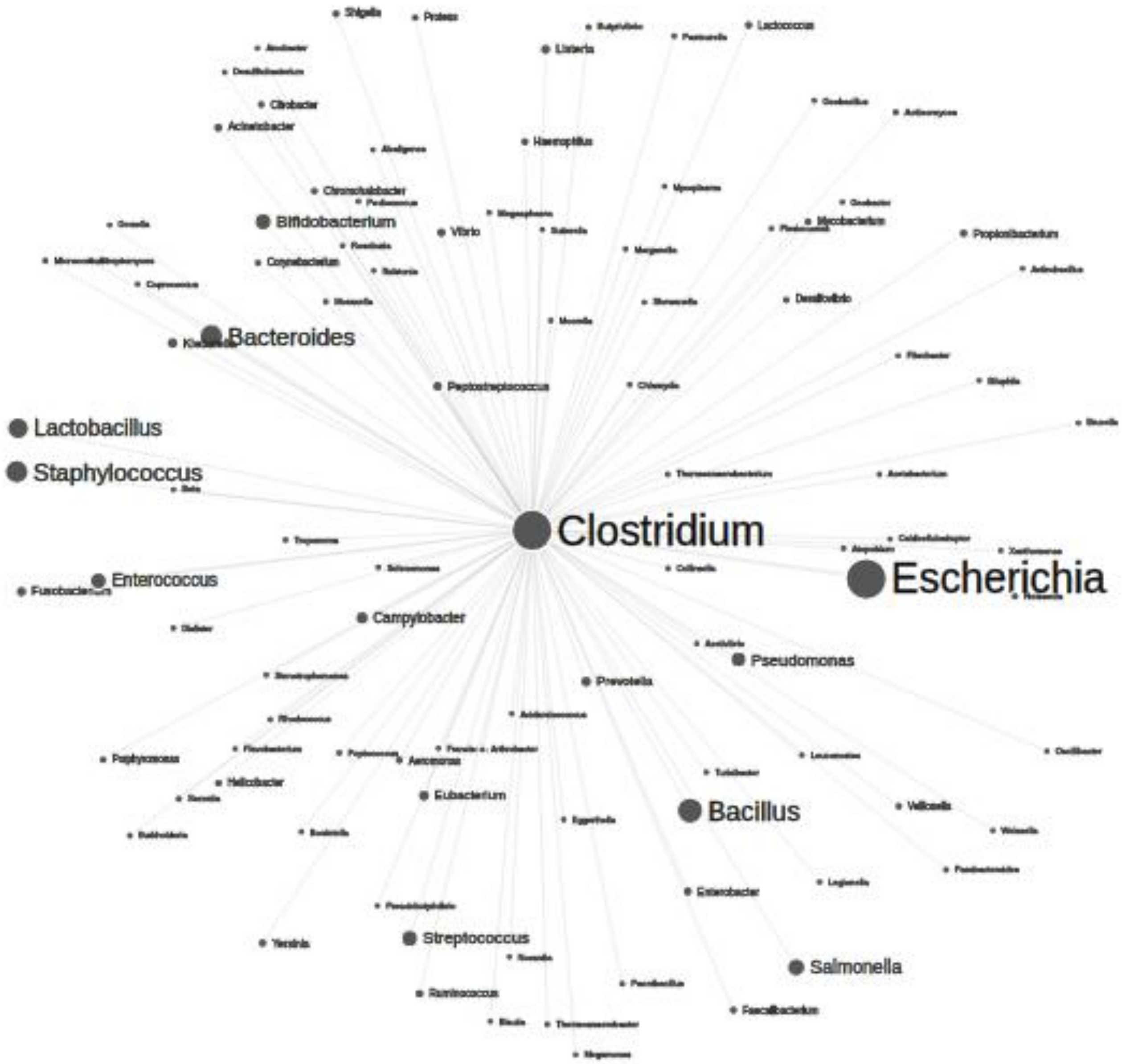

Supplementary Figure 5: Visualizing other genera closely associated with *Clostridium.* The node and label size are mapped to the number of evidences found for the association.

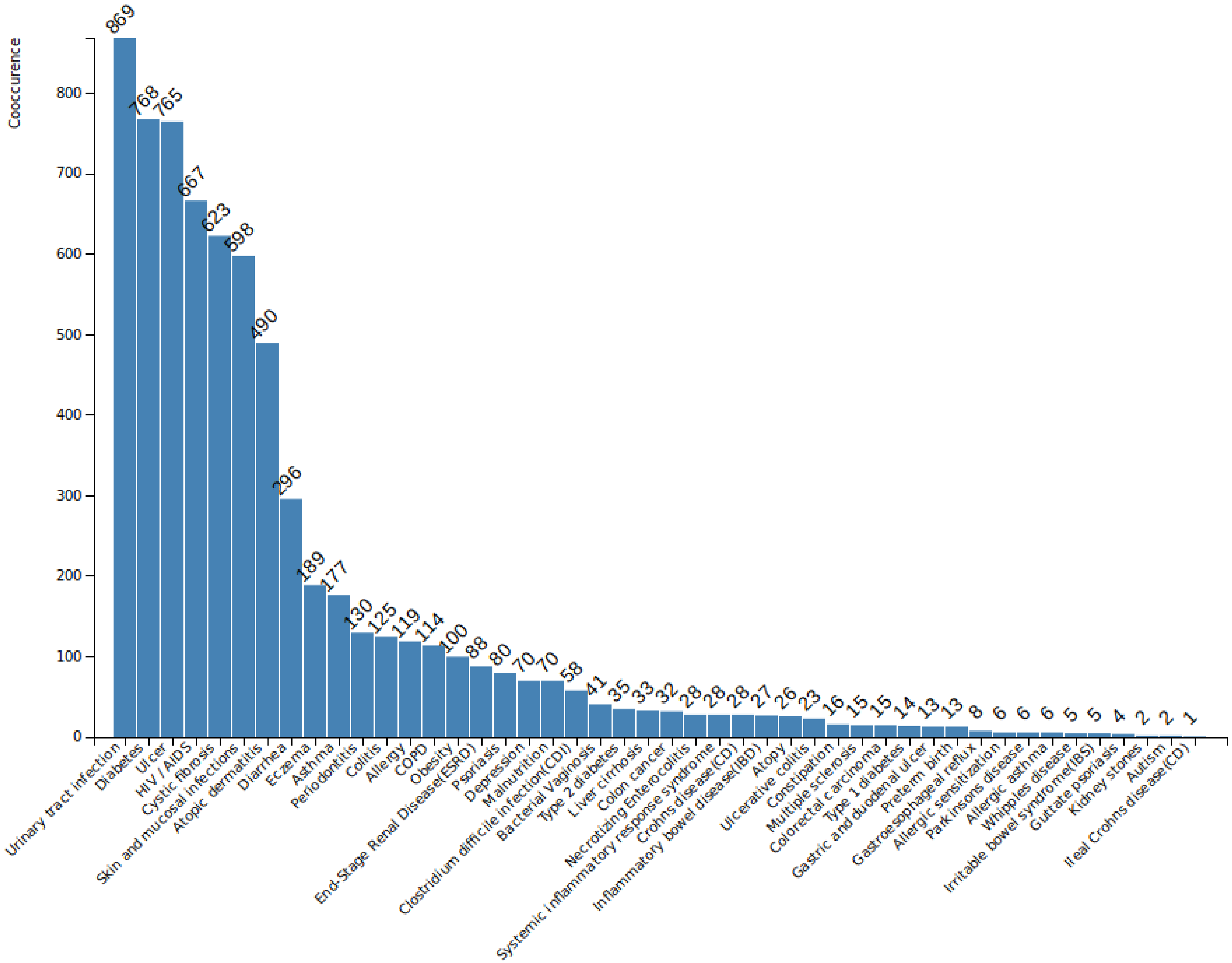

Supplementary Figure 6: Visualizing associated diseases for genera *Staphylococcus*

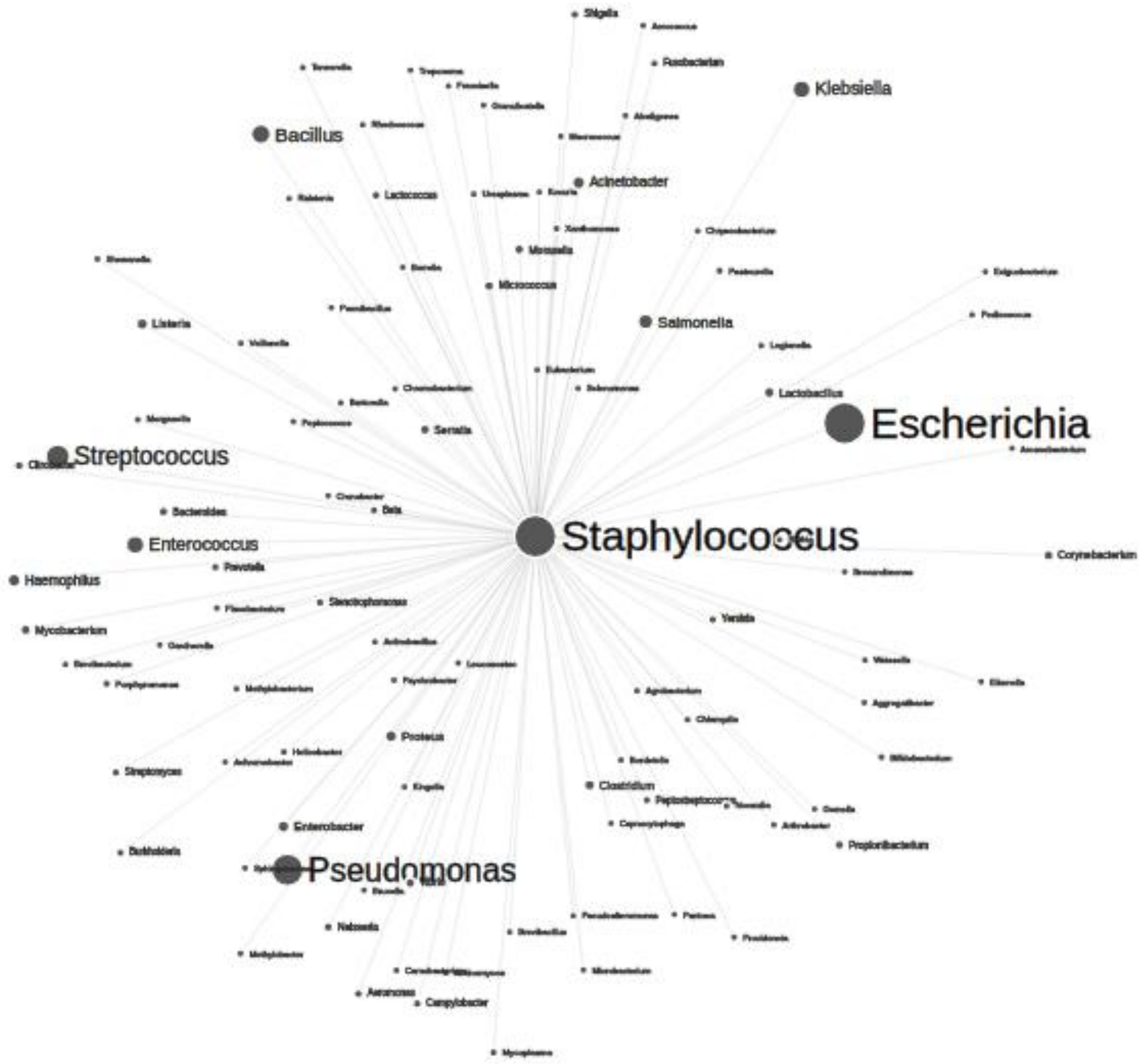

Supplementary Figure 7 : Visualizing other genera closely associated with *Staphylococcus.* The node and label size are mapped to the number of evidences found for the association.

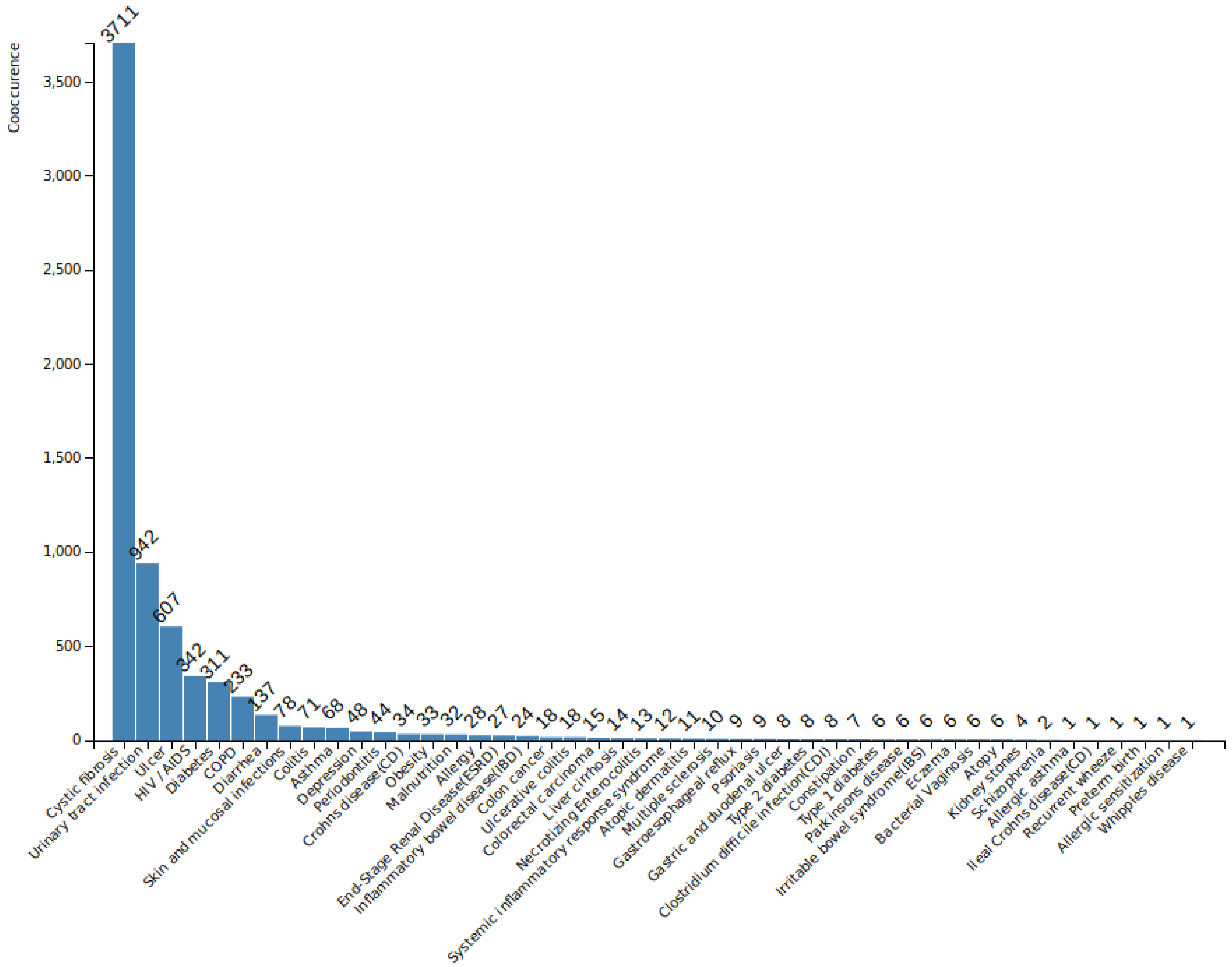

Supplementary Figure 8 : Visualizing associated diseases for genera *Pseudomonas*

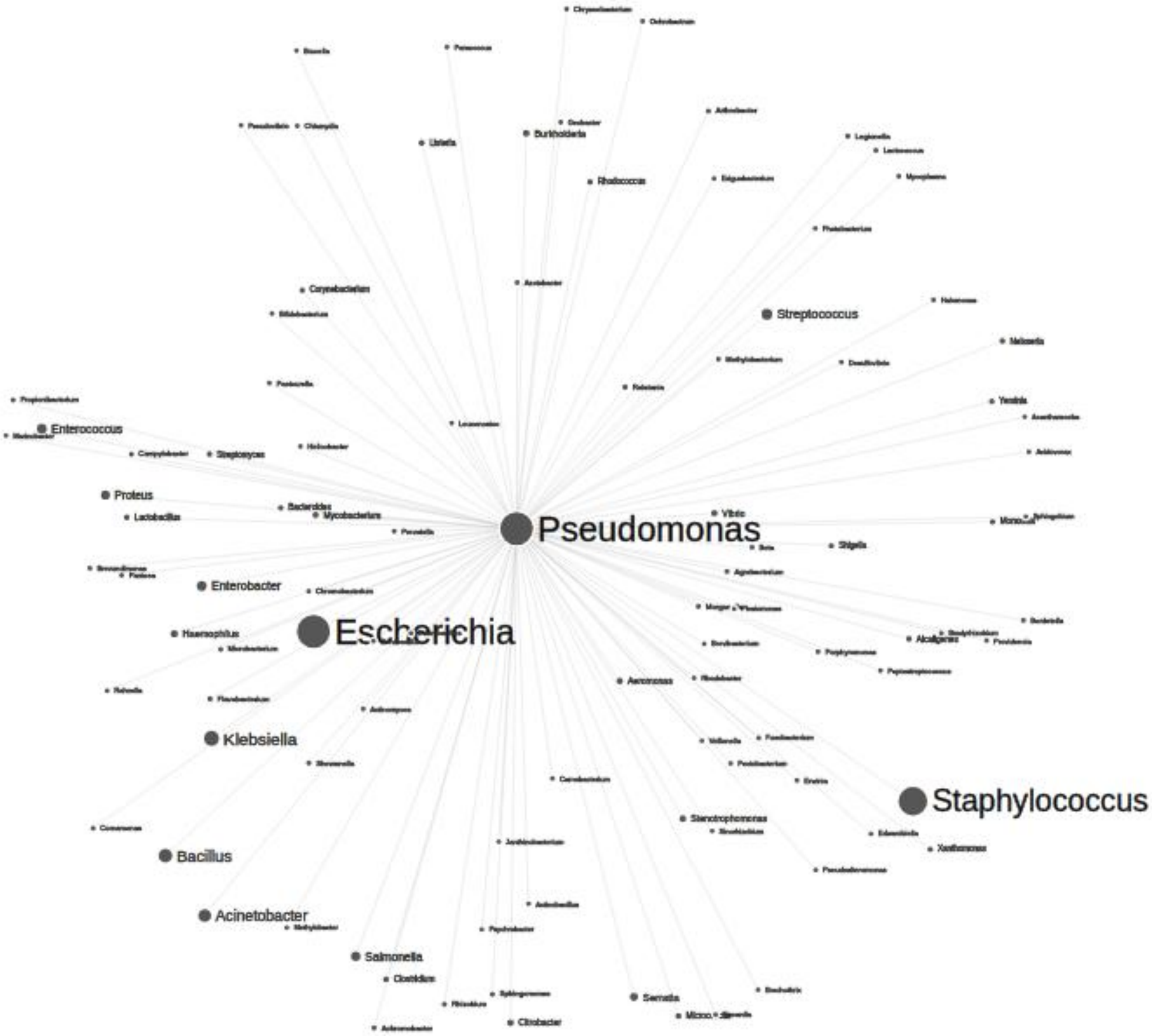

Supplementary Figure 9 : Visualizing other genera closely associated with *Pseudomonas.* The node and label size are mapped to the number of evidences found for the association.

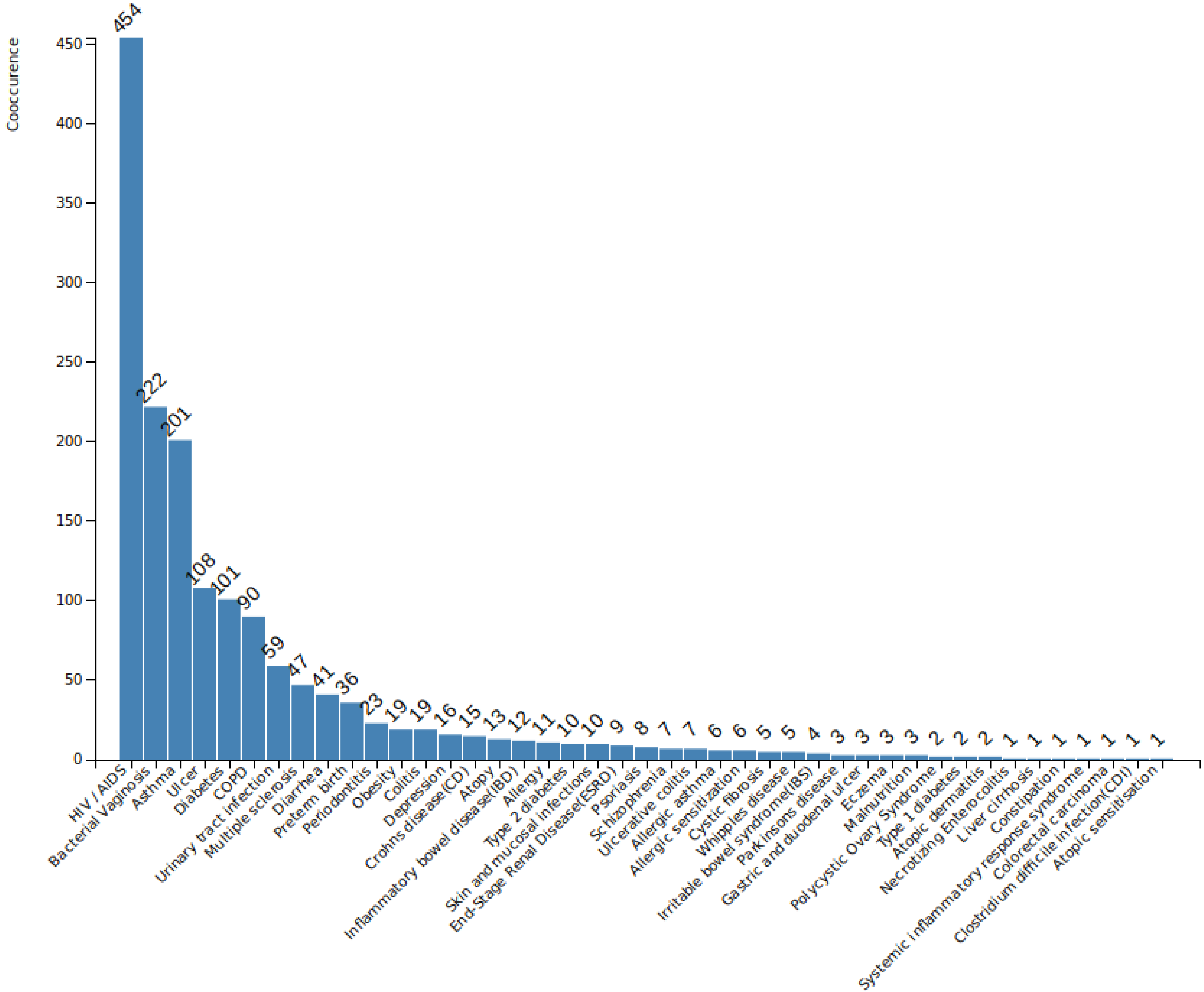

Supplementary Figure 10 : Visualizing associated diseases for genera *Chlamydia*

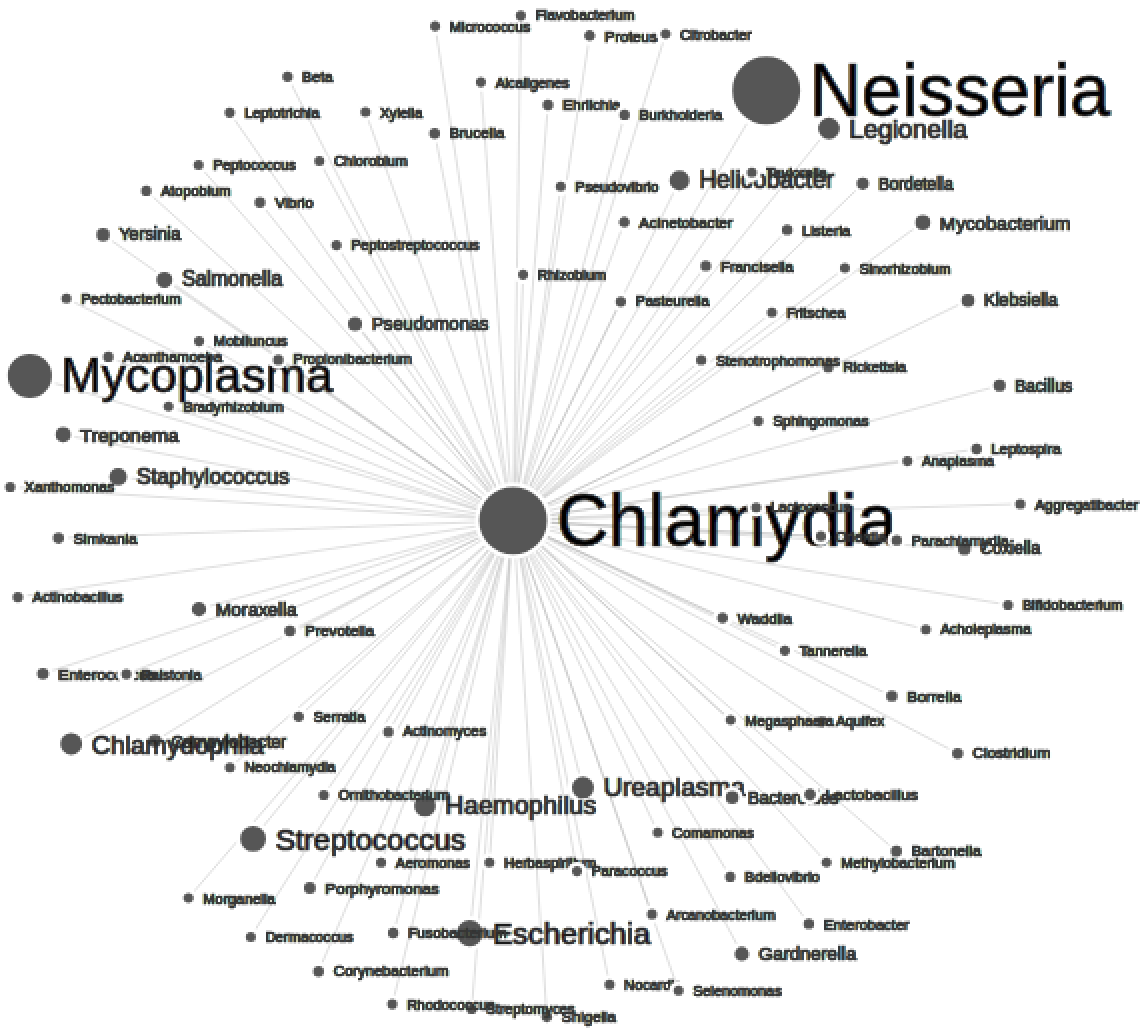

Supplementary Figure 11 : Visualizing other genera closely associated with *Chlamydia.* The node and label size are mapped to the number of evidences found for the association.

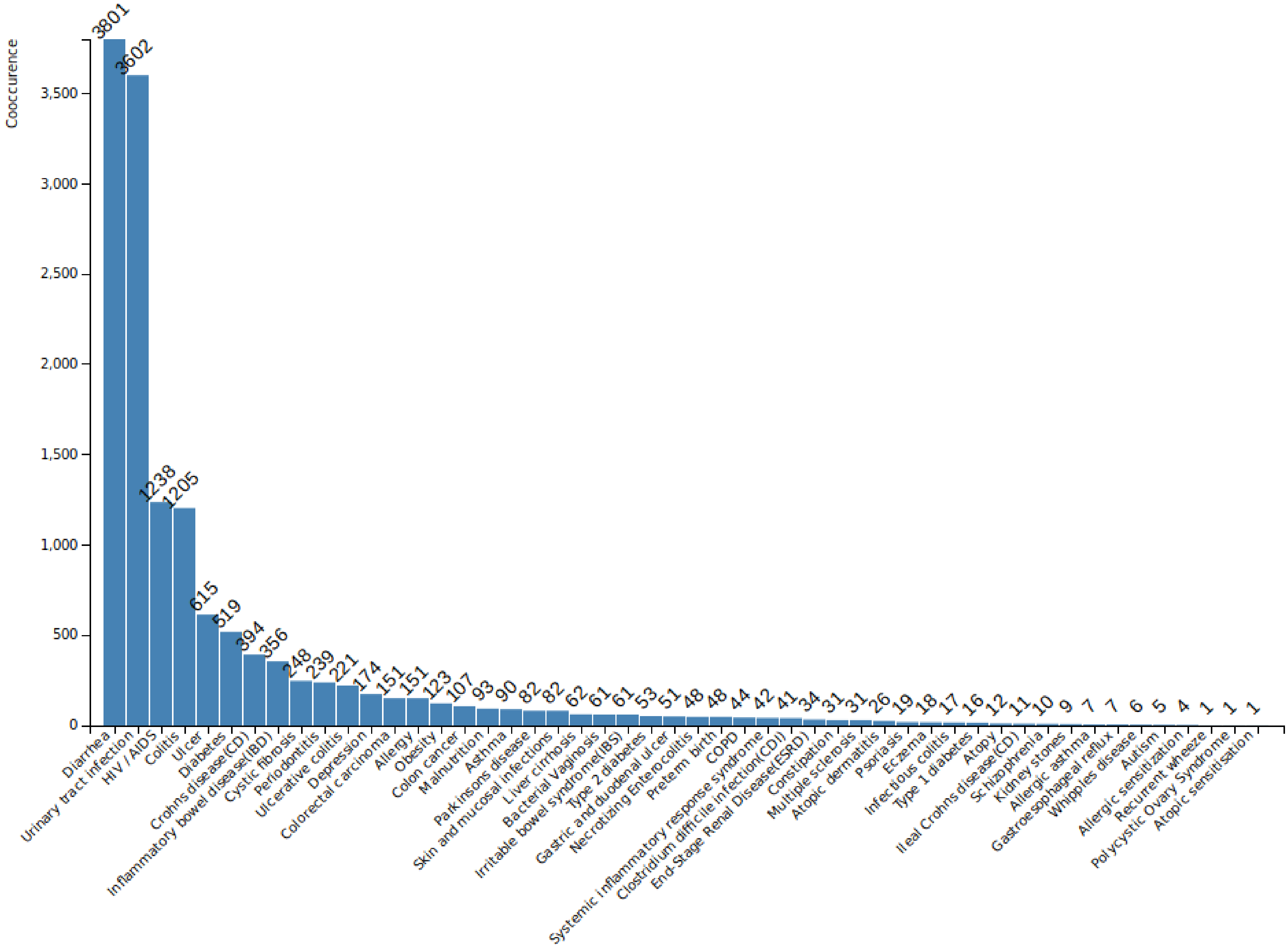

Supplementary Figure 12 : Visualizing associated diseases for genera *Escherichia*

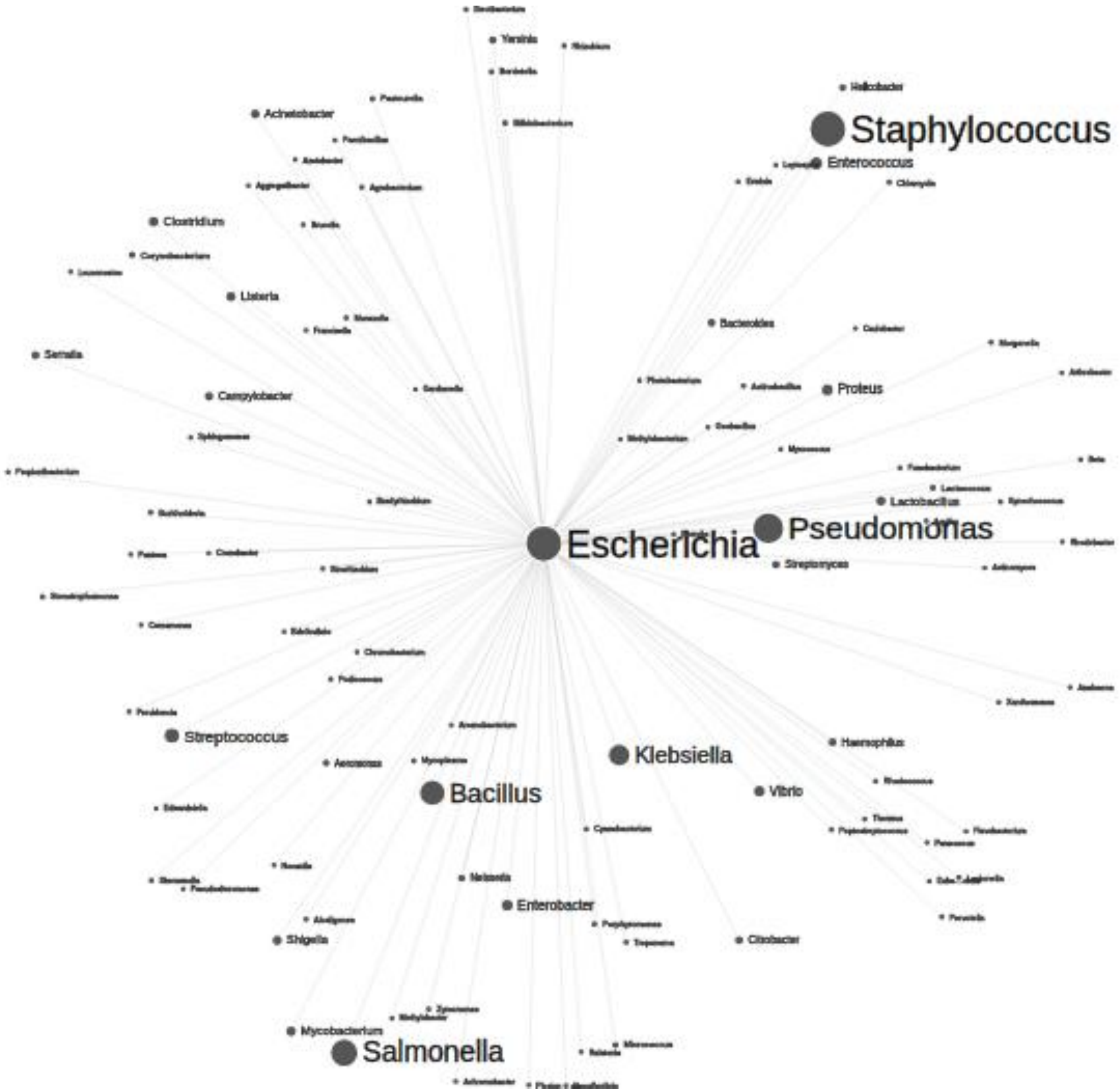

Supplementary Figure 13 : Visualizing other genera closely associated with *Escherichia.* The node and label size are mapped to the number of evidences found for the association.

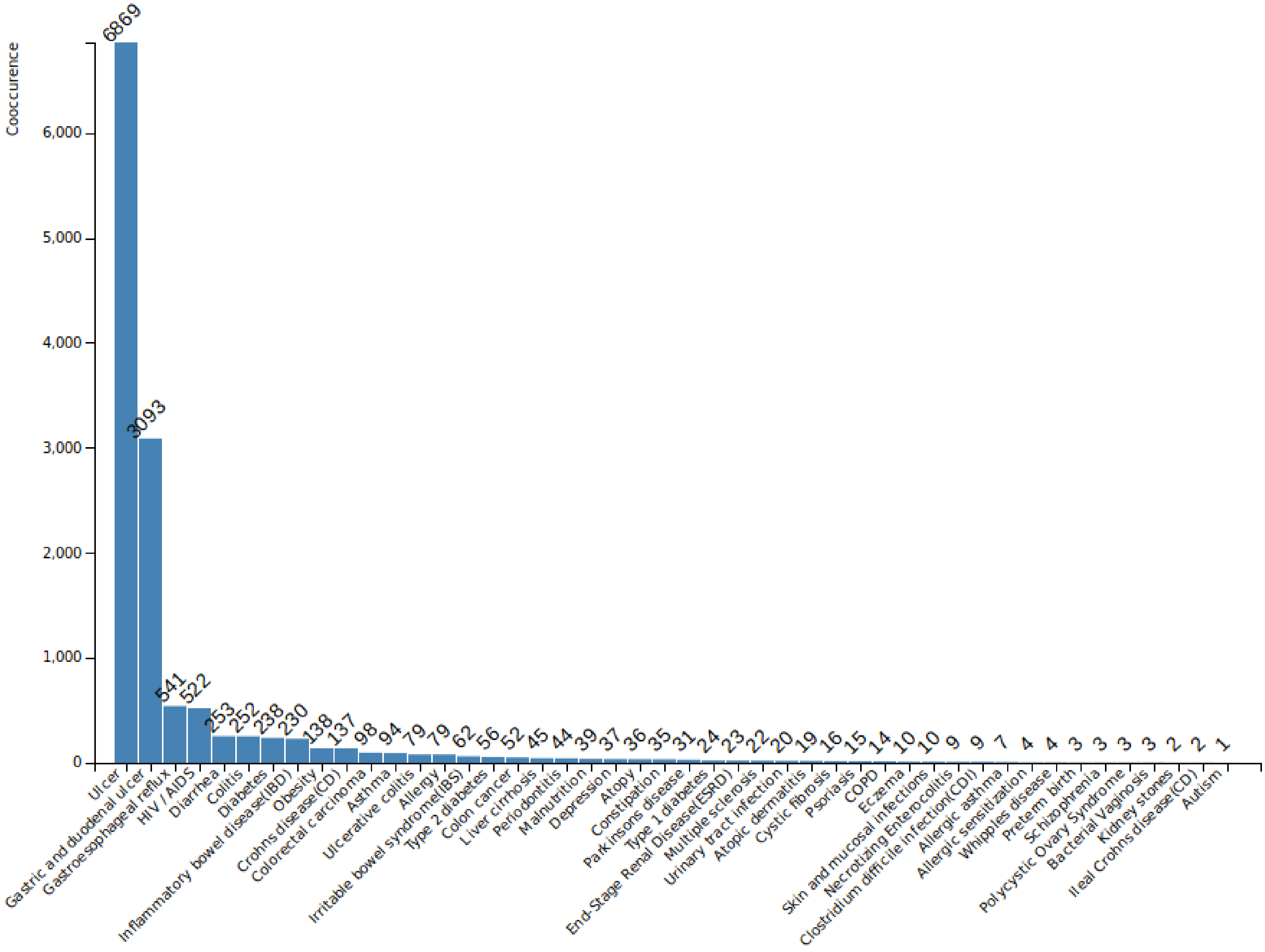

Supplementary Figure 14 : Visualizing associated diseases for genera *Helicobacter*

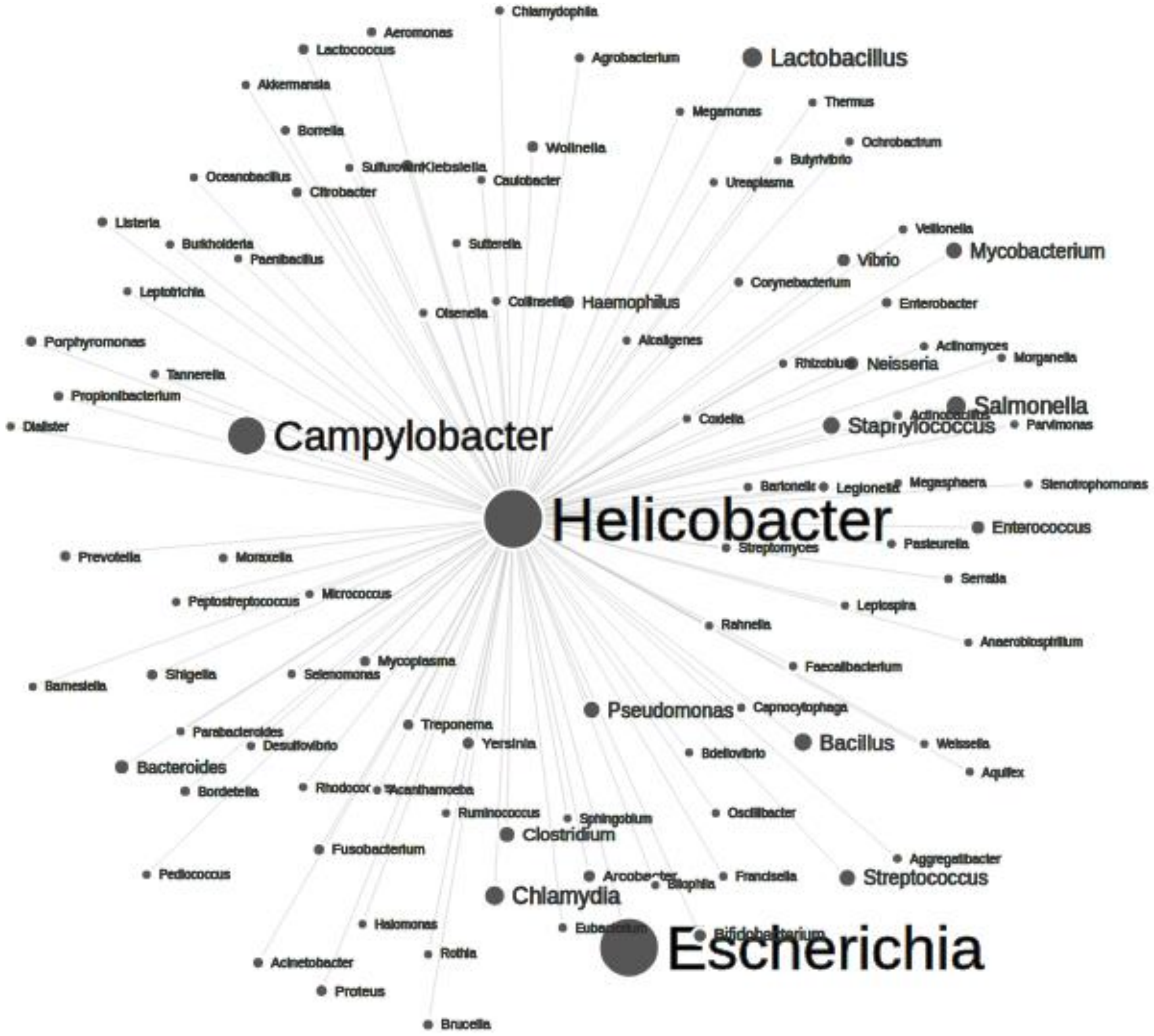

Supplementary Figure 15 : Visualizing other genera closely associated with *Helicobacter.* The node and label size are mapped to the number of evidences found for the association.

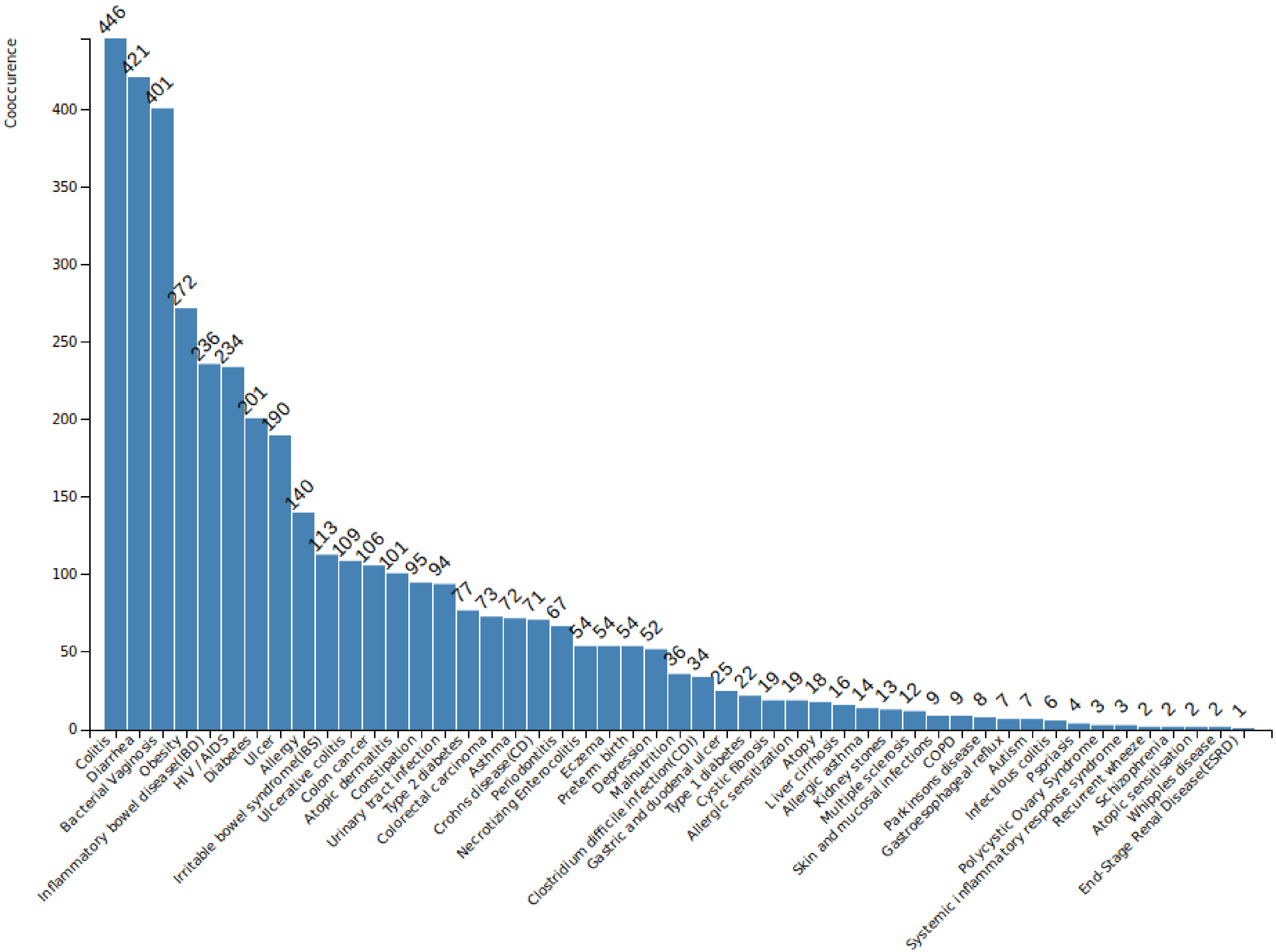

Supplementary Figure 16 : Visualizing associated diseases for genera *Lactobacillus*

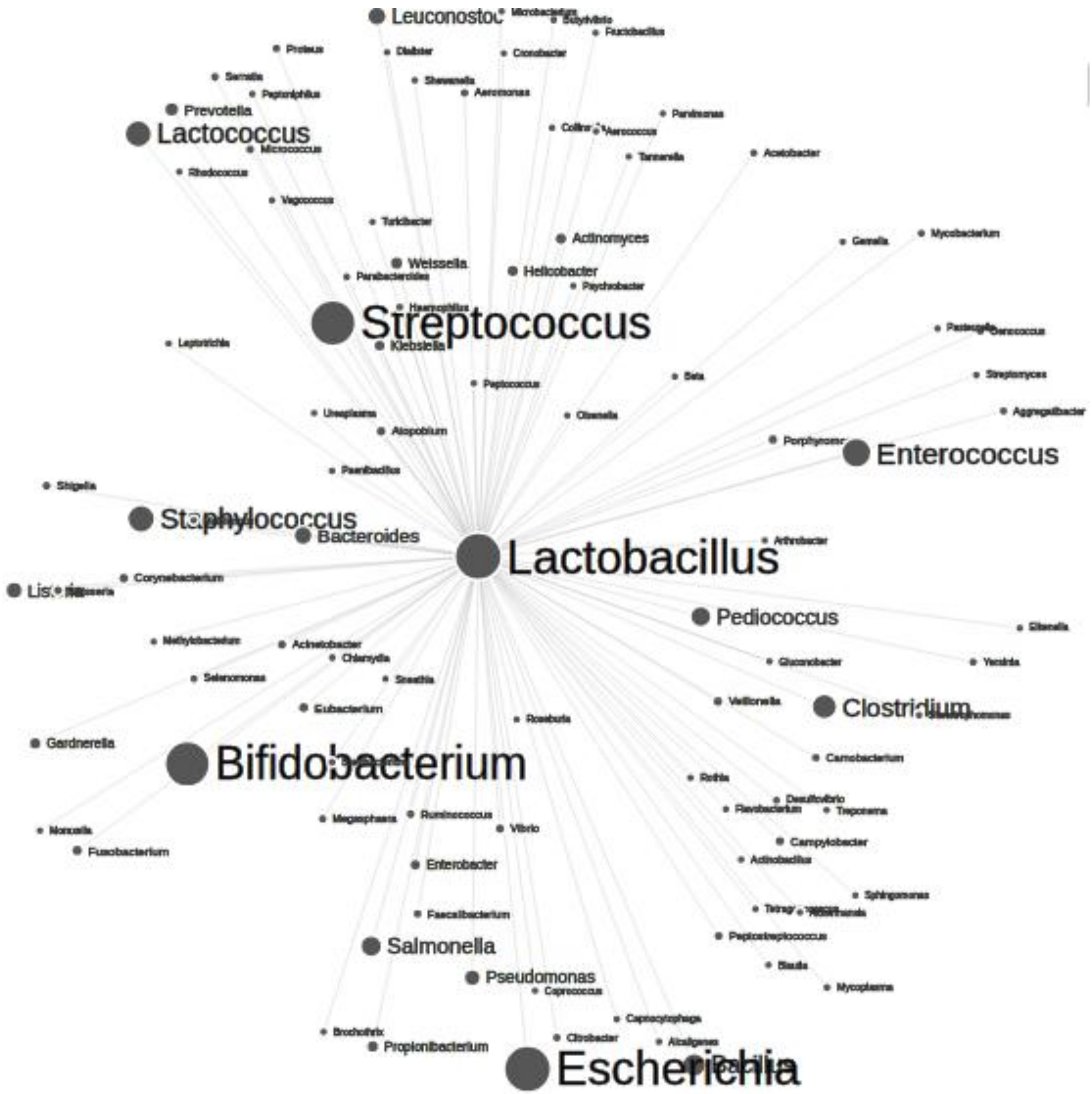

Supplementary Figure 17 : Visualizing other genera closely associated with *Lactobacillus.* The node and label size are mapped to the number of evidences found for the association.

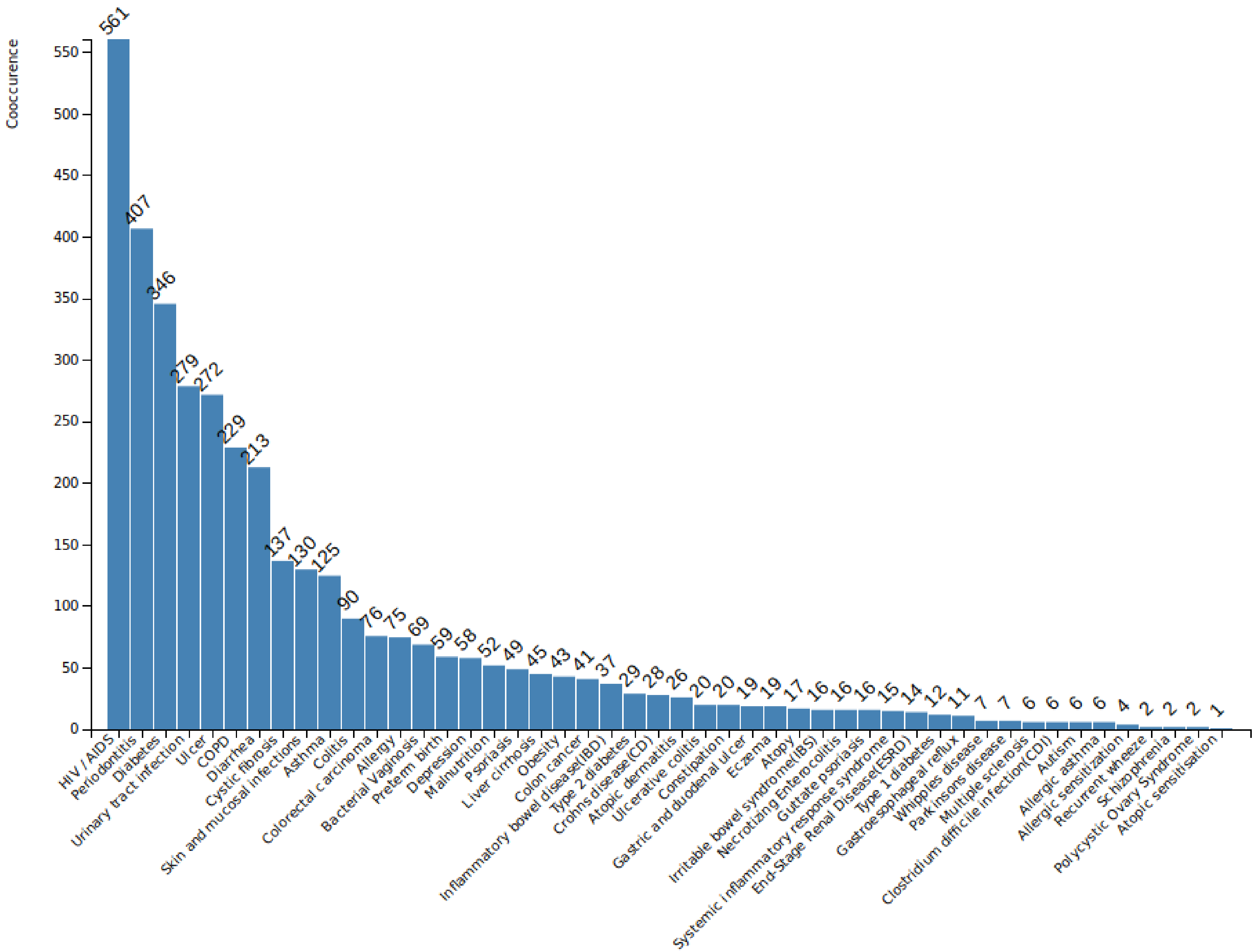

Supplementary Figure 18 : Visualizing associated diseases for genera *Streptococcus*

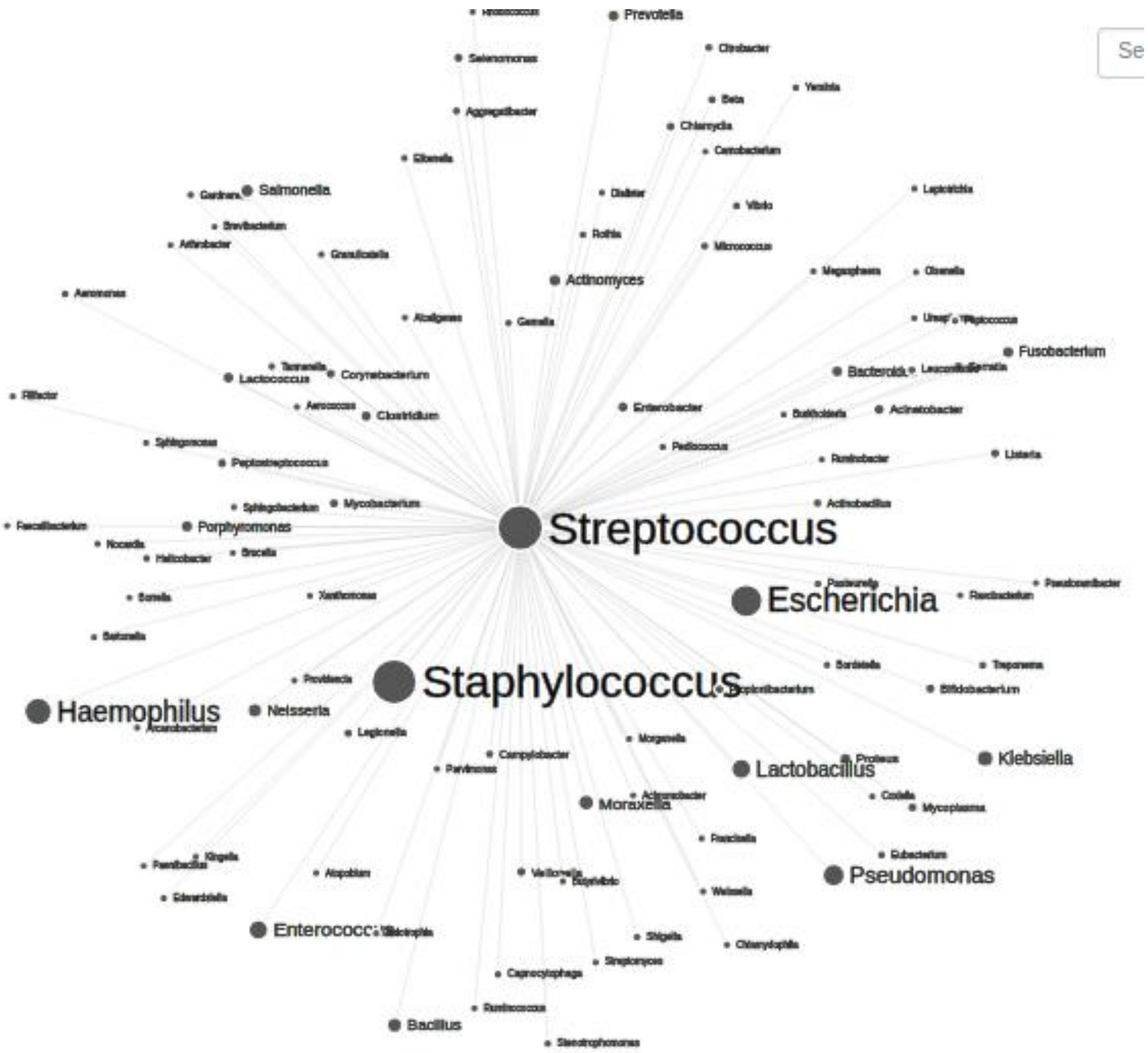

Supplementary Figure 19 : Visualizing other genera closely associated with *Streptococcus.* The node and label size are mapped to the number of evidences found for the association.

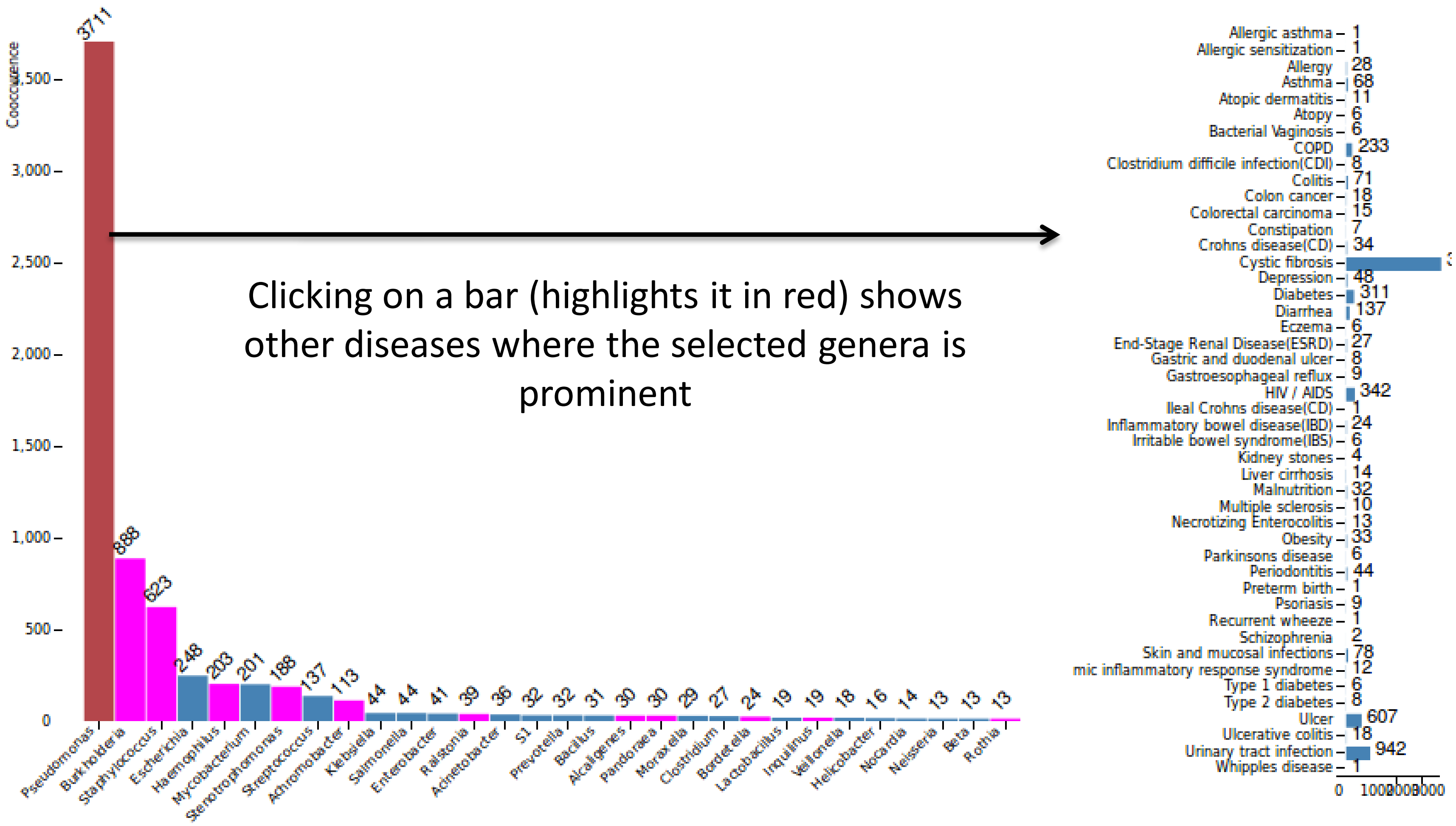

N.B: Pink bars represent the taxa identified to be associated significantly with the disease

Supplementary Figure 20 : Visualizing top genera associated with disease Cystic Fibrosis

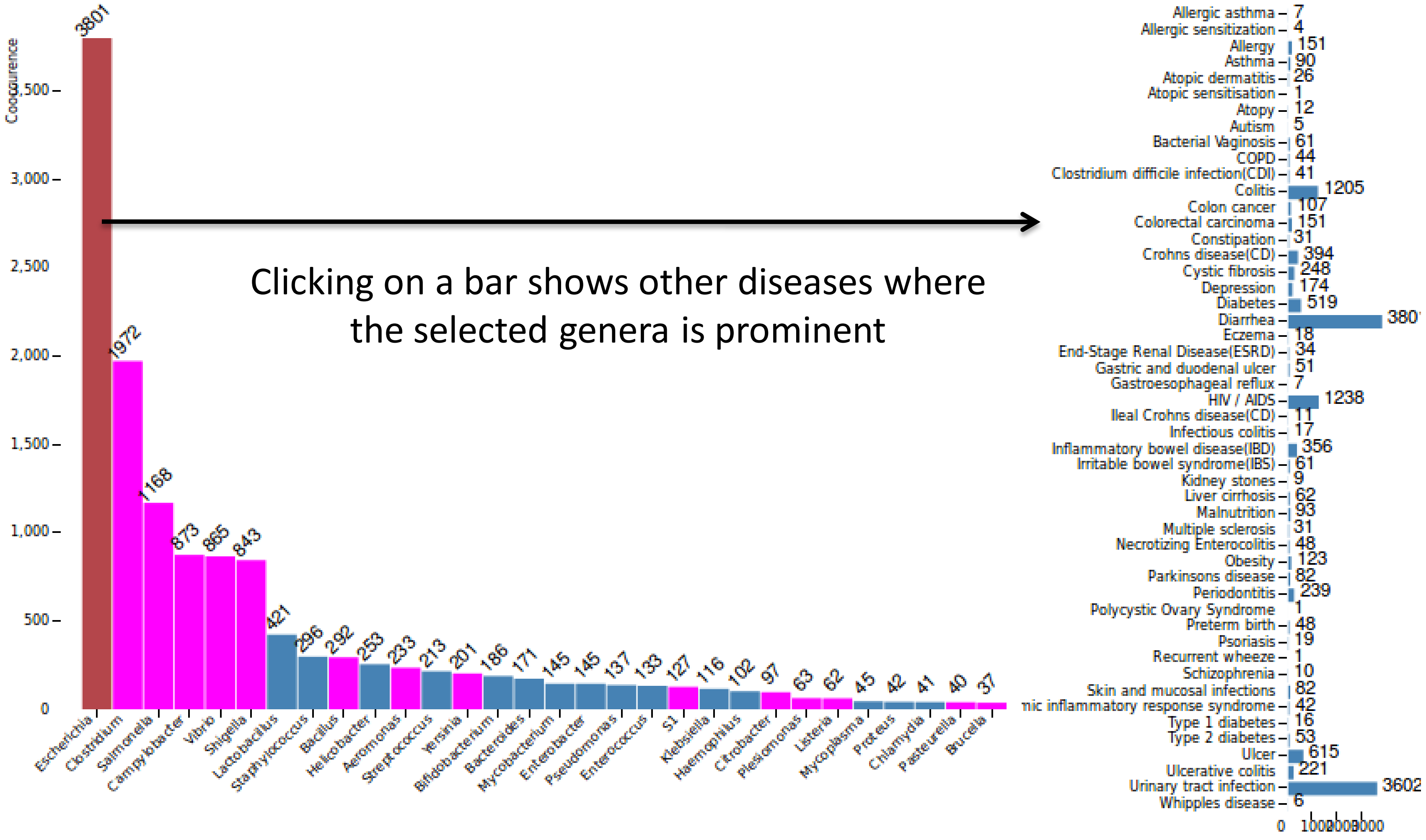

N.B: Pink bars represent the taxa identified to be associated significantly with the disease

Supplementary Figure 21 : Visualizing top genera associated with disease Diarrhea

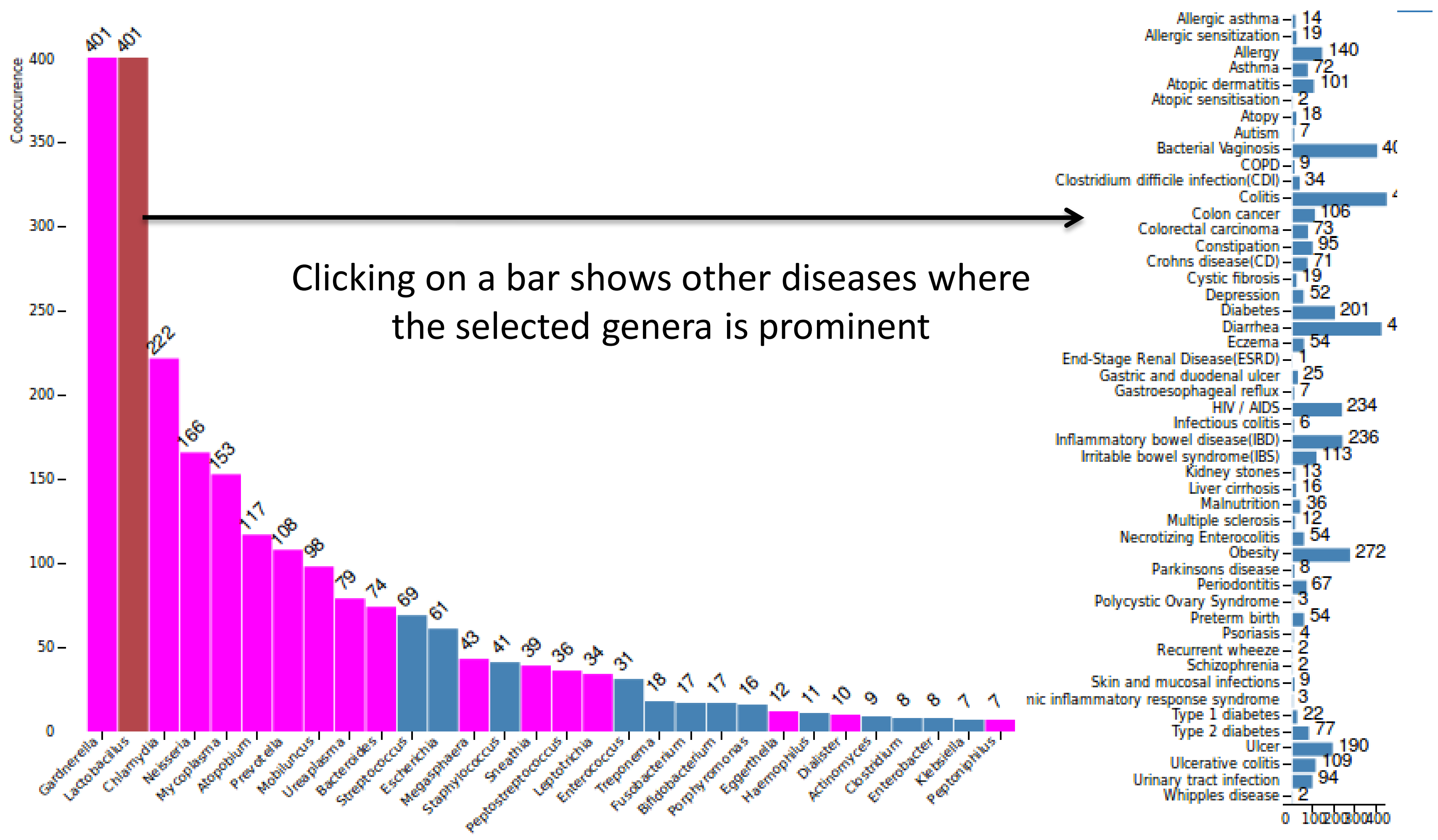

N.B: Pink bars represent the taxa identified to be associated significantly with the disease

Supplementary Figure 22 : Visualizing top genera associated with disease Bacterial vaginosis

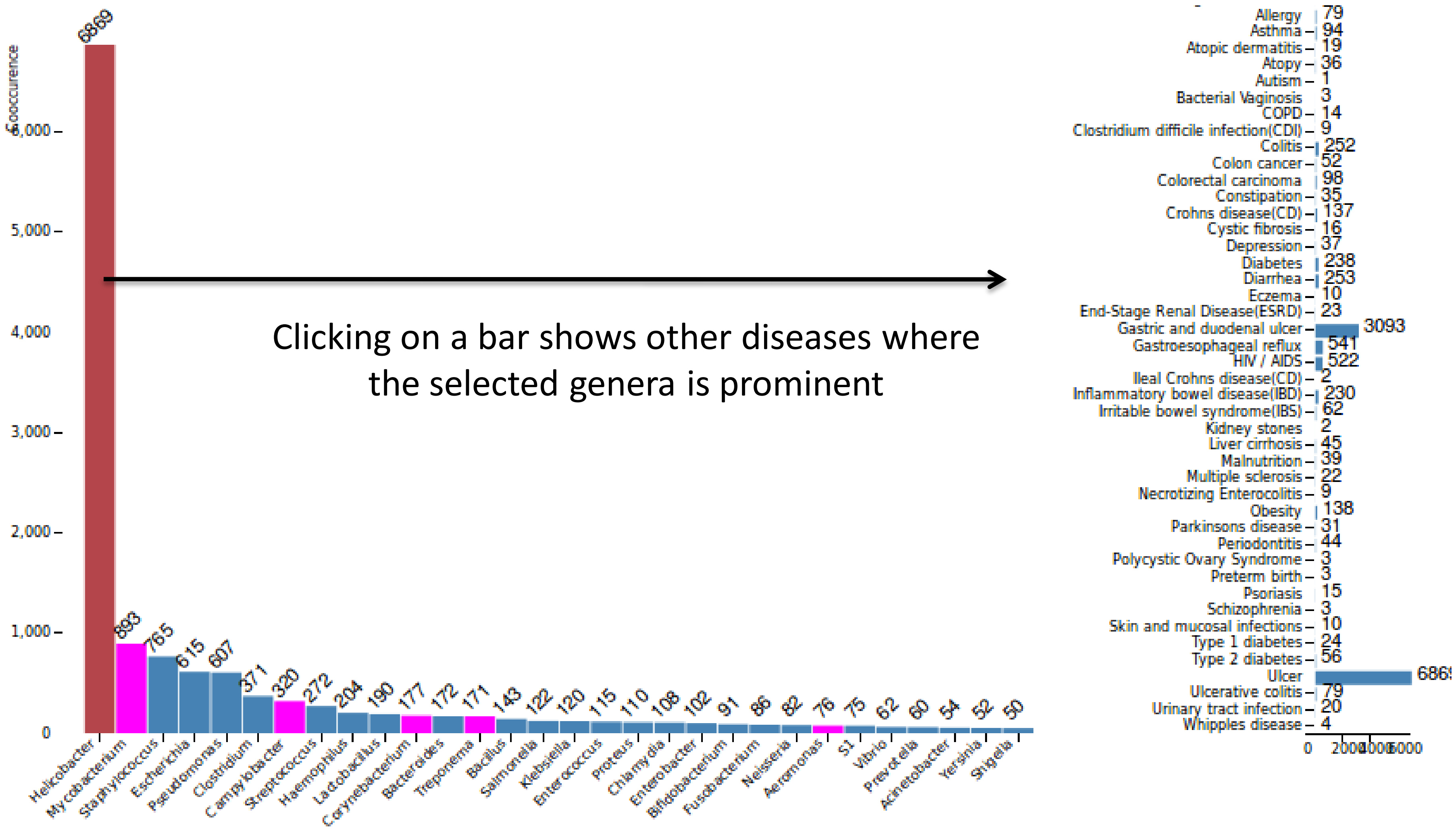

N.B: Pink bars represent the taxa identified to be associated significantly with the disease

Supplementary Figure 23 : Visualizing top genera associated with disease Ulcer

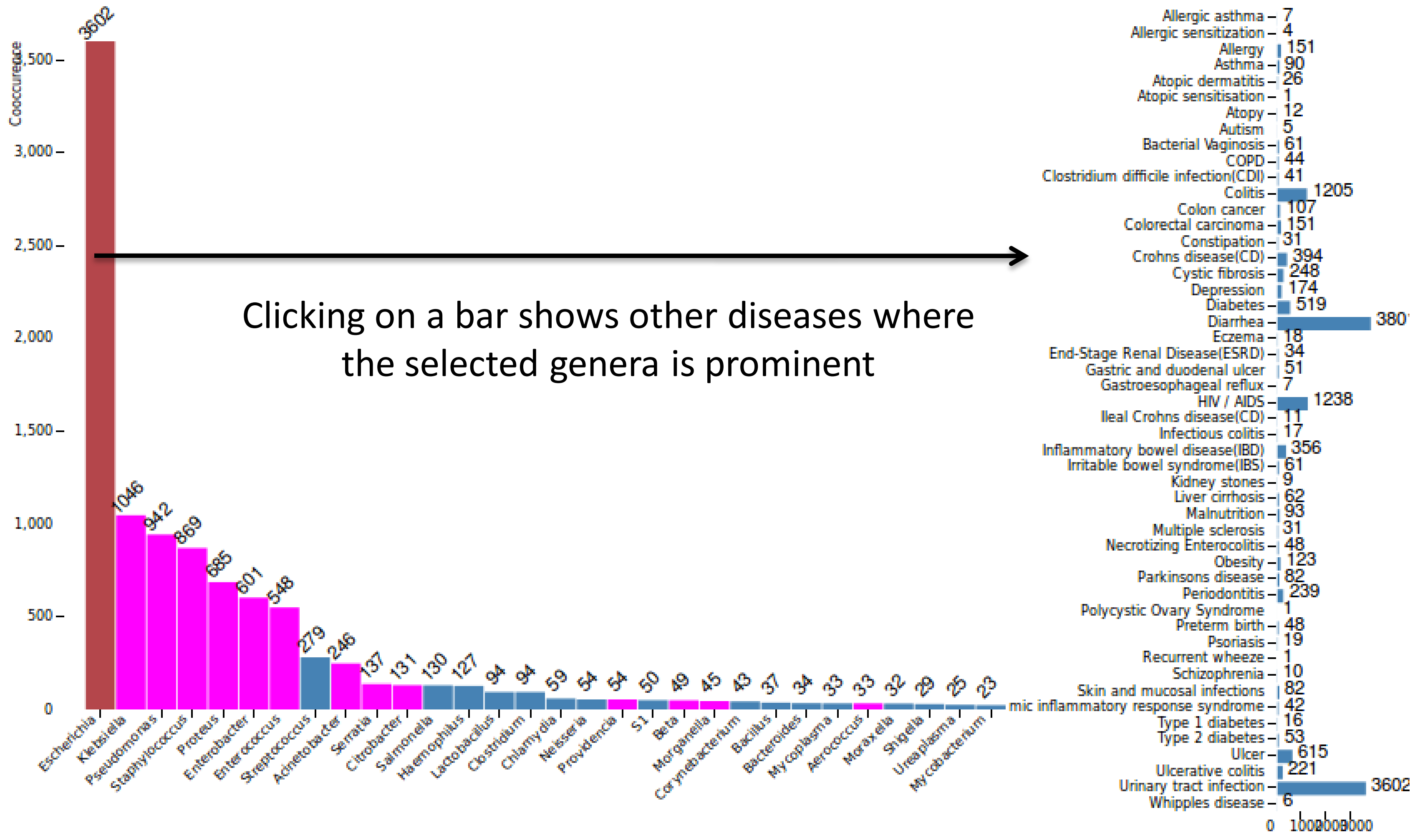

N.B: Pink bars represent the taxa identified to be associated significantly with the disease

Supplementary Figure 24 : Visualizing top genera associated with disease UTI

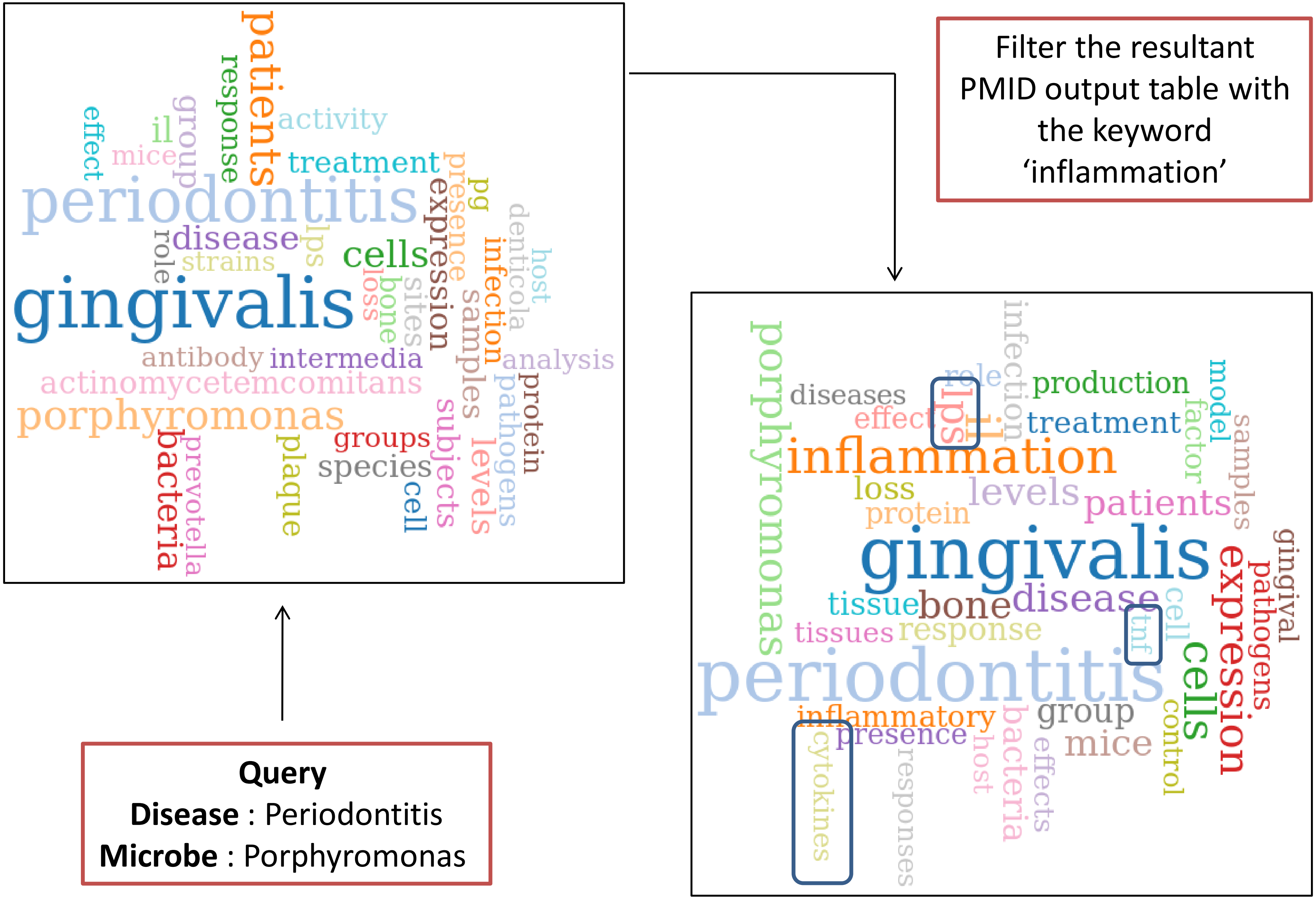

Supplementary Figure 25 : Utility of 'word cloud' feature

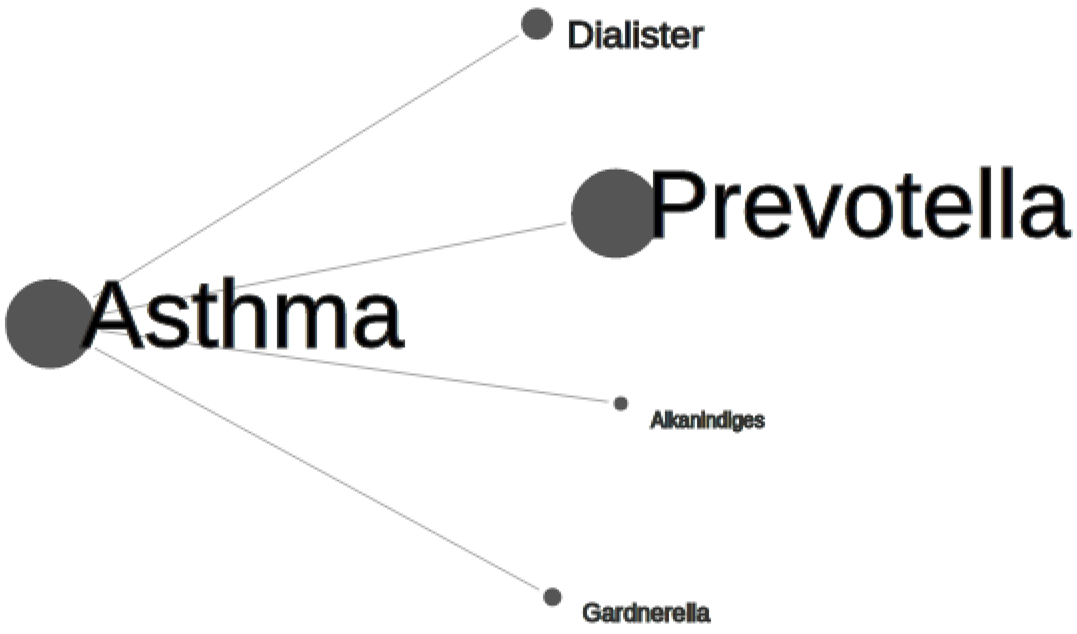

Evidence Statistics

Cooccurence Statistics:	
**Genus/Disease**	**Number of Abstracts**
Asthma AND Prevotella	18
ONLY Prevotella	3859
ONLY Asthma	1096
NONE	632455

P Values		
**Statistical Test for Significance of Cooccurence**	**Uncorrected P value**	**Bonferroni Corrected P value**
One Sided Fisher Exact Test	<0.001	<0.001
Two Sided Fisher Exact Test	<0.001	<0.001
Chi Square Test	<0.0001	<0.0001

Supplementary Figure 26 : Evidence of selected Asthma associated genera. The node and label size are mapped to the number of evidences found for the association.

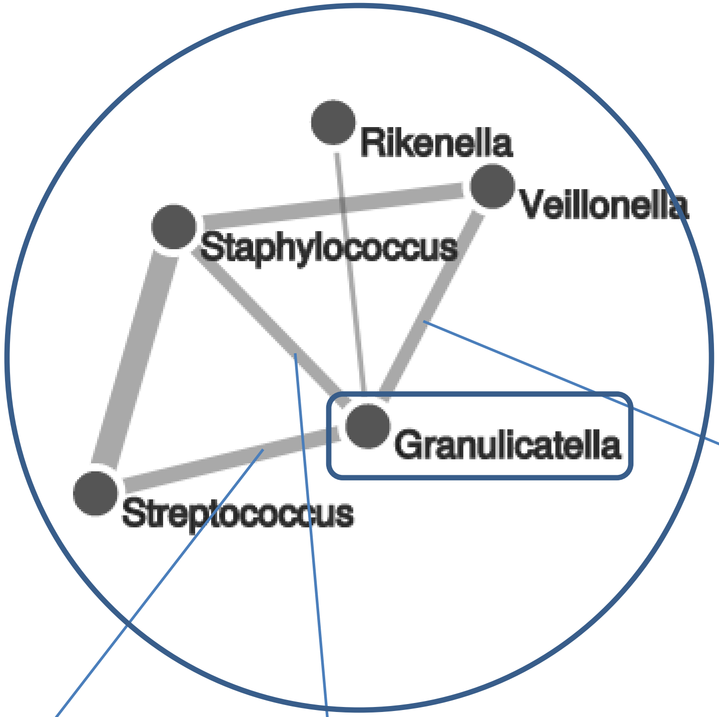

Genus	In Allergic asthma	In All Diseases	In All Abstracts
Granulicatella	0	35	165
Streptococcus	6	3170	41688
Both	0	18	80

Genus	In Allergic asthma	In All Diseases	In All Abstracts
Granulicatella	0	35	165
Staphylococcus	6	5801	70266
Both	0	8	23

Genus	In Allergic asthma	In All Diseases	In All Abstracts
Granulicatella	0	35	165
Veillonella	0	228	915
Both	0	14	35

Supplementary Figure 27 : Evidence indices for *Granulicatella*

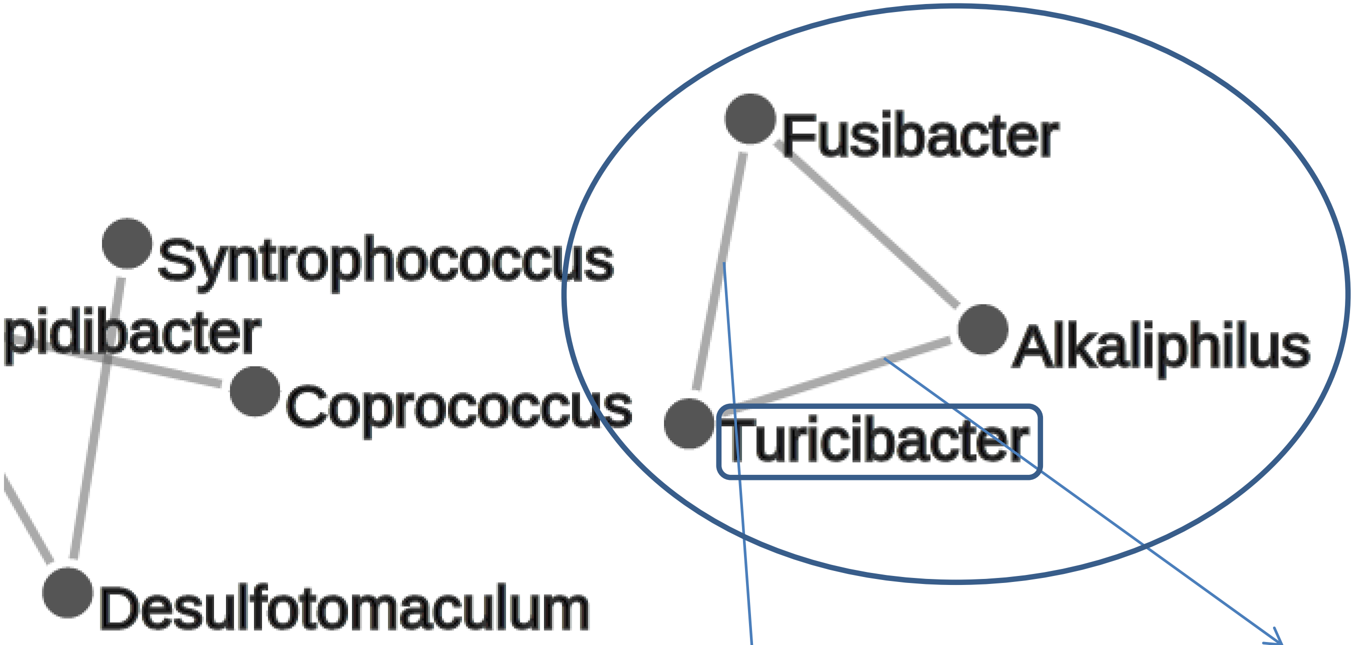

Genus	In Allergic asthma	In All Diseases	In All Abstracts
Fusibacter	0	1	16
Turicibacter	0	28	83
Both	0	0	0

Genus	In Allergic asthma	In All Diseases	In All Abstracts
Alkaliphilus	0	0	56
Turicibacter	0	28	83
Both	0	0	0

Supplementary Figure 28 : Evidence indices for *Turicibacter*
