# Supplementary material for: “EviMass”: A Literature Evidence-Based Miner for Human Microbial Associations
Source: Front Genet. 2019 Sep 13;10:849. doi: 10.3389/fgene.2019.00849 (PMC6763948; doi:10.3389/fgene.2019.00849)
Supplement: Supplementary file 2 [file DataSheet_2.pdf]

‘EviMass’ user manual

Module 1 **Module 2** **Module 3**

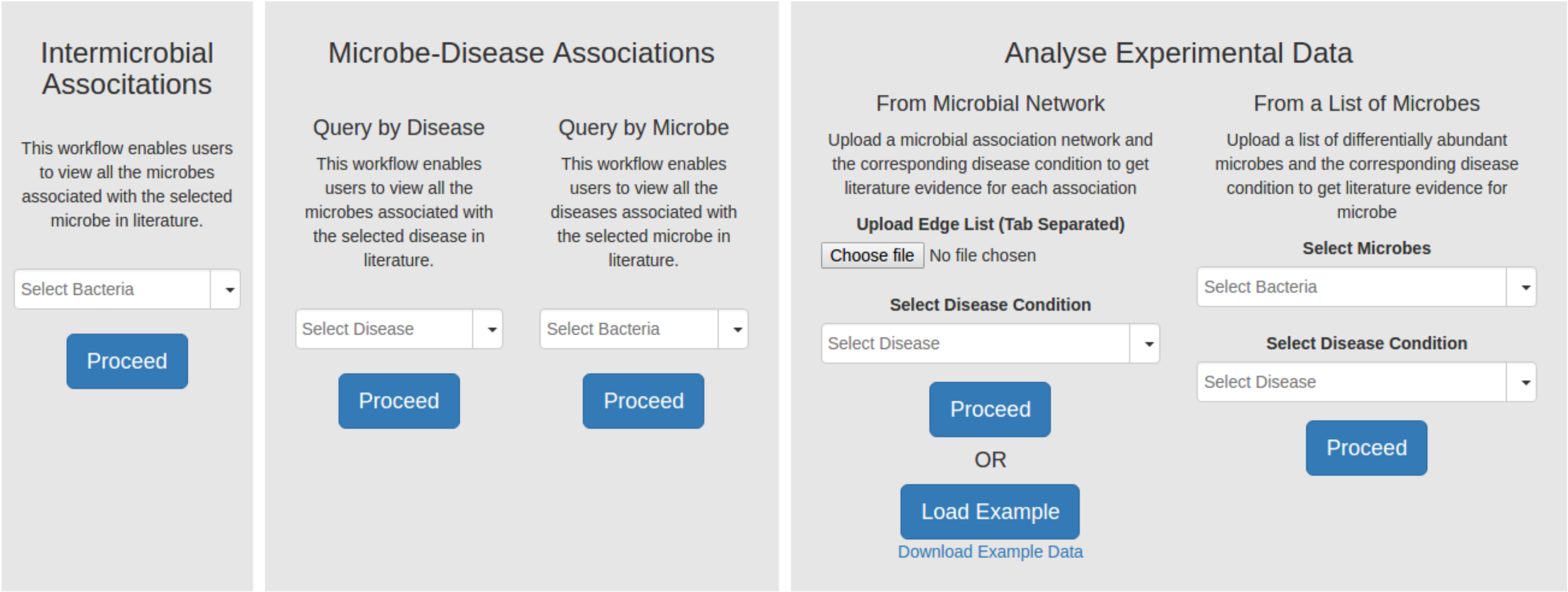

Module 1

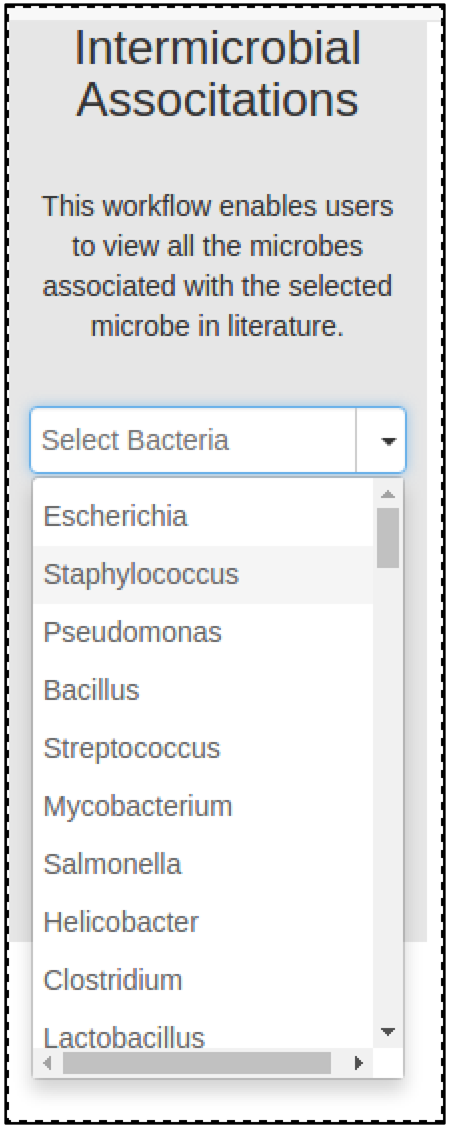

Search for a desired genera name using the searchable dropdown with autosuggest feature

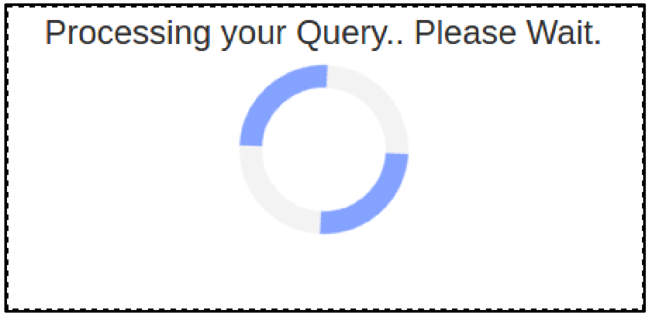

The selected genera along with its associated genera are displayed as a graph with the central node as the ‘query genus’ and connected peripheral nodes as the ‘associated genera’. The size of the node (and labels) are proportionally scaled to the reported number of associations

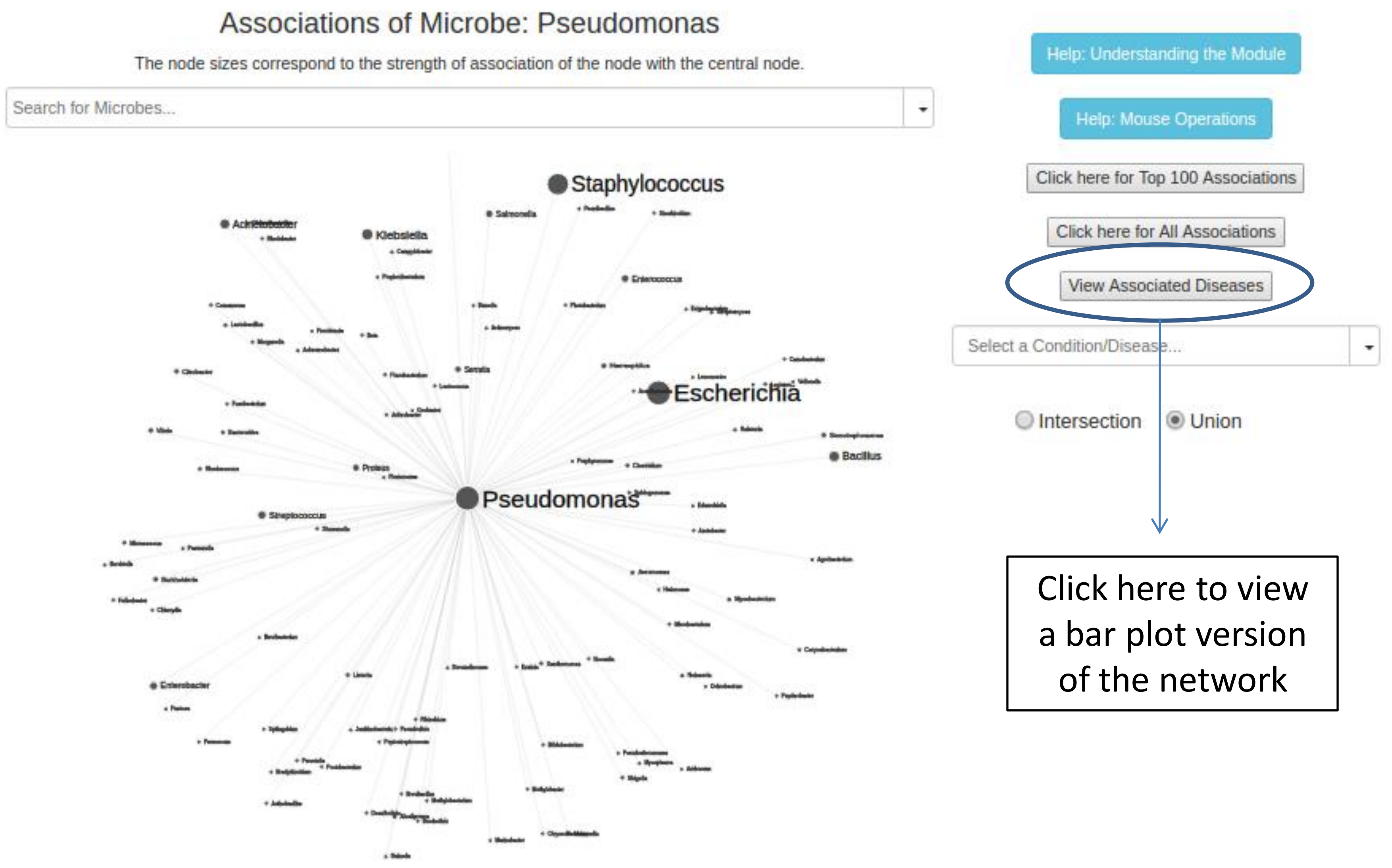

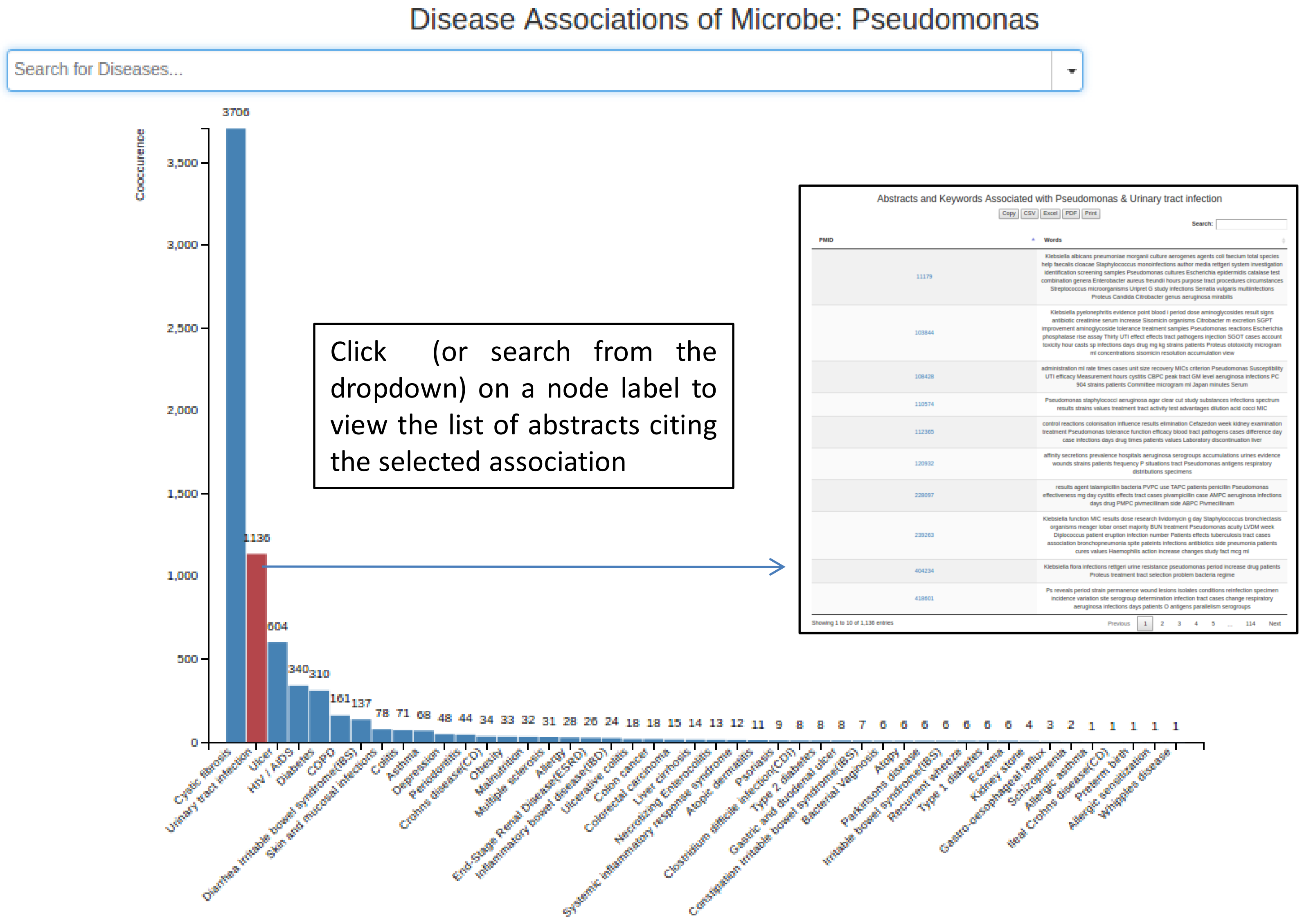

Disease Associations of Microbe: Pseudomonas
Search for Diseases...
Cooccurrence
Click (or search from the dropdown) on a node label to view the list of abstracts citing the selected association
Abstracts and Keywords Associated with Pseudomonas & Urinary tract infection
Cystic fibrosis
Urinary tract infection
Ulcer
HIV / AIDS
Diabetes
COPD

- Search and filter the PMID result tables for a specific keyword
- Export the list in multiple formats
- Most frequent words in the PMID result table can be viewed as a 'word cloud'

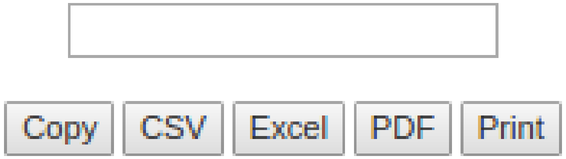

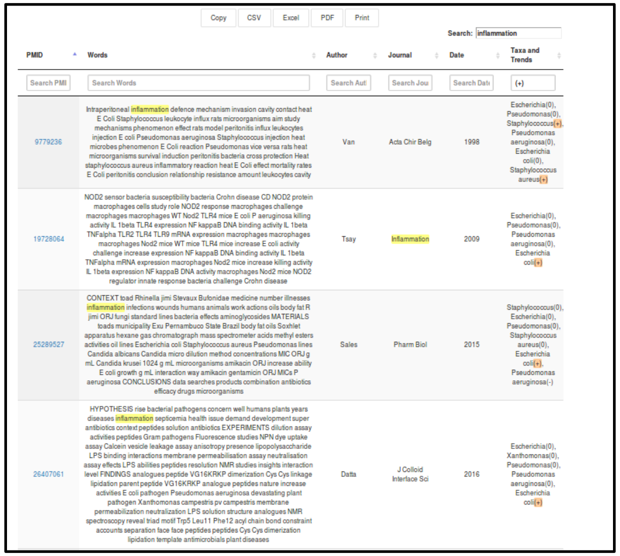

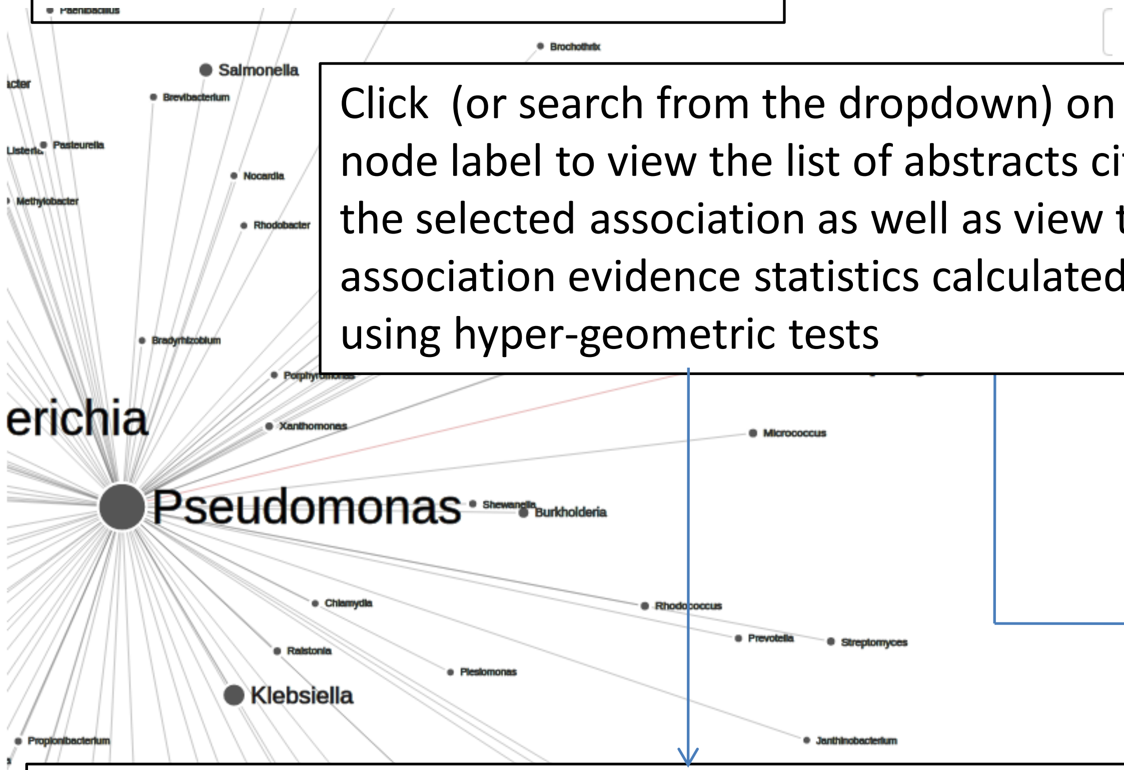

Click (or search from the dropdown) on a node label to view the list of abstracts citing the selected association as well as view the association evidence statistics calculated using hyper-geometric tests

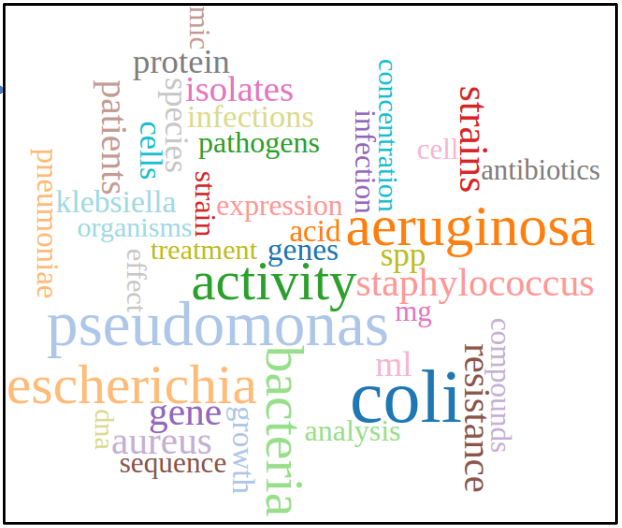

Evidence Statistics

Cooccurence Statistics:

Genus	Number of Abstracts
Pseudomonas AND Escherichia	12850
ONLY Pseudomonas	45577
ONLY Escherichia	162776
NONE	416225

P Values

Statistical Test for Significance of Cooccurence	Uncorrected P value	Bonferroni Corrected P value
One Sided Fisher Exact Test	>0.1	>0.1
Two Sided Fisher Exact Test	<0.0001	<0.0001
Chi Square Test	<0.0001	<0.0001

Select and add one/more diseases and use the ‘Intersection’ radio button to filter and view only those associations present in a selected set of diseases

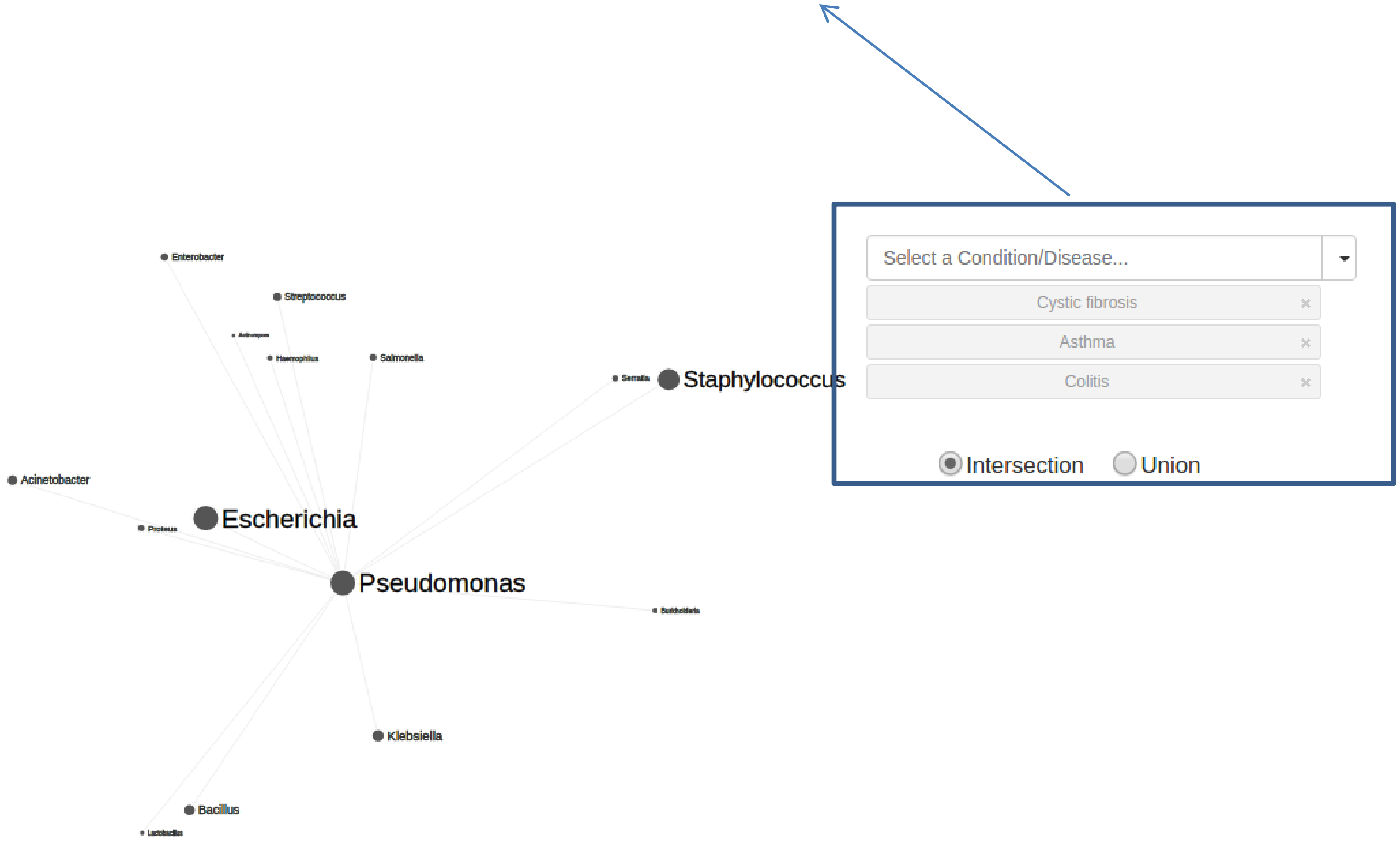

Select and add one/more diseases and use the 'Union' radio button to view all associations present in a selected set of diseases

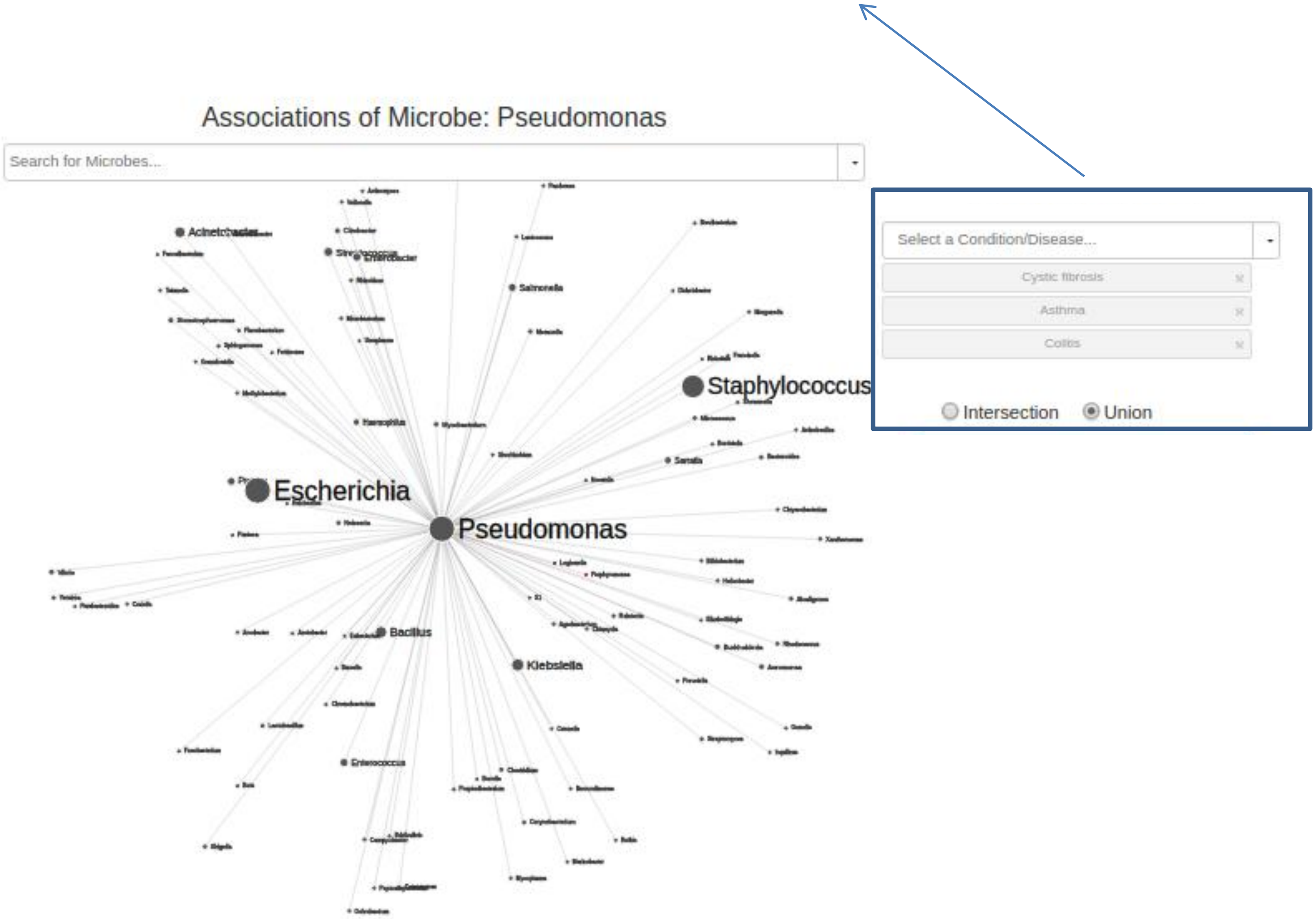

Module 2a

Search for a disease name using the searchable dropdown with autosuggest feature

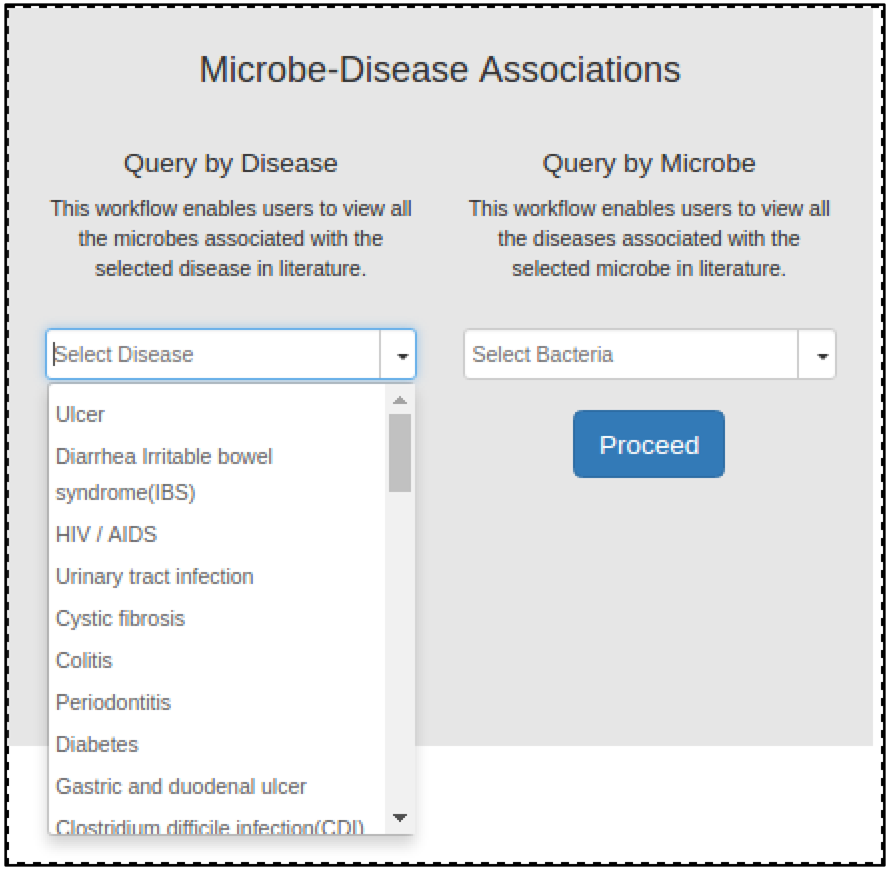

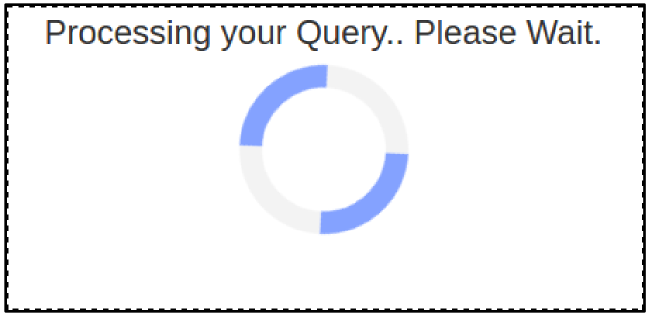

The selected disease along with its associated genera are displayed as a graph with the central node as the 'query disease' and connected peripheral nodes as the 'associated genera'. The size of the node (and labels) are proportionally scaled to the reported number of associations

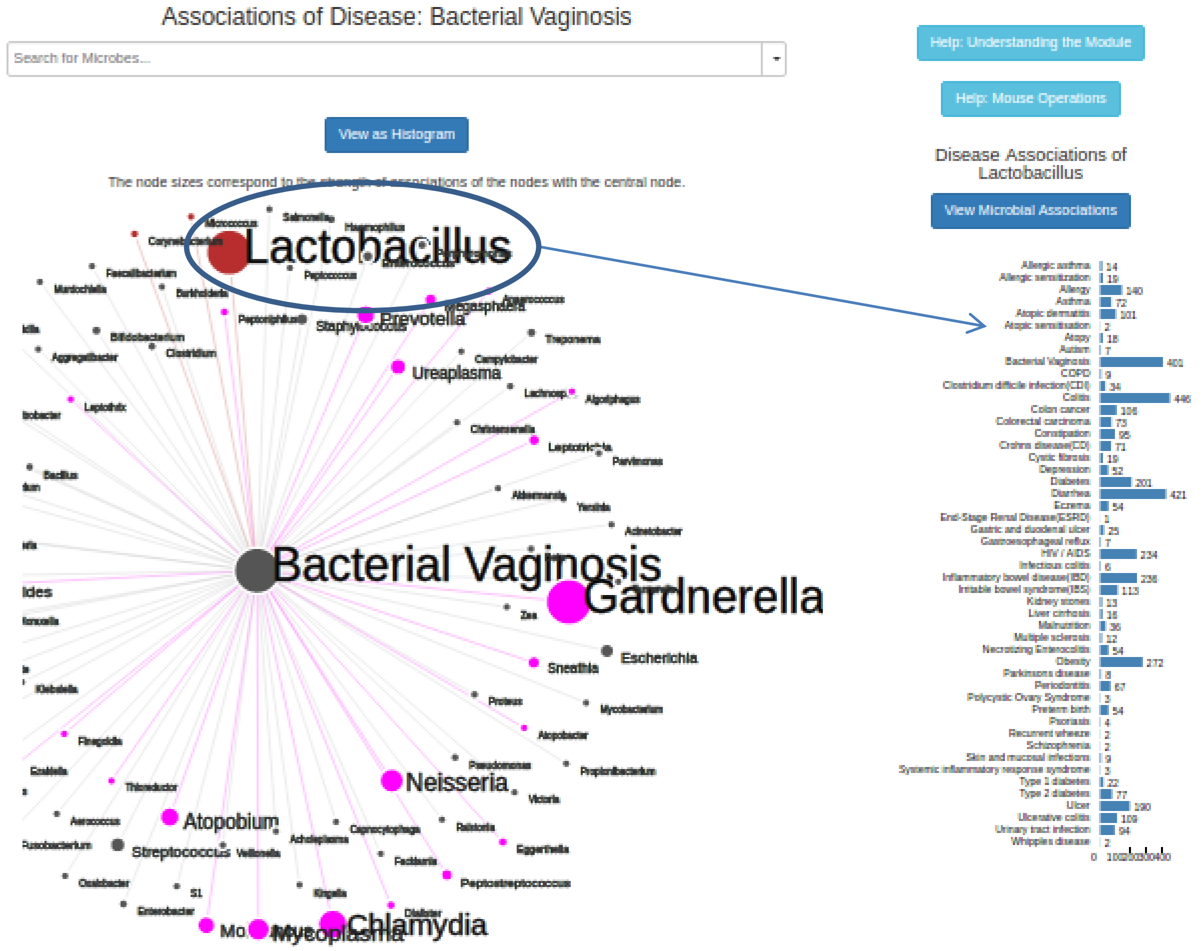

Nodes in pink represents the taxa identified to be significantly associated with the disease

Click (or search from the dropdown) on a node label (genera name) to view the list of other diseases associated with it in form of a bar plot. Additionally the list of abstracts reporting the evidence for the association is also populated.

Clicking on the ‘View as Histogram’ button displays a bar plot for the selected disease and genera associations. The bar plot is sorted by the number of article evidences for the selected association.

Associations of Disease: Bacterial Vaginosis

Search for Microbes...

View as Network

The node sizes correspond to the strength of associations of the nodes with the central node.

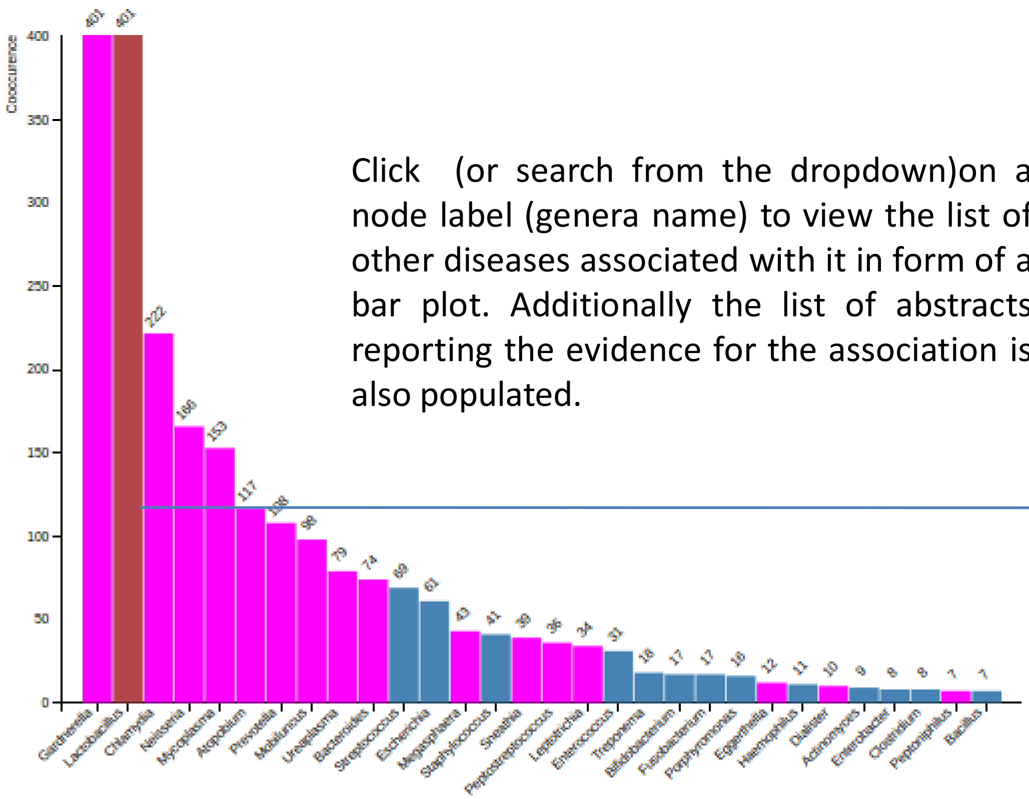

Click (or search from the dropdown)on a node label (genera name) to view the list of other diseases associated with it in form of a bar plot. Additionally the list of abstracts reporting the evidence for the association is also populated.

Help: Understanding the Module

Help: Mouse Operations

Disease Associations of Lactobacillus

View Microbial Associations

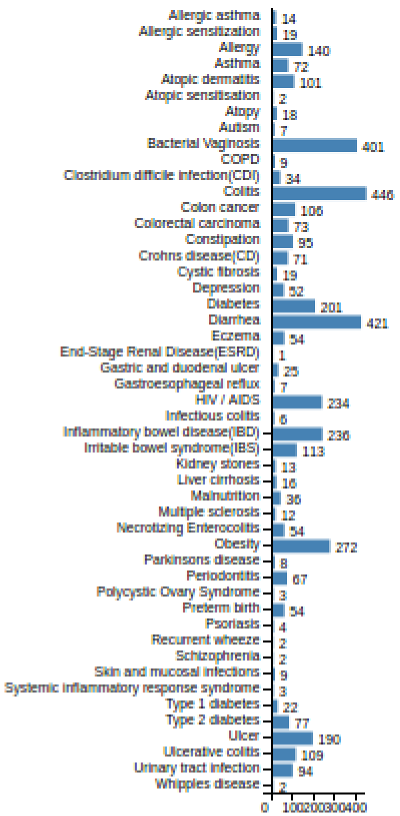

Bars in pink represents the taxa identified to be significantly associated with the disease

Module 2b

Search for a genera name using the searchable dropdown with autosuggest feature

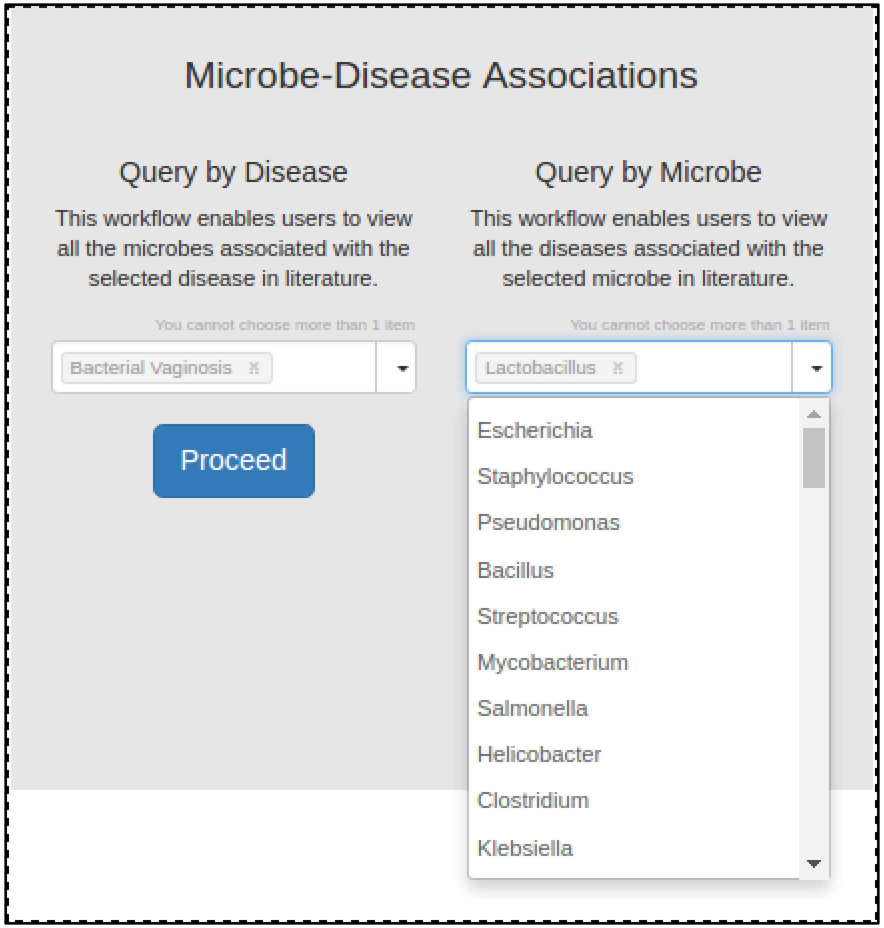

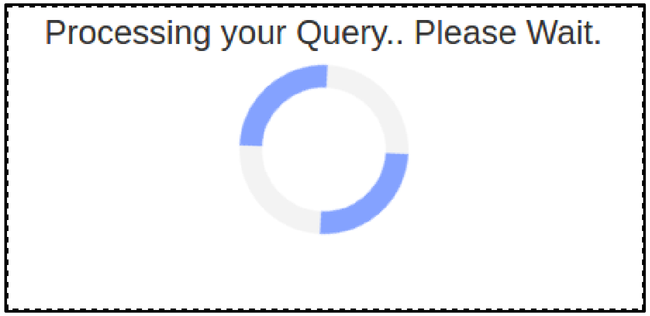

Disease Associations of Microbe: Lactobacillus

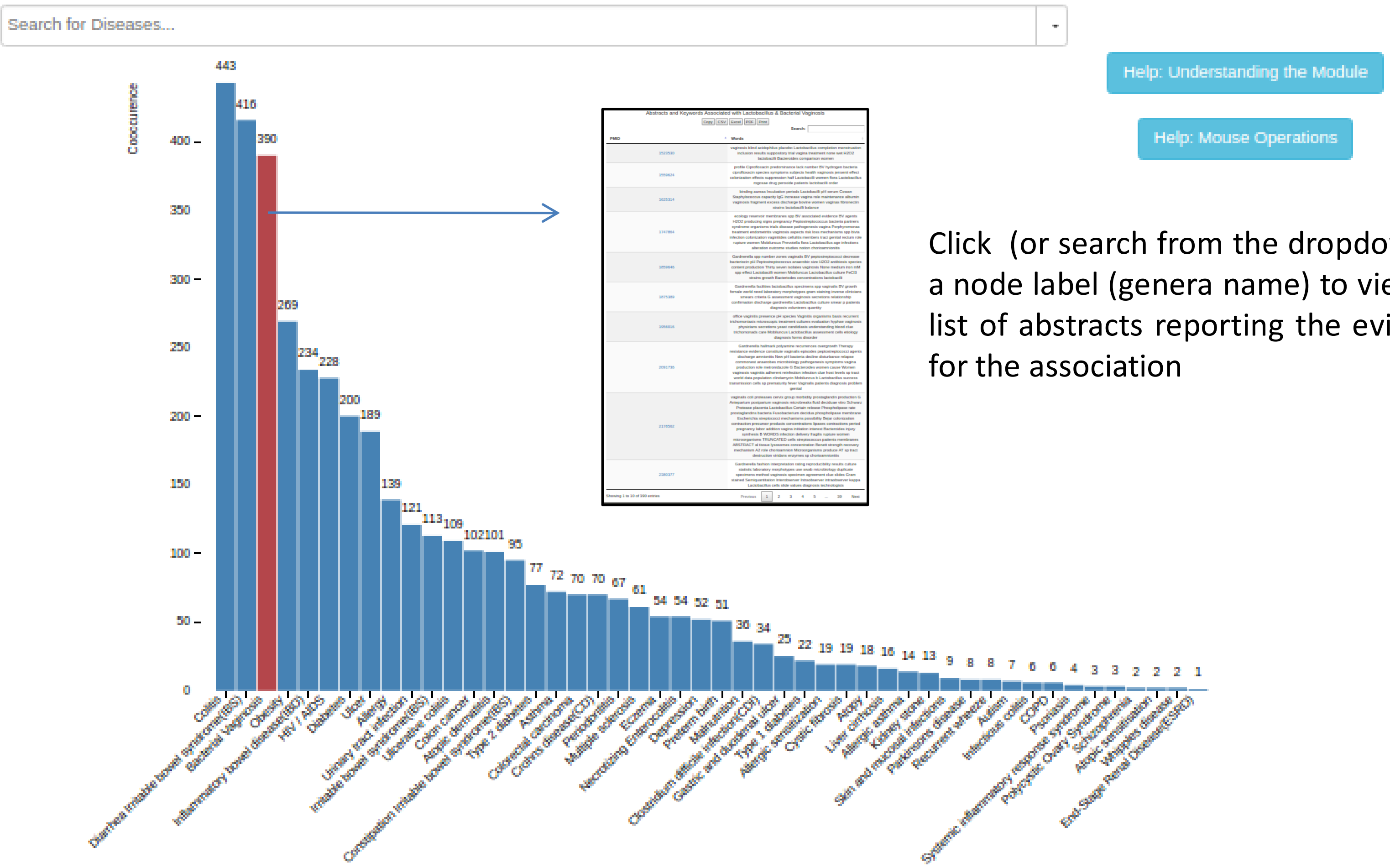

Click (or search from the dropdown)on a node label (genera name) to view the list of abstracts reporting the evidence for the association

Search: (+)

PMID	Words	Author	Journal	Date	Taxa and Trends
Search PMID	HIV	Search Auth	Search Jour	Search Date	Search Taxa
22716110	Beta defensins peptides host invasion skin mucosal surfaces relationships defensin levels colonization colonization vaginosis BV bacteria lactobacilli tract HIV women controls lavage CVL samples women controls PCR assays DNA levels DNA reflective load levels bacteria Lactobacillus species L crispatus L iners L jensenii Lactobacillus levels CVL Levels beta defensins ELISA HIV donors CVL levels rDNA CD4 T cell counts HIV women age HIV women controls CVL DNA levels BV associated bacteria CVL levels Lactobacillus DNAs HIV donors none differences CVL levels hBD 2 hBD 3 HIV women controls correlations levels DNA levels controls HIV women correlations hBD2 hBD 3 Lactobacillus species controls HIV women results HIV infection regulation defenses mucosal sites	Jiang	Curr HIV Res	2012	Beta(0), Lactobacillus(+), Lactobacillus(-), Lactobacillus iners(+), Lactobacillus iners(-)
23894682	contraception methods effects colonization lactobacilli association contraception methods lactobacilli women health study women years age subjects groups basis contraception methods visit day cycle swabs determination Nugent score culture polymerase chain reaction PCR lactobacilli prevalence colony counts rRNA gene expression Lactobacillus strains groups Chi square ANOVA analysis methods Nugent score 0 3 condom group group device IUD p 0 005 prevalence H2O2 producing Lactobacillus condom group IUD group p 0 016 difference colony count standard error SE unit CFU ml H2O2 producing Lactobacillus condom users IUD users p 0 000 rRNA gene expression SE log10copies ml Lactobacillus crispatus condom group IUD group p 0 000 condom use colonization Lactobacillus crispatus vagina vaginosis BV immunodeficiency virus HIV	Ma	PLoS One	2013	Lactobacillus(+), Lactobacillus crispatus(+)
25879811	Women Africa HIV infection tract infections vaginosis BV disruption microbiota HIV infection Risk factors microbiota species polymerase chain reaction data Lactobacillus genus Lactobacillus species bacteria Gardnerella Atopobium vaginae Prevotella bivia Escherichia coli Candida albicans women groups risk HIV reference group adult women risk HIV acquisition species variations subgroups adolescents women women practices sex workers group women combination therapy associations presence quantity bacteria BV Nugent score relation factors importance presence species Kenyan South African Rwandan women differences groups reference women Kenya South Africa Rwandan sex workers women G vaginalis presence women genus log genome equivalents geq ml reference women geq ml L vaginalis L iners women Nugent score exposure presence L crispatus L vaginalis Lactobacillus genus partner months prevalence G L iners CONCLUSIONS composition species countries presence species L crispatus L vaginalis women Nugent score studies Lactobacillus species behavioural Strategies Lactobacillus species TRIAL REGISTRATION study Trial Registration National Health Research Ethics Council South Africa number DOH2709103223	Jespers	BMC Infect Dis	2015	Gardnerella(0), (0), Prevotella(0), Lactobacillus(0), Escherichia(0), vaginae(0), Escherichia coli(0), Lactobacillus iners(+), Lactobacillus vaginalis(+)

Copy | CSV | Excel | PDF | Print

- ➢Search and filter the abstract POS tags for a specific keyword
- ➢ Secondary search can be performed on the individual columns using the 'column search boxes'
- ➢Export the list in multiple formats

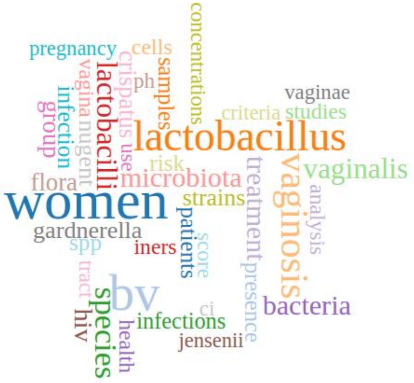

Most Frequent Words and Frequencies

women 1168
bv 1013
lactobacillus 752
vaginosis 639
species 428
vaginalis 348
lactobacilli 346
microbiota 318
bacteria 314
treatment 305

- ➢ The most frequently occurring words in a displayed table can be shown as a 'word cloud' as well as a frequency list of top 10 words
- ➢ A new 'word cloud' can be generated by filtering the table for a custom query

Module 3a

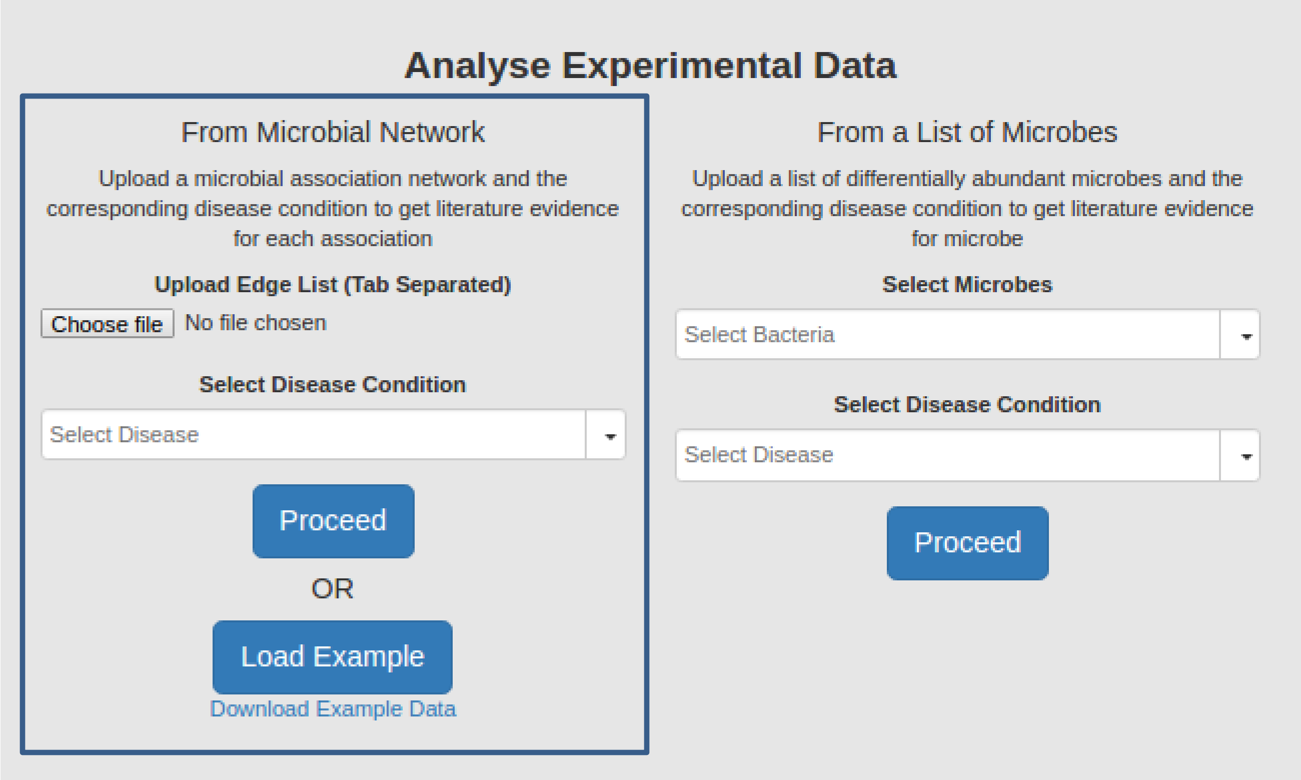

Upload a tab delimited edge list file (see sample file 'Download example data') for microbial associations. Node names should be microbial **genera names** (as in RDP or Green genes database)

The uploaded microbial association network will be displayed showing only the edges whose information is available in the EviMass backend database. An edge can be manually selected (by mouse left click) or by using the dropdown search. Once an edge is selected, a tabulated evidence summary for the associations are displayed. The microbe names in the table are hyper linked to their individual association networks.

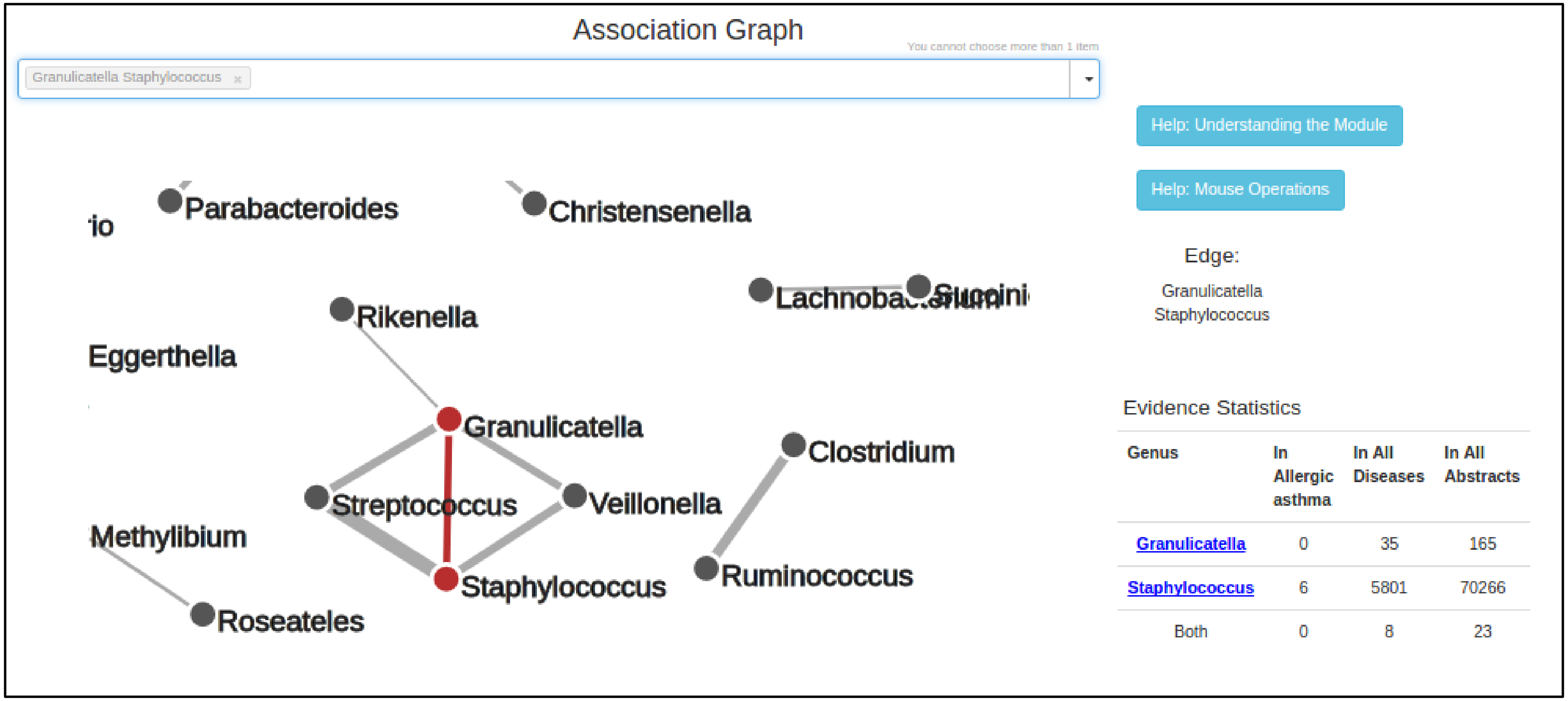

The edge thickness is mapped proportional to the evidence count observed for the microbe-microbe association

In addition to the tabulated evidence indices, the list of abstracts reporting the selected association are also populated as a separate table below the plot. This table contains only the important keywords from each abstract which can be searched (and filtered) to list only the abstracts containing the desired keyword using the search boxes. The table can be explored in multiple formats and the most frequent words can be viewed as a 'word cloud'.

PMID	Words	Author	Journal	Date	Taxa and Trends
Search PM	Search Words	Search Aut	Search Jour	Sear	Search Taxa and Tre
12145727	multicenter study findings sinusitis adults Seventy aerobic anaerobic pathogens patients baseline therapy anaerobes Prevotella species streptococci Fusobacterium species aerobes Streptococcus species Haemophilus influenzae Pseudomonas aeruginosa Staphylococcus aureus Moraxella catarrhalis Recurrences signs symptoms sinusitis anaerobes aerobes counts anaerobes 10 cfu mL role Granulicatella species cases sinusitis time	Finegold	Clin Infect Dis	2002	Fusobacterium(0), Prevotella(0), Moraxella(0), Granulicatella(0), Haemophilus(0), Staphylococcus(0), Streptococcus(0), Pseudomonas(0), Pseudomonas aeruginosa(0), Staphylococcus aureus(0), Haemophilus influenzae(0)
12808082	Chronic sinusitis inflammatory condition role infection Bacteria sinuses role chronicity inflammation objective study bacteria samples sinusitis diversity present Washes tissue samples sinus surgery patients sinusitis PCR amplification rDNA primer pairs samples presence bacteria fungi culture methods bacteria rDNA sinus samples patients sinus samples PCR culture methods PCR culture Thirteen species Abiotrophia defectiva Enterococcus avium Eubacterium sp Granulicatella Neisseria sp Prevotella sp Pseudomonas aeruginosa Serratia Staphylococcus aureus Stenotrophomonas maltophilia Streptococcus gordonii Streptococcus mitis Streptococcus oralis Streptococcus sp Fungi patient Streptococcus mitis Streptococcus oralis patient Pseudomonas aeruginosa sinus cavity PCR primers results bacteria half sinusitis cases	Paju	J Med Microbiol	2003	Staphylococcus(0), Enterococcus(0), Granulicatella(0), Pseudomonas(0), Serratia(0), Stenotrophomonas(0), Streptococcus(0), Neisseria(0), Abiotrophia(0), Eubacterium(0), Prevotella(0), Serratia marcescens(0), Pseudomonas aeruginosa(0), Stenotrophomonas maltophilia(0), Staphylococcus aureus(0), Abiotrophia defectiva(0), Streptococcus sp(0)

Module 3b

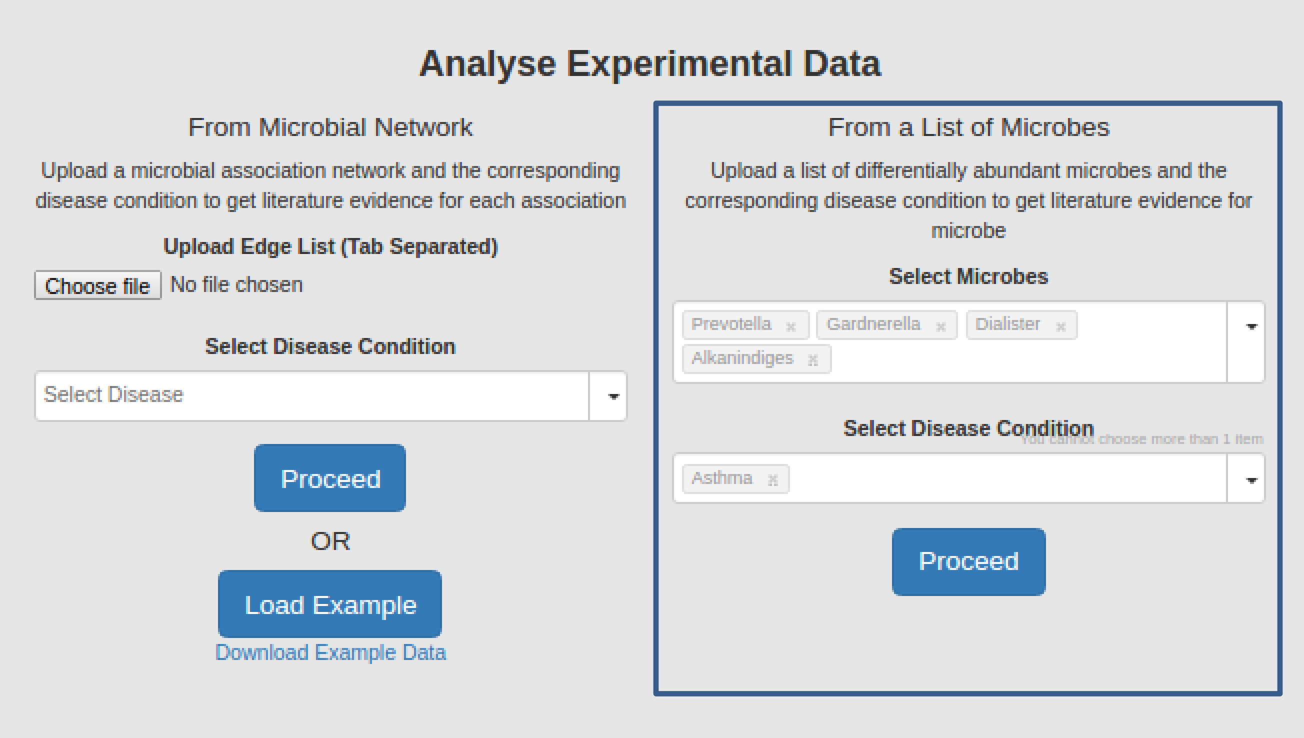

Enter a list of microbial genera reported in an experiment along with the disease name to view the literature evidences supporting the microbe-disease associations.

The selected disease along with its associated genera are displayed as a graph with the central node as the ‘query disease’ and connected peripheral nodes as the ‘associated genera’. The size of the node (and labels) are proportionally scaled to the reported number of associations. An enrichment analysis is also performed on the uploaded microbe list specific to the disease and the p-value for enrichment is reported.

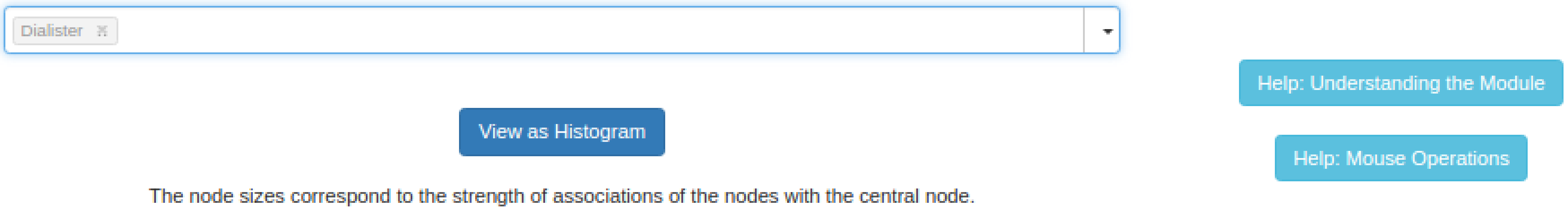

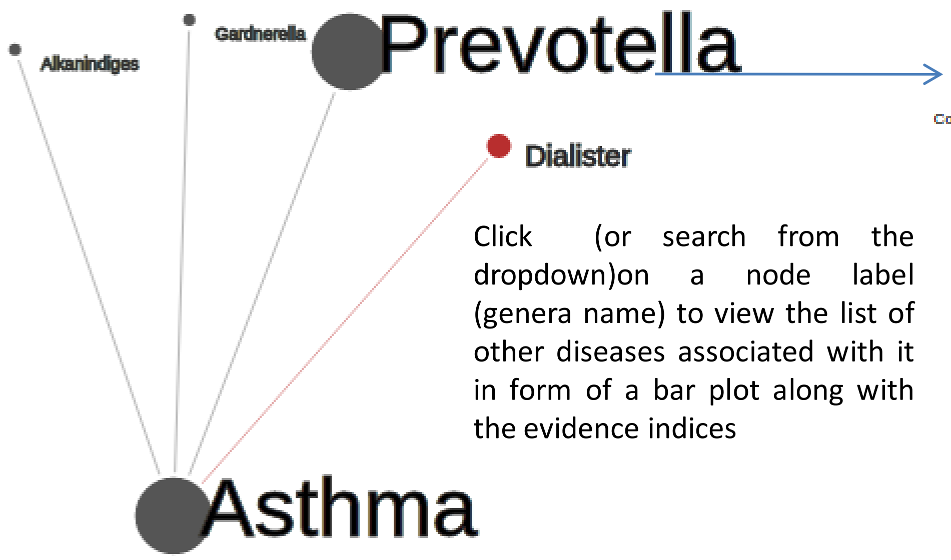

Click (or search from the dropdown)on a node label (genera name) to view the list of other diseases associated with it in form of a bar plot along with the evidence indices

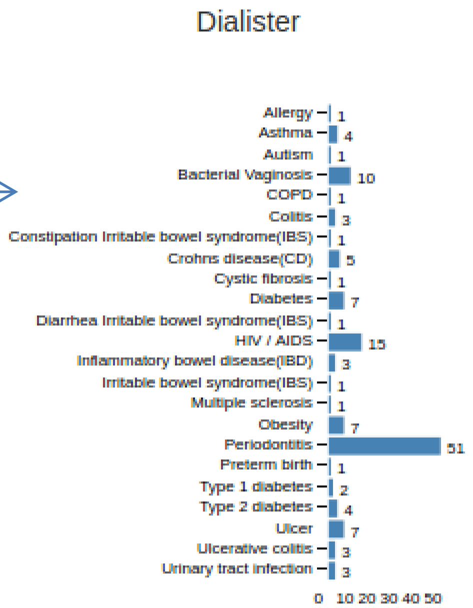

Clicking on the 'View as Histogram' button displays a bar plot for the selected disease and genera associations. The bar plot is sorted by the number of article evidences for the selected association.

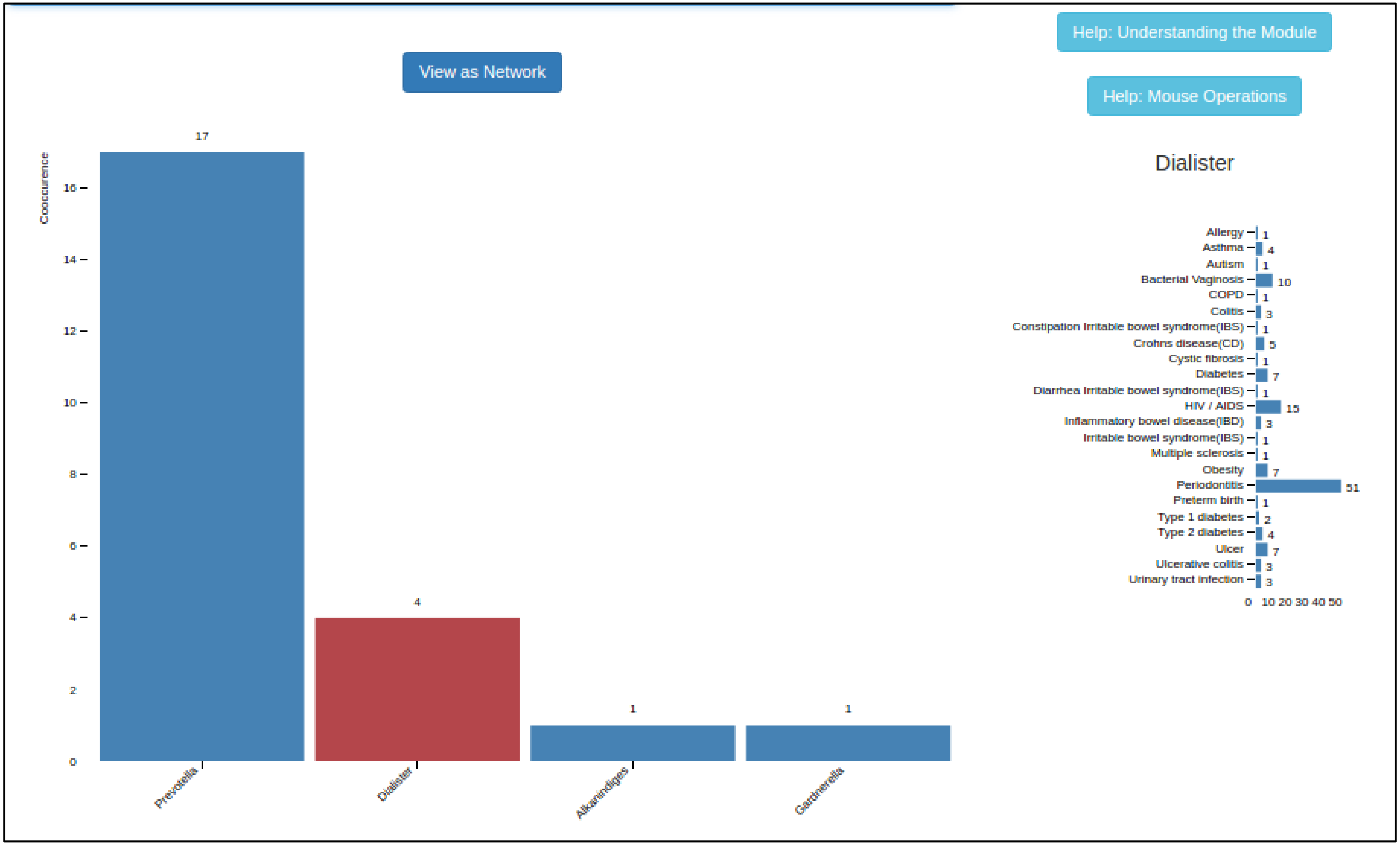

The list of abstracts reporting the selected association are also populated as a separate table below the plot. This table contains only the important keywords from each abstract which can be searched (and filtered) to list only the abstracts containing the desired keyword using the search box. The table can be explored in multiple formats.

PMID	Words	Author	Journal	Date	Taxa and Trends
Search PMI	Search Words	Search Autl	Search Jou	Search Dat	Search Taxa and T
20052417	environment infancy protects infections exacerbations airway microbiota levels adult patients asthma condition COPD controls lavage children controls FINDINGS rRNA sequences subjects species tree mean genomes cm surface Pathogenic Proteobacteria Haemophilus spp bronchi adult asthmatics patients COPD controls increases Proteobacteria children Bacteroidetes Prevotella spp controls adult asthmatics COPD patients results tree microbiota microbiota airways	Hilty	PLoS One	2010	Prevotella(0), Haemophilus(0)
23711849	interaction microbiota diseases bacteria inception progression asthma others protection asthma mechanisms bacteria harm regard study bacteria effects functions eosinophils effector cells inflammation asthma Eosinophils adult volunteers Percoll density gradient centrifugation bead selection microbeads kinds bacteria asthma Staphylococcus aureus SA Haemophilus influenzae HI Prevotella sp PS effects secretion neurotoxin EDN generation superoxides production cytokines chemokines SA HI PS EDN release manner Superoxide generation species SA production eosinophils HI PS cytokine HI PS SA CONCLUSIONS Bacteria effects eosinophils results SA exacerbation HI PS inhibition inflammation asthma	Hosoki	Int Arch Allergy Immunol	2013	Prevotella(0), Haemophilus(0), Staphylococcus(0), Staphylococcus aureus(0), Haemophilus influenzae(0)
25179236	studies airways colonization commensal microbiota Prevotella spp properties bacteria respiratory system respiratory response commensal Prevotella strains Prevotella melaninogenica Prevotella nanceiensis Prevotella salivae Proteobacteria lungs patients disease COPD Haemophilus influenzae B Haemophilus influenzae Moraxella catarrhalis commensal Prevotella spp Proteobacteria differences capacities murine lung cells vitro vivo mice H influenzae Toll like receptor TLR2 inflammation airway neutrophilia expression cytokine chemokine profile lung tissue lung immunopathology comparison P nanceiensis airway inflammation lung pathology airway response bacteria P nanceiensis findings inflammatory properties airway commensal Prevotella spp colonization bacteria respiratory immune system	Larsen	Immunology	2015	Prevotella(0), Haemophilus(0), Moraxella(0), Prevotella nanceiensis(0), Haemophilus influenzae(0), Prevotella melaninogenica(0)
